# In Silico Comparative Genomic Analysis Revealed a Highly Conserved Proteolytic System in *Lactobacillus delbrueckii*

**DOI:** 10.3390/ijms241411309

**Published:** 2023-07-11

**Authors:** Mariano Elean, Leonardo Albarracin, Julio Villena, Haruki Kitazawa, Lucila Saavedra, Elvira M. Hebert

**Affiliations:** 1 Reference Centre for Lactobacilli (CERELA-CONICET), Tucumán 4000, Argentina; melean@cerela.org.ar (M.E.); lalbarracin@herrera.unt.edu.ar (L.A.); jcvillena@cerela.org.ar (J.V.); lucila@cerela.org.ar (L.S.); 2 Food and Feed Immunology Group, Laboratory of Animal Food Function, Graduate School of Agricultural Science, Tohoku University, Sendai 980-8572, Japan; 3 Livestock Immunology Unit, International Education and Research Centre for Food and Agricultural Immunology (CFAI), Graduate School of Agricultural Science, Tohoku University, Sendai 980-8572, Japan

**Keywords:** cell envelope proteinase, *Lactobacillus delbrueckii*, proteinase activity, PrtL, PrtB, lactic acid bacteria

## Abstract

*Lactobacillus delbrueckii*, the type species of the genus *Lactobacillus*, is widely recognized as the primary starter culture in the dairy industry due to its proteolytic activity, which enables it to growth in milk. In this study, a comprehensive genomic analysis of the proteolytic system was conducted on *L. delbrueckii* strains. The analysis included 27 genomes of *L. delbrueckii*, with a specific focus on the key enzyme involved in this system, the cell envelope-associated proteinase (CEP). The amino acid sequences, as well as the protein-structure prediction of the CEPs, were compared. Additionally, syntenic analysis of the genomic locus related to the CEPs revealed high conservation in *L. delbrueckii* subsp. *bulgaricus* strains, while *L. delbrueckii* subsp. *lactis* strains exhibited greater variability, including the presence of insertion sequences, deletions, and rearrangements. Finally, the CEP promoter region and putative regulatory elements responsible for controlling the expression of the proteolytic system in lactobacilli were investigated. Our genomic analysis and *in silico* characterization of the CEPs contribute to our understanding of proteolytic activity and the potential applications of these lactic acid bacteria in the dairy industry. Further research in this area will expand our knowledge and potential practical uses of these findings.

## 1. Introduction

Lactic acid bacteria (LAB) have a significant impact on the food industry, fulfilling crucial roles as probiotics, postbiotics, and starter cultures in the production of fermented foods. The genus *Lactobacillus* includes the type species *Lactobacillus delbrueckii* [1,2] which consists of six recognized subspecies: *Lactobacillus delbrueckii* subsp. *delbrueckii*, *L. delbrueckii* subsp. *bulgaricus*, *L. delbrueckii* subsp. *indicus*, *L. delbrueckii* subsp. *lactis*, *L. delbrueckii* subsp. *Sunkii*, and *L. delbrueckii* subsp. *jakobsenii*. *L. delbrueckii* subsp. *lactis* was the first subspecies isolated by G. Leichmann (1896) from a dairy product [1]; this subspecies is used as a starter culture for the elaboration of a variety of fermented dairy products, such as fermented sour milks, mozzarella, and Swiss and Italian cheeses [1]. *L. delbrueckii* subsp. *bulgaricus,* which was initially isolated from a Bulgarian milk (1919) [1], is used in combination with *Streptococcus thermophilus* as a starter culture for commercial yogurt production, as well as in the manufacture of Swiss and Italian cheeses. On the other hand, *L. delbrueckii* subsp. *indicus* was first isolated from a dairy product in India [1], while *L. delbrueckii* subsp. *delbrueckii*, *L. delbrueckii* subsp. *sunkii*, and *L. delbrueckii* subsp. *jakobsenii* were isolated from cereal, vegetable products, and malted sorghum wort, respectively [1]. *L. delbrueckii* subsp. *lactis* and *L. delbrueckii* subsp. *bulgaricus* exemplify the industrial significance of this species mainly in the production of fermented milk products, whereas *L. delbrueckii* subsp. *delbrueckii* finds its primary application in fermented vegetable products [2].

The ideal milk starter culture should rapidly and dependably produce lactic acid [3,4]. LAB have complex nutritional requirements for growth and are auxotrophic for a large number of amino acids [5,6]. Milk contains limited concentrations of free essential amino acids, making the sustained growth of LAB in this substrate dependent on the presence of a complete proteolytic system capable of utilizing casein as a nitrogen source [3,4]. The LAB proteolytic system consists of: (I) one or more cell envelope-associated proteinases (CEPs), which are responsible for the initial hydrolysis of casein into oligopeptides; (II) amino acid and peptide transport systems; and (III) intracellular peptidases, which further break down oligopeptides into smaller peptides and amino acids [7,8]. The CEP enzyme plays a pivotal role in this proteolytic system by initiating the hydrolysis of milk proteins [7,8,9,10].

Extensive research has focused on the proteolytic system of *Lactococcus* compared to that of lactobacilli among LAB [11,12]. However, there has been increasing interest in studying the proteolytic systems of specific *Lactobacillus* species, particularly *L. delbrueckii* subsp. *bulgaricus*, and *L. delbrueckii* subsp. *lactis*. These species play a crucial role in the dairy industry as starter cultures for fermented milk products, and their proteolytic system is not only vital for their growth, but also contributes to the development of the organoleptic characteristics of the final products. Moreover, there has been a growing interest in the potential of these strains to produce bioactive peptides with health-promoting properties. In this sense, a comprehensive genomic analysis of the proteolytic system from *L. delbrueckii* subsp. *lactis* CRL 581 was conducted in our lab [7,13]. The analysis identified genes encoding the CEP, two peptide transport systems, and sixteen peptidases. Furthermore, a previous evaluation of proteolytic activity was performed on 36 strains belonging to the *L. delbrueckii* species, demonstrating that this activity is strain-specific [4]. The analysis encompassed strains from the two aforementioned subspecies, *L. delbrueckii* subsp. *bulgaricus* and *L. delbrueckii* subsp. *lactis*, as well as the *L. delbrueckii* subsp. *delbrueckii* subspecies, taking into account the immunogenic potential of some strains belonging to this subspecies [14]. Among the tested strains, *L. delbrueckii* subsp. *bulgaricus* ATCC 11842 exhibited the lowest activity, while *L. delbrueckii* subsp. *lactis* CRL 581 showed the highest activity [4]. Notably, the proteolytic activity of the CRL 581 strain was approximately 18 times higher than that of the ATCC 11842.

Considering the significant variations observed in the proteolytic activity among different strains of *L. delbrueckii*, we conducted a comprehensive analysis of 26 completed genomes available on the NCBI database plus the genome of *L. delbrueckii* subsp. *lactis* CRL 581, with a specific focus on the genes involved in the proteolytic system. Initially, the phylogenetic relationship of the 27 strains was determined, and their subspecies were confirmed using average nucleotide identity (ANIm) and multilocus sequence analysis (MLSA). Next, a pangenomic analysis was performed to identify genes associated with peptidases and CEPs (*pep* and *prt*, respectively) across the different strains. Taking into account the variability in the proteinase activity among several strains of *L. delbrueckii*, a comparative analysis of their primary sequences and tridimensional structures was performed. Furthermore, an analysis on the promoter and upstream regions corresponding to the *prt* genes, along with the presence of transcriptional regulators associated with the lactobacilli proteolytic system, was carried out.

## 2. Results

### 2.1. Phylogenetic Analysis

The ANIm analysis of the 27 selected strains (Supplementary Figure S1) revealed identity percentages greater than 97%. The resulting ANI values ranged from 97.28 to 99.98%, falling within the generally recommended ranges for prokaryotic species [15,16]. Notably, the strains were categorized into two distinct groups. The first group consisted exclusively of strains belonging to the species *L. delbrueckii* subsp. *bulgaricus*, while the second group comprised *L. delbrueckii* subsp. *delbrueckii* and *L. delbrueckii* subsp. *lactis* strains. Surprisingly, among the 15 strains of *L. delbrueckii* subsp. *bulgaricus* studied, the strain ND02 was grouped with the second cluster, demonstrating a high degree of identity (99.97%) with *L. delbrueckii* subsp. *lactis* CIDCA133. Additionally, both strains shared an identical plasmid (100% identity and 0 gaps), annotated as CP002342.1 and CP065514.1 in ND02 and CIDCA133, respectively. These findings suggest that the strain ND02 should be classified as belonging to the *L. delbrueckii* subsp. *lactis* subspecies rather than *L. delbrueckii* subsp. *bulgaricus*.

**Figure 1:**
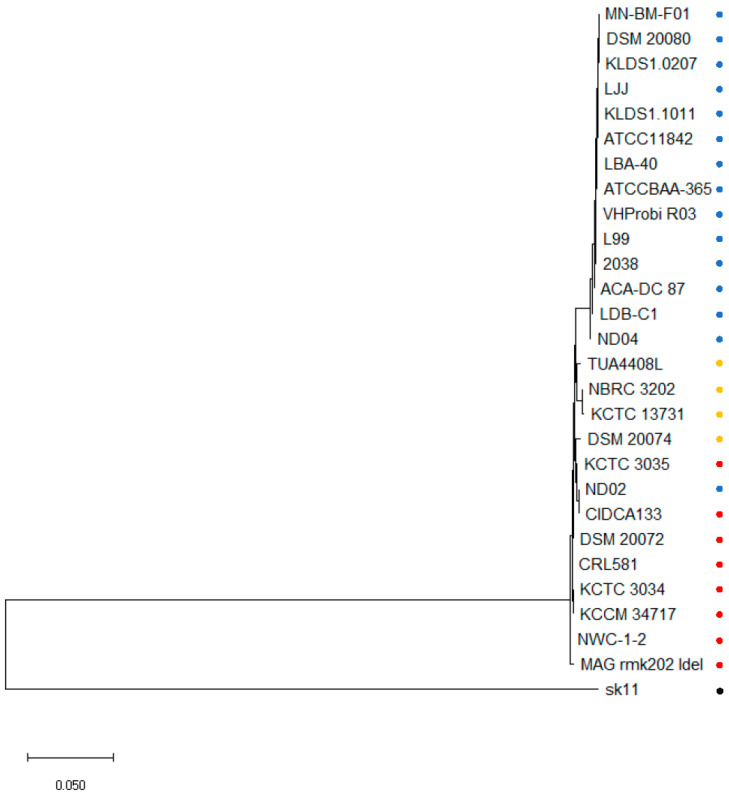
Multilocus sequence analysis (MLSA) of *Lactobacillus delbrueckii* strains. The subspecies are indicated by colored circles: *L. delbrueckii* subsp. *bulgaricus* in blue, *L. delbrueckii* subsp. *lactis* in red and *L. delbrueckii* subsp. *delbrueckii* in green. *Lactococcus lactis* SK11 (black circle) was used as an outgroup.

To further elucidate the subspecies to which strain ND02 belongs, an MLSA was conducted. The 27 selected strains were effectively segregated into two distinct clusters (Figure 1). The first cluster exclusively comprised strains belonging to *L. delbrueckii* subsp. *bulgaricus*, with the exception of the ND02 strain. In contrast, the second cluster encompassed strains of *L. delbrueckii* subsp. *delbrueckii* and *L. delbrueckii* subsp. *lactis*. Moreover, no discernible differences were observed between the strain ND02s and CIDCA133, both of which were positioned within the second cluster.

### 2.2. In Silico Analysis of the Proteolytic System

#### 2.2.1. Peptidases

The pangenome analysis of the 27 strains revealed a total of 2818 genes. Among them, the core-genome consisted of 894 genes, the softcore-genome (25 genomes) comprised 326 genes, the shell-genome (3–25 genomes) contained 996 genes, and the cloud-genome (1–2 genomes) included 602 genes. Within the cloud-genome, 309 genes were found to be unique to specific strains. 

Considering the proteolytic system of LAB, we identified several peptidase genes, *pepC*, *pepD*, *pepF*, *pepI*, *pepM*, *pepN*, *pepO*, *pepQ*, *pepR*, *pepT*, *pepV*, and *pepX,* in the core-genome, (Table 1). The genes *pepA*, *pepG/E*, and *pepP* were part of the softcore-genome but were absent in the KCTC 13731, DSM 20072, and 2038 strains, respectively (Table 1). The *pepL* gene was only found in the strains CIDCA133, CRL 581, DSM 20074, KCCM 34717, KCTC 13731, KCTC 3034, MAG_rmk202_Idel, NBRC 3202, ND02, and TUA4408L. Additionally, all evaluated strains possessed the *pcp* (OG0000669) and *dpp* (OG0000898) genes, encoding pyroglutamyl-peptidase I and dipeptidyl-peptidase VI, respectively. Furthermore, seven putative peptidases genes (OG0000061, OG0000393, OG0000665, OG0000907, OG0000926, OG0000928, and OG0000957) were identified, although their specific assignments were not determined.

#### 2.2.2. Cell Envelope-Associated Proteinase (CEP)

**Figure 2:**
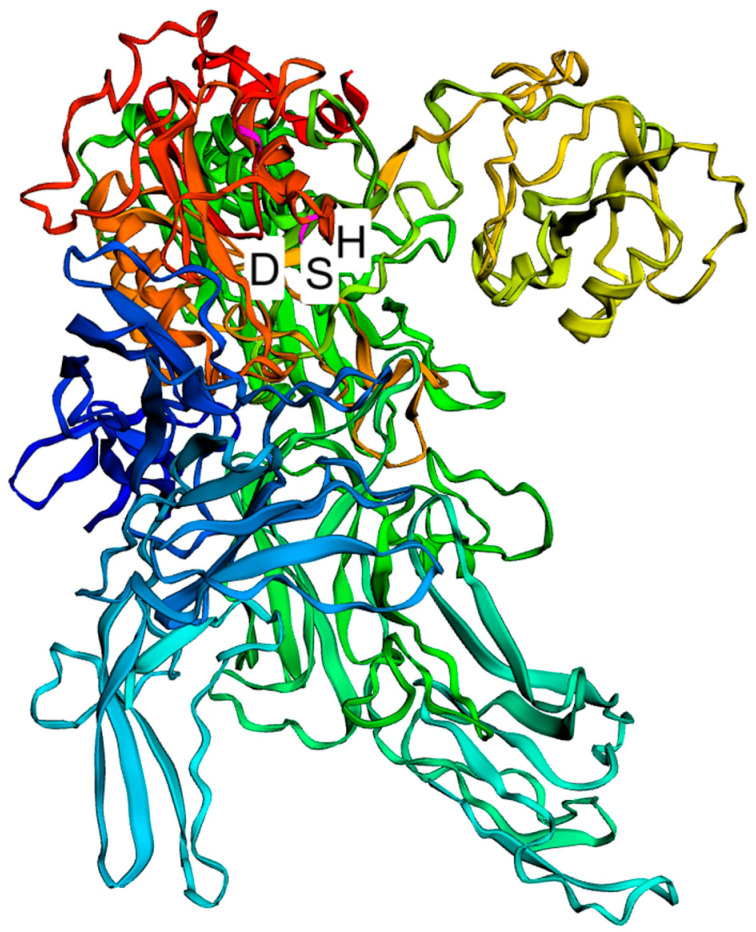
Structure prediction model of the proteinase from *L. delbrueckii* subsp*. lactis* CRL 581. A cartoon representation of a protein monomer shown in rainbow colors from red at the N-terminus to blue at the C-terminus. The active site of the enzyme contains the catalytic triad of amino acids: D, H, and S.

All *L. delbrueckii* strains evaluated in this study bear only one *prt* gene in each genome, named *prtB* in the strains belonging to the *L. delbrueckii* subsp. *bulgaricus* subspecies and *prtL* in the *L. delbrueckii* subsp. *lactis* and *L. delbrueckii* subsp. *delbrueckii* subspecies. This *prt* gene encodes a CEP, which generally possesses an amino acid sequence of about 1924 residues. The enzyme is synthesized as a preproprotein and matures after the removal of the PrePro domain [17], which consists of a putative 34-amino-acid signal sequence and a 158-amino-acid prosequence, with the predicted cleavage site between T192 and D193. Therefore, the mature proteinase contains approximately 1733 residues. The N-terminal region of the mature PrtL proteinase (497 amino acids) from the CRL 581 strain corresponds to the catalytic domain (PR), which exhibits similarity to subtilisin-type serine proteinases known as subtilases. Thus, these proteinase sequences can be classified within this proteinase family [17]. This catalytic domain is characterized by a catalytic triad composed of D30, H94, and S425 (Figure 2). However, in *L. delbrueckii* subsp*. delbrueckii* KCTC 13731, *L. delbrueckii* subsp*. delbrueckii* NBRC 3202, and *L. delbrueckii* subsp*. lactis* KCTC 3035, there is a premature termination codon, resulting in a truncated mature proteinase of approximately 155, 155, and 843 residues, respectively (Supplementary Figure S2). The proteinase from the KCTC 13731 and NBRC 3202 strains would not be functional since they lack the S425 amino acid, which is part of the catalytic triad and essential for enzyme activity (Supplementary Figure S2). Similarly, the putative proteinase in the KCTC 3035 strain lacks the D30 and H94 amino acids that are crucial components of the active site of the enzyme (Supplementary Figure S2).

**Figure 3:**
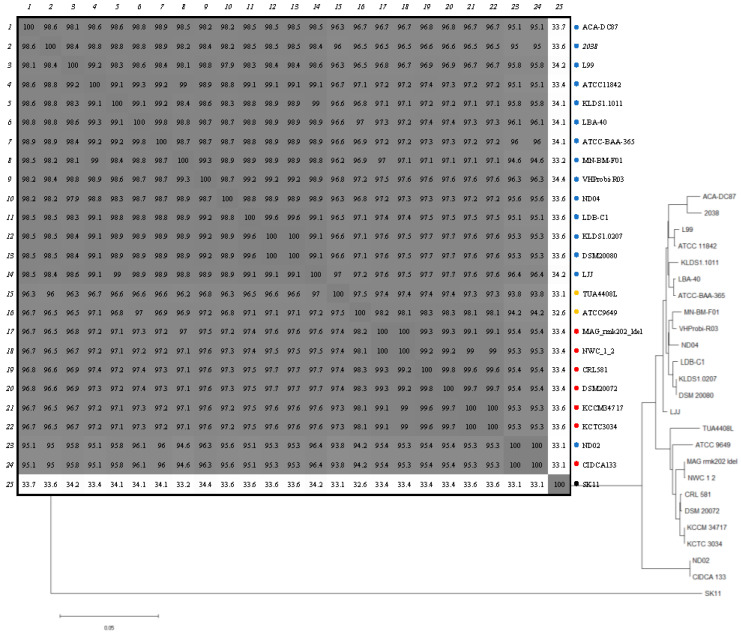
Phylogenetic analysis and identity percentage of proteinases in *L. delbrueckii* subspecies. Evolutionary relationships were inferred using the method of joining neighbors. Evolutionary distances were calculated using the p distance method and are expressed as the number of different amino acids per site. The PrtP proteinase from *Lactococcus lactis* subsp. *cremoris* SK11 was used as an outgroup. The percentages of identity between the proteinases of each strain are shown in the table. *L. delbrueckii* subsp. *bulgaricus* strains are marked with a blue dot, *L. delbrueckii* subsp. *delbrueckii* strains with a green dot, and *L. delbrueckii* subsp. *lactis* with a red dot.

A phylogenetic analysis of CEPs from different *L. delbrueckii* strains is shown in Figure 3. The proteinase sequences were clustered into three main groups. The first group consisted of putative PrtB sequences, which corresponded to the 14 strains of *L. delbrueckii* subsp. *bulgaricus*. The PrtB sequences exhibited more than 98.8% identity among them. The second cluster included proteinases from the strains ND02 and CIDCA133, sharing the same predicted proteinase with 100% identity. Finally, the remaining strains belonging to *L. delbrueckii* subsp. *delbrueckii* (TUA4408L and ATCC 9649) and *L. delbrueckii* subsp. *lactis* (six strains) were clustered together. The analysis of alignments revealed that position 282 of the CEPs in *L. delbrueckii* subsp. *lactis* and *L. delbrueckii* subsp. *delbrueckii*, referred to as PrtL, is characterized by a serine residue, while PrtB (specific to *L. delbrueckii* subsp *bulgaricus*) exhibits a cysteine residue at the same position (Supplementary Figures S3 and S4). Similarly to PrtL, the ND02 strain also displays a serine residue at position 282 (position 90 in the mature CEP). An analysis carried out using Missense3D [18] to predict the structural changes introduced by an amino acid substitution showed that this substitution did not alter the conformational structure of the enzyme (Supplementary Figure S3).

**Figure 4:**
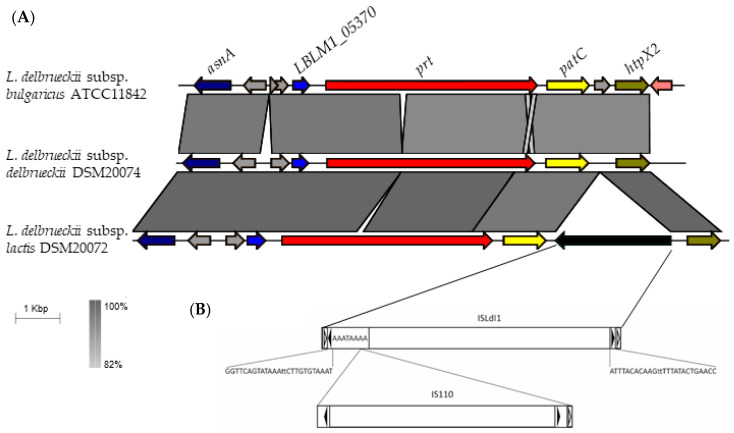
Synteny analysis of the *prt* region in *L. delbrueckii* type strains. (**A**). Comparison of synteny in *L. delbrueckii* strains for the *prt* region (14 Kb). The following genes are represented: aspartate ammonium lyase *asnA* (dark blue), acetyltransferase *LBLM1_05370* (blue), proteinase *prt* (red), cystathionine beta-lyase *patC* (yellow), heat shock protein *htpX2* (green), and hypothetical proteins in gray. The black color indicates the insertion sequence in the DSM 20072 strain. (**B**). Representation of insertion sequences upstream of the gene *patC* in the DSM 20072 strain are represented. Black arrows indicate reversed repetitions, while white arrows indicate direct repetitions. The nucleotides in the sequence indicate the direct repeat where inserts of the IS110 insertion sequence are found.

To characterize the genomic *locus* of CEPs in *L. delbrueckii*, a syntenic analysis was performed. An overview of the genomic context of CEPs is presented in Figure 4, with more detailed information available in Supplementary Figures S5–S7. Synteny is highly conserved among all 14 strains of *L. delbrueckii* subsp. *bulgaricus* subspecies (Supplementary Figure S5). The *prt* gene is consistently located downstream of the acetyltransferase and aspartate ammonium lyase genes (*LBLM1_05370* and *asnA,* respectively) and upstream of the *patC* (cystathionine beta-lyase) and *htpx2* (heat shock protein) genes (Figure 4, Supplementary Figures S5–S7).

The *L. delbrueckii* subsp. *lactis* subspecies showed greater variability downstream of the *prt* gene, including the presence of insertion sequences, deletions, and rearrangements (Figure 4, Supplementary Figure S7). In *L. delbrueckii* subsp. *lactis* DSM 20072, an insertion sequence was found between the *patC* and *htpX2* genes. This insertion sequence contains imperfect reverse repeats of 26 bp flanked by direct repeats rich in A + T of 8 bp, and encodes a putative transposase consisting of 455 amino acids (Figure 4). Both the repeats and peptide sequence perfectly match what was previously reported by Ravin and Alatossava [19] for ISLdl1, exhibiting 100% identity in the amino acid sequence. Additionally, a second insertion sequence was located at the 5’ end of this sequence, showing 98% identity with the sequence described for IS110 by Bruton and Chater [20].

#### 2.2.3. Analysis of Promoter Regions and Putative Regulators

As mentioned before, CEPs exhibit a high degree of identity among them. However, there are significant variations in proteolytic activity and the strength of repression when strains are grown in the presence of peptides [4,7]. For instance, despite having 99% identity in their amino acid sequences, the CRL 581 strain shows ten-fold higher activity compared to the DSM 20072 strain. These two proteinases differ by only four amino acids out of a total of 1924 residues. These substitutions do not affect the active site environment of the enzyme (amino acids 1 to 497). Specifically, the substitutions from the strains CRL 581 to DSM 20072 are D606 to E606, T610 to A610, G638 to V638, and N658 to D658. Structural prediction analyses [18] indicate that these amino acid substitutions do not impact the three-dimensional structure of the proteinase. Therefore, an *in silico* analysis was performed on the promoter region of the *prt* genes. Furthermore, the presence of transcriptional regulators associated with the lactobacilli proteolytic system (BCAAR, PrcR, and YebC), along with their consensus sequences in the *prt* gene promoter region, was also examined. These *in silico* analyses of the proteolytic system of *L. delbrueckii* were primarily focused on CEPs, which serve as the key enzymes in the system and act as the primary drivers of proteolytic activity.

**Figure 5:**
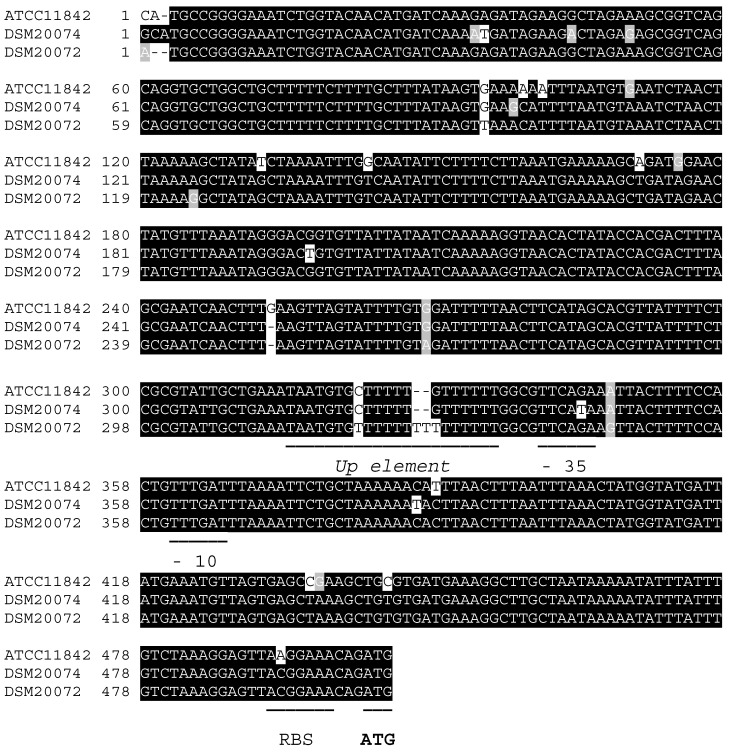
Comparative analysis of putative promoter sequences of CEPs from *L. delbrueckii* subspecies. Alignment of putative promoter sequences of CEPs from *L. delbrueckii* subsp. *bulgaricus* ATCC 11842, *L. delbrueckii* subsp. *delbrueckii* DSM 20074, and *L. delbrueckii* subsp. *lactis* DSM 20072. The figure depicts the 500 bp sequence upstream of the ATG start codon, indicating the UP element, the -0 element, the -35 element, and the ribosome binding site.

**Figure 6:**
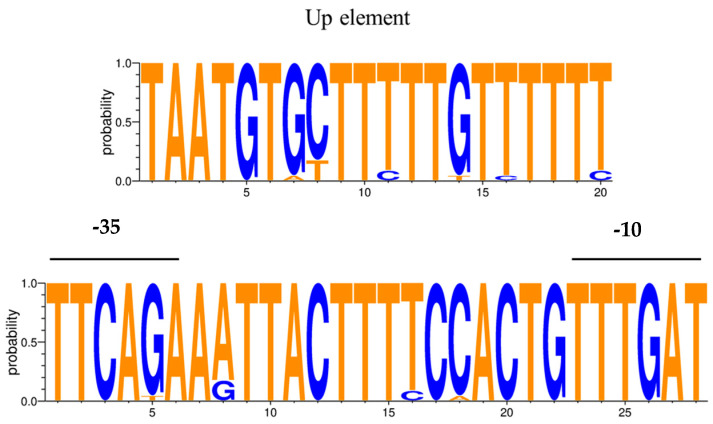
Sequence logo representation of the putative promoter consensus for the *prt* gene in *Lactobacillus delbrueckii* strains. Nucleotides that differ from those in the promoter described for the NCDO1489 strain are shown in blue.

The core promoter showed a high degree of conservation among the *L. delbrueckii* strains analyzed (Table 2, Figure 5). Only the promoter region associated with the proteinase of *L. delbrueckii* subsp. *delbrueckii* ATCC 9649 exhibited the substitution of guanine with thymidine at the -35 element. With the exception of *L. delbrueckii* subsp*. bulgaricus* MN-BM-F01 and *L. delbrueckii* subsp. *bulgaricus* VHProbi R03, which possess an additional nucleotide between the UP element and the -35 element, the extended promoter region remained conserved in all the examined strains of *L. delbrueckii* subsp. *bulgaricus*. In contrast, the UP element was less conserved in *L. delbrueckii* subsp. *lactis* strains, with small variations in 1–2 nucleotides. Based on these findings, a consensus sequence for the *prt* promoter was established (Figure 6).

A pangenomic analysis to identify potential regulators, including the *Lactobacillus helveticus* BCAA-responsive transcriptional regulator (BCAAR), the *Lactobacillus delbrueckii* DNA-binding protein YebC, and the *Lactobacillus casei* OmpR family response regulator, PrcR, was performed. Remarkably, BCAAR (OG0000219), YebC (OG0000477), and PrcR (OG0000151) were identified in all 27 *L. delbrueckii* strains examined. However, it is noteworthy that the consensus sequences described for BCAAR and PrcR were not identified in the promoter region of the analyzed *prt* genes. On the other hand, studies investigating the consensus binding region of YebC have not been conducted yet. This finding highlights the need for further investigation in this specific area, offering new opportunities for future research and exploration.

## 3. Discussion

Phylogenetic analysis using ANIm and MLSA revealed a clear distinction between *L. delbrueckii* subsp. *bulgaricus* and *L. delbrueckii* subsp. *lactis*. Surprisingly, the ND02 strain, initially identified as *L. delbrueckii* subsp. *bulgaricus* based on 16S RNA analysis and sugar assimilation profiles, was found to be closely related to the *L. delbrueckii* subsp. *lactis* CIDCA133 strain. Moreover, the ND02 strain has a considerably larger genome size (2.13 Mb) compared to the reported genome size of the *L. delbrueckii* subsp. *bulgaricus* subspecies (1.82–1.89 Mb), aligning more closely with the genome size of the *L. delbrueckii* subsp. *lactis* subspecies (2.00–2.26 Mb).

Liu et al. (2010) [11] conducted comparative studies on the proteolytic system of 22 LAB strains, which revealed that CEPs were present in only a few strains. In contrast, various peptidases genes, such as *pepC, pepN, pepM, pepX, pepQ*, *pepO*, and *pepV*, were found in all LAB genomes. Although comparative genomics approaches can distinguish different subgroups of peptidases, differences in enzyme specificity between these subgroups remain unclear.

The advent of advanced high-throughput sequencing techniques has led to a vast abundance of sequenced bacterial genomes becoming easily accessible. Therefore, this study aimed to analyze the genomic diversity of the proteolytic system, focusing specifically on the *L. delbrueckii* species. Through the analysis of 26 *L. delbrueckii* strains, a substantial repertoire of peptidase enzymes similar to those found in *L. delbrueckii* subsp. *lactis* CRL 581 were identified [7]. On the other hand, all strains featured a single *prt* gene encoding a putative CEP. This enzyme is of great significance in the proteolytic system as it plays a critical role in the initial step of protein hydrolysis. PrtB was found to be specific to *L. delbrueckii* subsp. *bulgaricus*, while PrtL was represented by strains of *L. delbrueckii* subsp. *delbrueckii* and *L. delbrueckii* subsp. *lactis*. 

The activity and specificity of a CEP is influenced not only by its amino acid sequence but also by its three-dimensional structure. In CEP alignments, the analyses revealed a high degree of identity among the CEP primary sequences, indicating a significant level of similarity in their amino acid compositions. Moreover, the predicted three-dimensional structures of these proteins exhibit a remarkable degree of conservation, suggesting that the overall folding and arrangement of critical protein domains are highly preserved. However, despite the high sequence identity and shared conformation among *L. delbrueckii* proteinases, their activity levels vary among different strains. This indicates that additional factors, beyond sequence and structure, contribute to the observed variation in enzyme activity. These differences may be influenced by various mechanisms, such as post-translational modifications, the regulation of gene expression, or the presence of specific regulatory elements within the proteolytic system. Therefore, a comparative analysis of putative promoters and regulatory sequences was performed to investigate their potential roles in modulating enzyme activity. 

In bacterial transcription, the process begins with the recognition of specific consensus sequences known as promoters, which are located upstream of the transcription start site (TSS). The core promoter, consisting of the -10 and -35 elements, is essential for optimal transcription initiation, with their optimal positioning relative to the TSS [21]. To enhance transcription, a third element known as the UP element, a sequence rich in A + T located between positions −40 and −60, is often present [22]. Gilbert et al. [23] identified these three elements in the promoter region of the *prtB* gene in *L. delbrueckii* subsp. *bulgaricus* NCDO1489. In our study, the putative promoter of *prt* was found to be conserved in the majority of the evaluated strains, and the -10 and -35 elements were separated by a distance of 16 bp according to Gilbert et al. [23]. In addition, the first 500 bp upstream of the initiation codon ATG were highly conserved in all three subspecies. This suggests that factors beyond differences in amino acid and promoter sequences contribute to the activity differences.

Microorganisms have evolved a regulatory mechanism to control the proteolytic system in response to changes in nitrogen availability, ensuring a balanced nitrogen metabolism within the cell. In *Lactococcus lactis*, which is one of the most extensively studied Gram-positive microorganisms after *Bacillus*, the expression of proteolytic enzymes is repressed through nitrogen catabolite repression when readily assimilable nitrogenous compounds are present [7,24]. This repression is mediated by CodY, which undergoes a conformational change upon binding to isoleucine, leucine, and valine, allowing it to bind to the operator on DNA and block the transcription of proteolytic enzyme synthesis genes.

The regulatory mechanisms of the proteolytic system in the *Lactobacillaceae* family are not fully understood. CodY homologues have not been identified in their genomes. In *L. helveticus* CM4, the BCAAR regulator binds to the 5′-AAAAANNCTWTTATT-3′ sequence within the promoter region of several genes involved in protein degradation and transport, including the *pepT2, pepCE, pepO, pepO2*, and *dppD* genes [25]. This binding occurs in the presence of branched-chain amino acids, leading to the repression of their transcription [25]. On the other hand, Alcantara et al. [26] identified a response regulator, PrcR, which regulates the expression of genes involved in amino acid biosynthesis, transport, intracellular peptidases, and proteinase in *Lacticaseibacillus paracasei* BL23. Although the exact DNA binding sequence for PrcR has not been determined, an AAAA motif may be involved in this regulation. Previously, we identified a putative transcriptional regulator belonging to the YebC family in *L. delbrueckii* subsp. *lactis* CRL 581 [7]. This regulator was found to bind to the promoter region of the *prtL* gene. Although the consensus sequence with which YebC matches has not yet been defined, its overexpression was observed in the presence of Casitone, a peptide-rich nitrogenous source [7]. In this *in silico* analysis of the 27 strains of *L. delbrueckii*, the presence of the three transcriptional regulators (YebC, BCAAR, and PrcR) was conserved. However, the consensus sequence of BCAAR or PrcR in the surroundings of the *prt* promoter was not identified. Therefore, further experiments are necessary to gain a comprehensive understanding of proteolytic activity regulation. Despite these limitations, this research represents the first comparative genomic analysis of the proteolytic system in *L. delbrueckii* strains.

## 4. Materials and Methods

### 4.1. Genome Selection

Genomic sequence data for *L. delbrueckii* strains were obtained from the National Center for Biotechnology Information (NCBI) database [27]. A total of 26 closed genomes from *Lactobacillus delbrueckii* subsp. *delbrueckii*, *L. delbrueckii* subsp. *bulgaricus*, and *L. delbrueckii* subsp. *lactis* (on April 2023) were downloaded and used for this study. The genome of the type strain *L. delbrueckii* subsp. *indicus* JCM 15610 was included as an outgroup. Additionally, the genome of the highly proteolytic strain *L. delbrueckii* subsp. *lactis* CRL 581 was specifically chosen as a study model. The genome annotations of the different strains are summarized in Table 3.

### 4.2. Average Nucleotide Identity (ANI) Analysis

DNA–DNA hybridization (DDH) is traditionally a widely used genomic taxonomic method. However, with the advancement of sequencing technologies and the reduction in sequencing costs, a new approach has emerged, which involves the massive sequencing of strains followed by the analysis and comparison of complete genomes or a large number of markers using bioinformatics tools [27,28]. One commonly used parameter in this approach is based on BLAST (ANIb). The calculation of ANI involves the comparison of base pairs in the genomes by identifying shared orthologous proteins or by dividing the genome into fragments of 1020 nucleotides [15]. Since the establishment of DDH, more advanced algorithms have emerged, such as MUMmer (ANIm) software, which creates and searches for data structures called suffix trees. These suffix trees can rapidly create sequence alignments containing millions of nucleotides [15]. ANIm provides more robust results when the pair of genomes compared share a high degree of similarity (ANI > 90%) [15]. Taking this into account, ANIm was used as a parameter for our comparative genomic analysis. ANIm calculation of the 28 strains (Table 3) was carried out using the JspeciesWS online server [28]. From these data, an ANI heatmap was constructed using the heatmap.2 function of the gplots R package (version 3.1.3).

### 4.3. Multilocus Sequence Analysis (MLSA) and Pangenomic Analysis

Genomes were re-annotated using PGAP v4.8 [29] in the stand-alone configuration. Amino acid sequences encoded in *L. delbrueckii* genomes were compared for ortholog group inference using OrthoFinder v2.5.4. [30]. A phylogenetic tree was constructed from the following housekeeping gene sequences: *clpX*, *dnaA*, *hsp60*, *murE*, *pheS*, and *pyrG*. Housekeeping genes for MLSA were selected based on previous work [31]. Using a multiple alignment program (MUSCLE) [32], the sequences were aligned and the tree was built from the maximum likelihood estimation statistical test [33], available in MEGAX [34]. 

### 4.4. In Silico Analysis of Promoter Region and Predicted Amino Acid Sequence of prt Genes

Genomes downloaded from Genbank were uploaded to the RAST server [35]. CEP sequences were obtained from the RAST server by performing BlastP alignment with the proteinase PrtL sequence of CRL 581. Protein sequence alignments were carried out using the T-coffee online tool [36] and colored using the Boxshade v3.2 tool. The aligned sequences were later used to build a tree using MEGA X version 10.1 software [34]. A phylogenetic tree was inferred using the neighbor-joining method, with the p distance as a criterion and the PrtP sequence of *Lactococcus Lactis* SK11 as an outgroup. The promoters of *prt* genes were identified via homology with the *prtB* promoter sequence from *L. delbrueckii* subsp*. bulgaricus* NCDO 1489 described by Gilbert et al. [23], considering the 500 pb sequence upstream of the ATG of each *prt*. Sequence logos were generated using the web-based application WebLogo 3.7.11 [37]. The synteny plots around the *prt* genes of *L. delbrueckii* strains were created using Easyfig v2.2.5 software [38] and the BLASTn algorithm.

### 4.5. Protein Modeling and Visualization

The Phyre2 web portal was utilized for protein modeling, prediction, and analysis [39]. For the visualization and selection of molecular chains, the EzMol software was employed [40]. 

## 5. Conclusions

This comprehensive genomic analysis of the proteolytic system in *L. delbrueckii* strains revealed the presence of various peptidase genes, including *pepC, pepD, pepF, pepI, pepM, pepN, pepO, pepQ, pepR, pepT, pepV,* and pepX, in the core-genome. The *prt* gene encoding CEPs was present in all *L. delbrueckii* strains, with two subtypes (*prtB* in *L. delbrueckii* subsp. *bulgaricus* and *prtL* in *L. delbrueckii* subsp. *lactis* and *L. delbrueckii* subsp. *delbrueckii*). The structural analysis of the CEPs confirmed the presence of conserved catalytic triads, and the predicted structural impact of the amino acid substitution at position 90 (S to C) did not alter the conformational structure of the enzyme. Synteny analysis demonstrated high conservation of the genomic context of the *prt* gene among *L. delbrueckii* subsp. *bulgaricus* strains, while *L. delbrueckii* subsp. *lactis* strains displayed greater variability, including the presence of insertion sequences, deletions, and rearrangements. *In silico* analysis of the *prt* gene promoter region revealed a high degree of conservation in the core promoter and extended promoter regions among the *L. delbrueckii* strains. Consensus sequences for the *prt* promoter were established. These findings contribute to our understanding of the proteolytic activity of *L. delbrueckii* and its potential applications in the dairy industry. Further research in this field will expand our knowledge and practical utilization of these findings.

## Figures and Tables

**Table 1:** Pangenome analysis of principal peptidases of *L. delbrueckii* strains.

Peptidase	Orthogroup	MEROPS Family	Gene (Locus) in CRL 581	*Pangenome analysis*
Aminopeptidases		4		
Aminopeptidase C	OG0000892	C1	pepC (G134_RS08320)	Core-genome
Lysine aminopeptidase	OG0000959	M1	pepN (G134_RS03335)	Core-genome
Glutamine aminopeptidase	OG0000394	M42	pepA (G134_RS00305)	Softcore-genome
Methionine aminopeptidase	OG0000412	M24A	pepM (G134_RS00100)	Core-genome
Endopeptidases				
Oligopeptidase F	OG0000936	M3	pepF (G134_RS03600)	Core-genome
Oligopeptidase O	OG0000119	M13	pepO (G134_RS01630)	Core-genome
Peptidase G	OG0001037	C1	pepG/E (G134_RS01440)	Softcore-genome
Dipeptidases				
Dipeptidase A	OG0000081	C69	pepD (G134_RS03315)	Core-genome
Dipeptidase V	OG0000164	M20A	pepV (G134_RS08405)	Core-genome
Tripeptidases				
Tripeptidase T	OG0000320	M20B	pepT (G134_RS01035)	Core-genome
Prolyl peptidases				
Aminopeptidase P	OG0001207	M24B	pepP (G134_RS05655)	Softcore-genome
Prolidase	OG0000832	M24B	pepQ (G134_RS07685)	Core-genome
X-Prolyl-dipeptidyl aminopeptidase	OG0000775	S15	pepX (G134_RS05545)	Core-genome
Proline dipeptidase	OG0000913	S33	pepR (G134_RS08875)	Core-genome
Proline aminopeptidase	OG0001790	S33	pepL (G134_RS06955)	Shell-genome
Proline aminopeptidase	OG0000907	S33	pepI (G134_RS08815)	Core-genome

**Table 2:** Putative promoter sequences of the *prt* gene for different strains of *L. delbrueckii*.

Strain	UP Element	b ^1^	Element-35	a ^2^	Element-10
*L. delbrueckii* subsp. *bulgaricus* NCDO1489	TAATGTGCTTTTTGTTTTTT	4	TTCAGA	16	TTTGAT
*L. delbrueckii* subsp. *bulgaricus* 2038	TAATGTGCTTTTTGTTTTTT	4	TTCAGA	16	TTTGAT
*L. delbrueckii* subsp. *bulgaricus* ACA-DC 87	TAATGTGCTTTTTGTTTTTT	4	TTCAGA	16	TTTGAT
*L. delbrueckii* subsp. *bulgaricus* ATCC-BAA365	TAATGTGCTTTTTGTTTTTT	4	TTCAGA	16	TTTGAT
*L. delbrueckii* subsp. *bulgaricus* ATCC 11842	TAATGTGCTTTTTGTTTTTT	4	TTCAGA	16	TTTGAT
*L. delbrueckii* subsp. *bulgaricus* DSM 20080	TAATGTGCTTTTTGTTTTTT	4	TTCAGA	16	TTTGAT
*L. delbrueckii* subsp. *bulgaricus* KLDS 1.0207	TAATGTGCTTTTTGTTTTTT	4	TTCAGA	16	TTTGAT
*L. delbrueckii* subsp. *bulgaricus* KLDS 1.1011	TAATGTGCTTTTTGTTTTTT	4	TTCAGA	16	TTTGAT
*L. delbrueckii* subsp. *bulgaricus* LBA-40	TAATGTGCTTTTTGTTTTTT	4	TTCAGA	16	TTTGAT
*L. delbrueckii* subsp. *bulgaricus* LDB-CI	TAATGTGCTTTTTGTTTTTT	4	TTCAGA	16	TTTGAT
*L. delbrueckii* subsp. *bulgaricus* LJJ	TAATGTGCTTTTTGTTTTTT	4	TTCAGA	16	TTTGAT
*L. delbrueckii* subsp. *bulgaricus* L99	TAATGTGCTTTTTGTTTTTT	4	TTCAGA	16	TTTGAT
*L. delbrueckii* subsp. *bulgaricus* MN-BM-F01	TAATGTGCTTTTTGTTTTTT	5	TTCAGA	16	TTTGAT
*L. delbrueckii* subsp. *bulgaricus* ND04	TAATGTGCTTTTTGTTTTTT	4	TTCAGA	16	TTTGAT
*L. delbrueckii* subsp. *bulgaricus* VHProbi R03	TAATGTGCTTTTTGTTTTTT	5	TTCAGA	16	TTTGAT
*L. delbrueckii* subsp. *delbrueckii* ATCC 9649	TAATGTGCTTTTTGTTTTTT	4	TTCA**T**A	16	TTTGAT
*L. delbrueckii* subsp. *delbrueckii* TUA4408L	TAATGT**A**CTTTTTGT**C**TTTT	4	TTCAGA	16	TTTGAT
*L. delbrueckii* subsp. *lactis* CRL 581	TAATGTG**T**TTTTTGTTTTTT	4	TTCAGA	16	TTTGAT
*L. delbrueckii* subsp. *lactis* CIDCA133	TAATGTGCTT**C**TTGTTTTT**C**	4	TTCAGA	16	TTTGAT
*L. delbrueckii* subsp. *lactis* DSM 20072	TAATGTG**T**TTTTT**T**TTTTTT	6	TTCAGA	16	TTTGAT
*L. delbrueckii* subsp. *lactis* MAG_rmk202_ldel	TAATGTG**T**TTTTTGTTTTTT	4	TTCAGA	16	TTTGAT
*L. delbrueckii* subsp. *lactis* NWC-1-2	TAATGTG**T**TTTTTGTTTTTT	4	TTCAGA	16	TTTGAT
*L. delbrueckii* ND02	TAATGTGCTT**C**TTGTTTTT**C**	4	TTCAGA	16	TTTGAT

Nucleotides that differ from those of the promoter described for the NCDO1489 strain are shown in blue. ^1^ distance between the UP element and the -35 element.; ^2^ distance between the -35 element and -10 element.

**Table 3:** Genomes of *Lactobacillus delbrueckii* strains used in this work.

Strain	Species	G + C%	Access	Chromosome	Plasmid
2038	*L. delbrueckii* subsp. *bulgaricus*	49.7	GCA_000191165.1	CP000156.1	
ACA-DC-87	*L. delbrueckii* subsp. *bulgaricus*	49.8	GCA_900196735.1	NZ_LT899687.1/LT899687.1	
ATCC 11842 (DSM 20081)	*L. delbrueckii* subsp. *bulgaricus*	49.7	GCA_000056065.1	NC_008054.1/CR954253.1	
ATCC BAA-365	*L. delbrueckii* subsp. *bulgaricus*	49.7	GCA_000014405.1	NC_008529.1/CP000412.1	
DSM 20080	*L. delbrueckii* subsp. *bulgaricus*	49.8	GCA_001953135.1	NZ_CP019120.1/CP019120.1	
KLDS 1.0207	*L. delbrueckii* subsp. *bulgaricus*	49.8	GCA_003597655.1	NZ_CP032451.1/CP032451.1	
KLDS 1.1011	*L. delbrueckii* subsp. *bulgaricus*	49.8	GCA_006704185.1	NZ_CP041280.1/CP041280.1	
LBA-40	*L. delbrueckii* subsp. *bulgaricus*	49.9	GCA_024665995.1	NZ_CP102529.1/CP102529.1	
LDB-C1	*L. delbrueckii* subsp. *bulgaricus*	50.0	GCA_023205755.1	NZ_CP050929.1/CP050929.1	
L99	*L. delbrueckii* subsp. *bulgaricus*	49.7	GCA_003351805.1	NZ_CP017235.1/CP017235.1	
LJJ	*L. delbrueckii* subsp. *bulgaricus*	49.5	GCA_011044195.1	NZ_CP049052.1/CP049052.1	
MN-BM-F01	*L. delbrueckii* subsp. *bulgaricus*	49.7	GCA_001469775.1	NZ_CP013610.1/CP013610.1	
ND02	L. *delbrueckii* subsp. *bulgaricus*	49.6	GCA_000182835.1	NC_014727.1/CP002341.1	NC_014728.1/CP002342.1
ND04	*L. delbrueckii* subsp. *bulgaricus*	49.6	GCA_002000885.1	NZ_CP016393.1/CP016393.1	
VHProbi R03	*L. delbrueckii* subsp. *bulgaricus*	49.7	GCA_023204995.1	NZ_CP096210.1/CP096210.1	
DSM 20074 (ATCC 9649)	*L. delbrueckii* subsp. *delbrueckii*	49.6	GCA_001908495.1	NZ_CP018615.1/CP018615.1	
KCTC 13731	*L. delbrueckii* subsp. *delbrueckii*	50.0	GCA_001888945.1	NZ_CP018216.1/CP018216.1	
NBRC 3202	*L. delbrueckii* subsp. *delbrueckii*	50.1	GCA_006740305.1	NZ_AP019750.1/AP019750.1	
TUA4408L	*L. delbrueckii* subsp. *delbrueckii*	49.9	GCA_002142575.1	NZ_CP021136.1/CP021136.1	
JCM 15610	*L. delbrueckii* subsp. *indicus*	49.4	GCA_001908415.1	NZ_CP018614.1/CP018614.1	pLD01: NZ_CP018612.1/CP018612.1pLD02: NZ_CP018613.1/CP018613.1
CIDCA133	*L. delbrueckii* subsp. *lactis*	49.6	GCA_021091115.1	NZ_CP065513.1/CP065513.1	NZ_CP065513.1/CP065513.1
CRL 581	*L. delbrueckii* subsp. *lactis*	49.6	GCA_000409675.1		
DSM 20072	*L. delbrueckii* subsp. *lactis*	49.1	49.1	NZ_CP018215.1/CP018215.1	
KCCM 34717	*L. delbrueckii* subsp. *lactis*	49.1	GCA_001888905.1	NZ_CP018215.1/CP018215.1	
KCTC 3034	*L. delbrueckii* subsp. *lactis*	49.0	GCA_002285775.1	NZ_CP023139.1/CP023139.1	
KCTC 3035	*L. delbrueckii* subsp. *lactis*	50.0	GCA_001888985.1	NZ_CP018156.1/CP018156.1	
MAG_RMK202_LDEL	*L. delbrueckii* subsp. *lactis*	49.0	GCA_017584225.1	NZ_CP046131.1/CP046131.1	p202_01: NZ_CP046132.1/CP046132.1p202_02: NZ_CP046133.1/CP046133.1
NWC_1_2	*L. delbrueckii* subsp. *lactis*	48.6	GCA_003814285.1	CP029250.1	p1 CP029251.1

## Data Availability

The data are contained within the article.

## Supplementary Materials

The following supporting information can be downloaded at: https://www.mdpi.com/article/10.3390/ijms241411309/s1.
