# Supplementary material for: In Silico Comparative Genomic Analysis Revealed a Highly Conserved Proteolytic System in Lactobacillus delbrueckii
Source: Int J Mol Sci. 2023 Jul 11;24(14):11309. doi: 10.3390/ijms241411309 (PMC10379286; doi:10.3390/ijms241411309)
Supplement: Supplementary file 1 [file ijms-24-11309-s001.zip › ijms-2456998-supplementary.pdf]

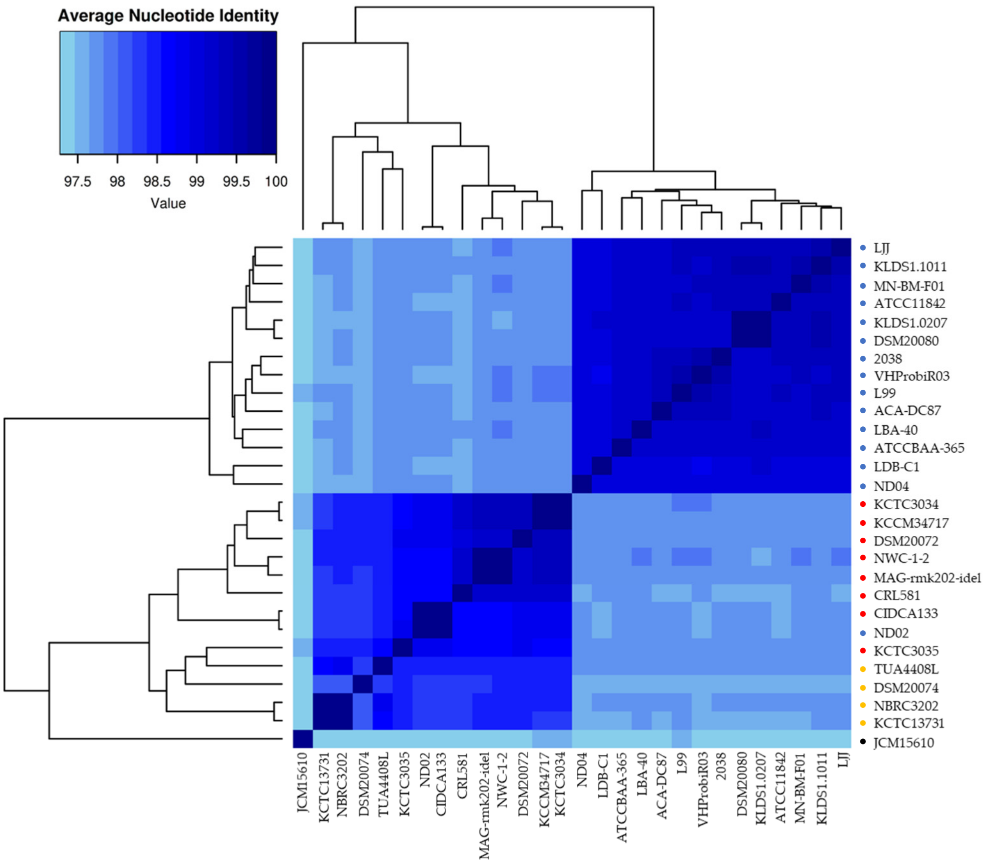

Supplementary Figure S1. Heat map of average nucleotide identity (ANIm) values for *Lactobacillus delbrueckii* strains. The subspecies to which they belong are indicated with colored circles: *L. delbrueckii* subsp. *bulgaricus* in blue, *L. delbrueckii* subsp. *lactis* in red and *L. delbrueckii* subsp. *delbrueckii* in green. ANIm values were calculated using the JSpeciesWS online server (Richter et al., 2009 [15]). *L. delbrueckii* subsp. *indicus* JCM15610 (black circle) was used as an outgroup.

[15] Richter, M.; Rossello-Mora, R. Shifting the genomic gold standard for the prokaryotic species definition. *Proc. Natl. Acad. Sci. USA* **2009**, *106*, 19126–19131.

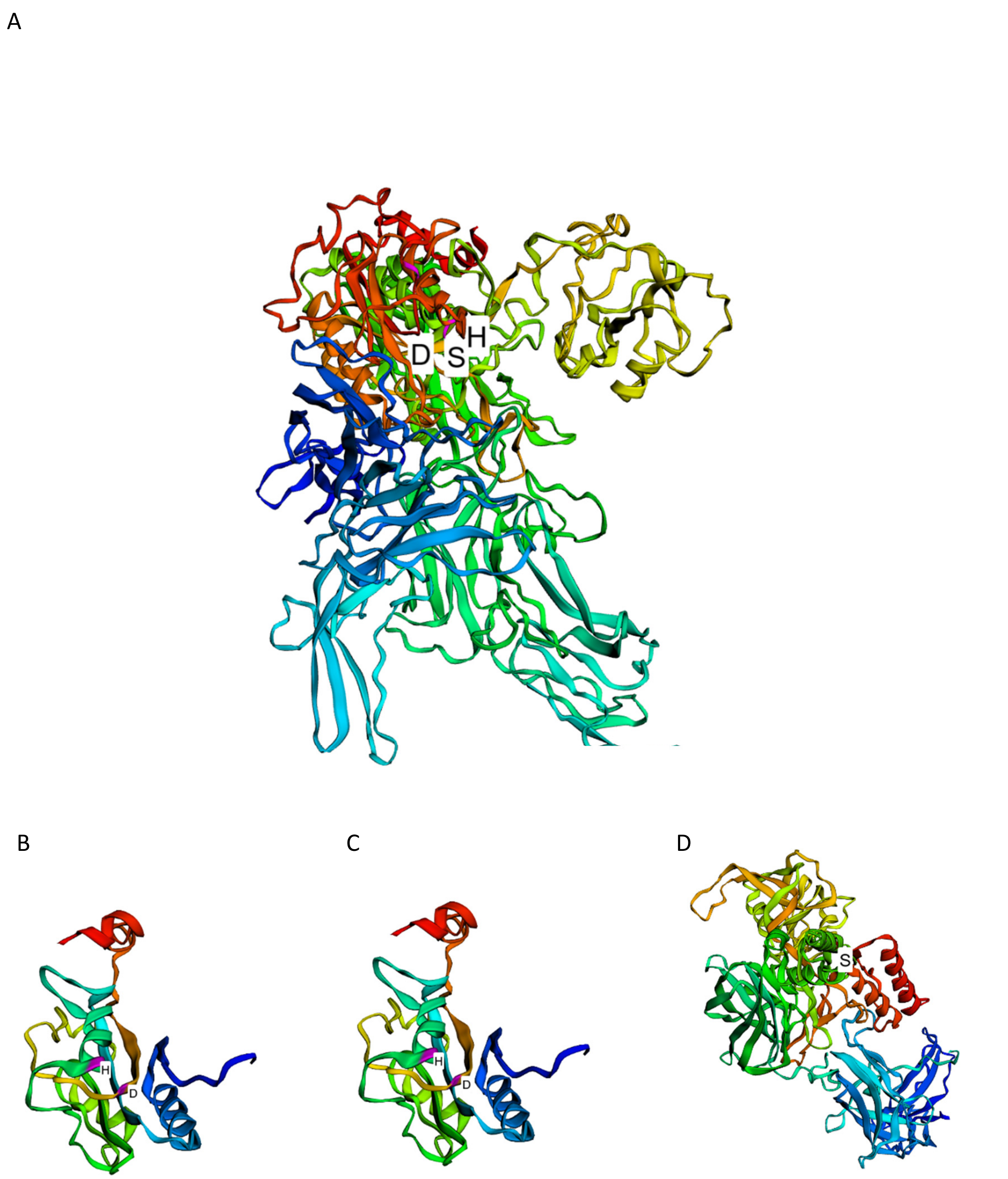

Supplementary Figure S2. Structure prediction model of the proteinase from *L. delbrueckii* subsp. *lactis* CRL 581 (A), *L. delbrueckii* subsp. *delbrueckii* KCTC 13731 (B), *L. delbrueckii* subsp. *delbrueckii* NBRC 3202 (C) and *L. delbrueckii* subsp. *lactis* KCTC 3035 (D). A cartoon representation of a protein monomer shown in rainbow colours from red at the N-terminus, to blue at the C-terminus. The active site of the enzyme contains the catalytic triad of amino acids: D, H, and S.

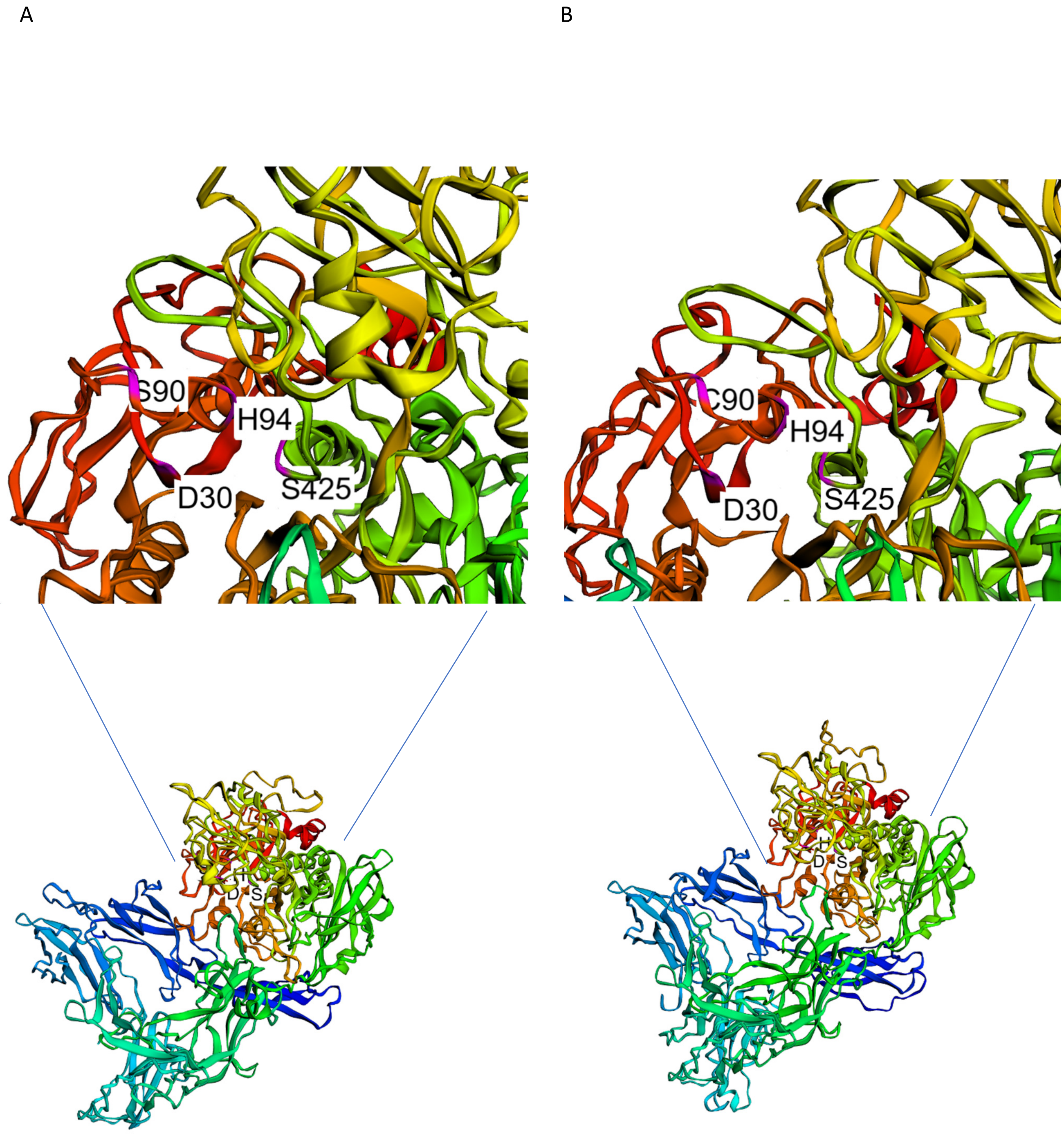

Supplementary Figure S3. Structure prediction model of the proteinase PrtL from *L. delbrueckii* subsp. *lactis* CRL 581 (A) and PrtB from *L. delbrueckii* subsp. *bulgaricus* ATCC 11842 (B). A cartoon representation of a protein monomer shown in rainbow colours from red at the N-terminus, to blue at the C-terminus. The active site of the enzyme contains the catalytic triad of amino acids: D, H, and S. The substitution S90 to C90 from PrtL to PrtB is also represented.

```
2038               1 MQKKKSARYLNKAAELGA-------------A----LLLSASPLAGTFQSAAFVQAASQE  43
ACA-DC87           1 MQKKKSARHLNKVAELAA-------------A----LLLSASPLAGTFQSAAFVQAASQE  43
ATCC11842          1 MQKKKSARHLNKVAELAA-------------A----LLLSASPLAGTFQSAAFVQAASQE  43
ATCC-BAA-365       1 MQKKKSARHLSKVAELAA-------------A----LLLSASPLAGTFQSAAFVQAASQE  43
DSM20080           1 MQKKKSARHLNKVAELAA-------------A----LLLSASPLAGTFQSAAFVQAASQE  43
KLDS1.0207         1 MQKKKSARHLNKVAELAA-------------A----LLLSASPLAGTFQSAAFVQAASQE  43
KLDS1.1011         1 MQKKKSARHLNKVAELAA-------------A----LLLSASPLAGTFQPAAFVQAASQE  43
LBA-40             1 MQKKKSARHLSKVAELAA-------------A----LLLSASPLAGTFQSAAFVQAASQE  43
LDB-C1             1 MQKKKSARHLNKVAELAA-------------A----LLLSASPLAGTFQSAAFVQAASQE  43
L99                1 MQKKKSARHLNKVAELAA-------------A----LLLSASPLAGTFQSAAFVQAASQE  43
LJJ                1 MQKKKSARHLNKVAELAA-------------A----LLLSASPLAGTFQSAAFVQAASQE  43
MN-BM-F01          1 MQKKKSARHLNKVAELAA-------------A----LLLSASPLAGTFQSAAFVQAASQE  43
ND04               1 MQKKKSARHLNKVAELAA-------------A----LLLSASPLAGTFQSAAFVQAASQE  43
VHProbi-R03        1 MQKKKSARHLNKVAELAA-------------A----LLLSASPLAGTFQSAAFVQAASQE  43
ATCC9649           1 MQKKKSARNLNKVAELAA-------------A----LLLSASPLAGTFQSAAFVQAASQE  43
TUA4408L           1 MQKKKSARHLNKVAELAA-------------A----LLLSASPLAGTFQSATFVQAASQE  43
CIDCA133           1 MQKKKSARHLNKVAELAA-------------A----LLLSASPLAGTFQSAAFVQAASQE  43
CRL581             1 MQKKKSARHLNKVAELAA-------------A----LLLSASPLAGTFQSAAFVQAASQE  43
DSM20072           1 MQKKKSARHLNKVAELAA-------------A----LLLSASPLAGTFQSAAFVQAASQE  43
KCCM34717          1 MQKKKSARHLNKVAELAA-------------A----LLLSASPLAGTFQSAAFVQAASQE  43
KCTC3034           1 MQKKKSARHLNKVAELAA-------------A----LLLSASPLAGTFQSAAFVQAASQE  43
MAG_rmk202_ldel    1 MQKKKSARHLNKVAELAA-------------A----LLLSASPLAGTFQSATFVQAASQE  43
NWC_1_2            1 MQKKKSARHLNKVAELAA-------------A----LLLSASPLAGTFQSATFVQAASQE  43
ND02               1 MQKKKSARHLNKVAELAA-------------A----LLLSASPLAGTFQSAAFVQAASQE  43
SK11               1 MQRKKKGLSILLAGTVALGALAVLPVGEIQAKAAISQQTKGSSLANTVTAATAKQAATDT  60

2038              44 KVSPRSASRAALTKYLQQEQRYNAKKSYSKFQEAAKEQRQASGQAVSKKNESSVRVIVSL 103
ACA-DC87          44 KVSPRSASRAALTKYLQQEQRYNAKKSYSKFQEAAKEQRQASGQAVSKKNESSVRVIVSL 103
ATCC11842         44 TVSPRSASRAALTKYLQQEQRYNAKKSYSKFQEAAKEQRQASGQAVSKKNESSVRVIVSL 103
ATCC-BAA-365      44 TVSPRSASRAALTKYLQQEQRYNAKKSYSKFQEAAKEQRQASGQAVSKKNESSVRVIVSL 103
DSM20080          44 KVSPRSASRAALTKYLQQEQRYNAKKSYSKFQEAAKEQRQASGQAVSKKNESSVRVIVSL 103
KLDS1.0207        44 KVSPRSASRAALTKYLQQEQRYNAKKSYSKFQEAAKEQRQASGQAVSKKNESSVRVIVSL 103
KLDS1.1011        44 KVSPRSASRAALTKYLQQEQRYNAKKSYSKFQEAAKEQRQTSGQAVSKKNESSVRVIVSL 103
LBA-40            44 TVSPRSASRAALTKYLQQEQRYNAKKSYSKFQEAAKEQRQASGQAVSKKNESSVRVIVSL 103
LDB-C1            44 KVSPRSASRAALTKYLQQEQRYNAKKSYSKFQEAAKEQRQASGQAVSKKNESSVRVIVSL 103
L99               44 TVSPRSASRAALTKYLQQEQRYNAKKSYSKFQEAAKEQRQASGQAVSKKNESSVRVIVSL 103
LJJ               44 KVSPRSASRAALTKYLQQEQRYNAKKSYSKFQEAAKEQRQASGQAVSKKNESSVRVIVSL 103
MN-BM-F01         44 KVSPRSASRAALTKYLQQEQRYNAKKSYSKFQEAAKEQRQASGQAVSKKNESSVRVIVSL 103
ND04              44 KVSPRSASRAALTKYLQQEQRYNAKKSYSKFQEAAKEQRQASGQAVSKKNESSVRVIVSL 103
VHProbi-R03       44 KVSPRSASRAALTKYLQQEQRYNAKKSYSKFQEAAKEQRQASGQAVSKKNESSVRVIVSL 103
ATCC9649          44 TVSPRSASRVALTKYLQQEQRYNAKESYSKFQEAAKEQRQASGQAVSKKNESSVRVIVSL 103
TUA4408L          44 TVSPRSASRAALTKYLQQEQRYNAKESYSKFQEAAKEQRQASGQAVSKKNESSVRVIVSL 103
CIDCA133          44 TVSPRSASRAALTKYLQQEQRYNAKKSYSKFQEAAKEQRQASGQAVSKKNESSVRVIVSL 103
CRL581            44 TVSPRSASRAALTKYLQQEQRYNAKESYSKFQEAAKEQRQASGQAVSKKNESSVRVIVSL 103
DSM20072          44 TVSPRSASRAALTKYLQQEQRYNAKESYSKFQEAAKEQRQASGQAVSKKNESSVRVIVSL 103
KCCM34717         44 TVSPRSASRAALTKYLQQEQRYNAKESYSKFQEAAKEQRQASGQAVSKKNESSVRVIVSL 103
KCTC3034          44 TVSPRSASRAALTKYLQQEQRYNAKESYSKFQEAAKEQRQASGQAVSKKNESSVRVIVSL 103
MAG_rmk202_ldel   44 TVSPRSASRAALTKYLQQEQRYNAKKSYSKFQEAAKEQRQASGQAVSKKNESSVRVIVSL 103
NWC_1_2           44 TVSPRSASRAALTKYLQQEQRYNAKKSYSKFQEAAKEQRQASGQAVSKKNESSVRVIVSL 103
ND02              44 TVSPRSASRAALTKYLQQEQRYNAKKSYSKFQEAAKEQRQASGQAVSKKNESSVRVIVSL 103
SK11              61 TAATT--NQAIATQLAAKGIDYNK------LNK-------------VQQQDIYVDVIVQM  99
```

```
2038               104  NKSAAFDHTSKPTGSAASVKKIEQASDQVKDGQEKVIKQVEEITGNKVRRQFGYLVNAFS  163
ACA-DC87           104  NKSAAFDHTSKPTGSAASVKKIEQASDQVKDGQEKVIKQVEEITGNKVRRQFGYLVNAFS  163
ATCC11842          104  NKSAAFDHTSKPTGSAASVKKIEQASDQVKDGQEKVIKQVEEITGNKVRRQFGYLVNAFS  163
ATCC-BAA-365       104  NKSAAFDHTSKPTGSAASVKKIEQASDQVKDGQEKVIKQVEEITGNKVRRQFGYLVNAFS  163
DSM20080           104  NKSAAFDHTSKPTGSAASVKKIEQASDQVKDGQEKVIKQVEEITGNKVRRQFGYLVNAFS  163
KLDS1.0207         104  NKSAAFDHTSKPTGSAASVKKIEQASDQVKDGQEKVIKQVEEITGNKVRRQFGYLVNAFS  163
KLDS1.1011         104  NKSAAFDHTSKPTGSAASVKKIEQASDQVKDGQEKVIKQVEEITGNKVRRQFGYLVNAFS  163
LBA-40             104  NKSAAFDHTSKPTGSAASVKKIEQASDQVKDGQEKVIKQVEEITGNKVRRQFGYLVNAFS  163
LDB-C1             104  NKSAAFDHTSKPTGSAASVKKIEQASDQVKDGQEKVIKQVEEITGNKVRRQFGYLVNAFS  163
L99                104  NKSAAFDHTSKPTGSAASVKKIEQASDQVKDGQEKVIKQVEEITGNKVRRQFGYLVNAFS  163
LJJ                104  NKSAAFDHTSKPTGSAASVKKIEQASDQVKDGQEKVIKQVEEITGNKVRRQFGYLVNAFS  163
MN-BM-F01          104  NKSAAFDHTSKPTGSAASVKKIEQASDQVKDGQEKVIKKVEEITGNKVRRQFGYLVNAFS  163
ND04               104  NKSAAFDHTSKPTGSAASVKKIEQASDQVKDGQEKVIKQVEEITGNKVRRQFGYLVNAFS  163
VHProbi-R03        104  NKSAAFDHTSKPTGSAASVKKIEQASDQVKDGQEKVIKKVEEITGNKVRRQFGYLVNAFS  163
ATCC9649           104  NKSAAFDHTSKPTGSAASVKKIEQASDQVKDGQEKVIKQVEEITGNKVRRQFGYLVNAFS  163
TUA4408L           104  NKSAAFDHTSKPTGSAASVKKIEQASDQVKDGQEKVIKQVEEITGNKVRRQFGYLVNAFS  163
CIDCA133           104  NKSAAFDHTSKPTGSAASVKNIEQASDQVKDGQEKVIKQVEEITGNKVRRQFGYLVNAFS  163
CRL581             104  NKSAAFDHTSKPTGSAASVKKIEQASDQVKDGQEKVIKQVEEITGNKVRRQFGYLVNAFS  163
DSM20072           104  NKSAAFDHTSKPTGSAASVKKIEQASDQVKDGQEKVIKQVEEITGNKVRRQFGYLVNAFS  163
KCCM34717          104  NKSAAFDHTSKPTGSAASVKKIEQASDQVKDGQEKVIKQVEEITGNKVRRQFGYLVNAFS  163
KCTC3034           104  NKSAAFDHTSKPTGSAASVKKIEQASDQVKDGQEKVIKQVEEITGNKVRRQFGYLVNAFS  163
MAG_rmk202_ldel    104  NKSAAFDHTSKPTGSAASVKKIEQASDQVKDGQEKVIKQVEEITGNKVRRQFGYLVNAFS  163
NWC_1_2            104  NKSAAFDHTSKPTGSAASVKKIEQASDQVKDGQEKVIKQVEEITGNKVRRQFGYLVNAFS  163
ND02               104  NKSAAFDHTSKPTGSAASVKNIEQASDQVKDGQEKVIKQVEEITGNKVRRQFGYLVNAFS  163
SK11               100  SAAPASENGTLRTDY-SSTAEIQQETNKVIAAQASVKAAVEQVTQQTAGESYGYVVNGFS  158

2038               164  IDMDLDDIDKVKDLPQVKNVTPVKVYHPTDESADQMAQVQDVWQEQKLKGEGMVISIIDT  223
ACA-DC87           164  IDMDLDDIDKVKDLPQVKNVTPVKVYHPTDESADQMAQVQDVWQEQKLKGEGMVISIIDT  223
ATCC11842          164  IDMDLDDIDKVKDLPQVKNVTPVKVYHPTDESADQMAQVQDVWQEQKLKGEGMVISIIDT  223
ATCC-BAA-365       164  IDMDLDDIDKVKDLPQVKNVTPVKVYHPTDESADQMAQVQDVWQEQKLKGEGMVISIIDT  223
DSM20080           164  IDMDLDDIDKVKDLPQVKNVTPVKVYHPTDESADQMAQVQDVWQEQKLKGEGMVISIIDT  223
KLDS1.0207         164  IDMDLDDIDKVKDLPQVKNVTPVKVYHPTDESADQMAQVQDVWQEQKLKGEGMVISIIDT  223
KLDS1.1011         164  IDMDLDDIDKVKDLPQVKNVTPVKVYHPTDESADQMAQVQDVWQEQKLKGEGMVISIIDT  223
LBA-40             164  IDMDLDDIDKVKDLPQVKNVTPVKVYHPTDESADQMAQVQDVWQEQKLKGEGMVISIIDT  223
LDB-C1             164  IDMDLDDIDKVKDLPQVKNVTPVKVYHPTDESADQMAQVQDVWQEQKLKGEGMVISIIDT  223
L99                164  IDMDLDDIDKVKDLPQVKNVTPVKVYHPTDESADQMAQVQDVWQEQKLKGEGMVISIIDT  223
LJJ                164  IDMDLDDIDKVKDLPQVKNVTPVKVYHPTDESADQMAQVQDVWQEQKLKGEGMVISIIDT  223
MN-BM-F01          164  IDMDLDDIDKVKDLPQVKNVTPVKVYHPTDESADQMAQVQDVWQEQKLKGEGMVISIIDT  223
ND04               164  IDMDLDDIDKVKDLPQVKNVTPVKVYHPTDESADQMAQVQDVWQEQKLKGEGMVISIIDT  223
VHProbi-R03        164  IDMDLDDIDKVKDLPQVKNVTPVKVYHPTDESADQMAQVQDVWQEQKLKGEGMVISIIDT  223
ATCC9649           164  IDMDLDDIDKVKDLPQVKNVTPVKVYHPTDESADQMAQVQDVWQEQKLKGEGMVISIIDT  223
TUA4408L           164  IDMDLDDIDKVKDLPQVKNVTPVKVYHPTDESADQMAQVQDVWQEQKLKGEGMVISIIDT  223
CIDCA133           164  IDMDLDDIDKVKDLPQVKNVTPVKVYRPTDESADQMAQVQDVWQEQKLKGEGMVISIIDT  223
CRL581             164  IDMDLDDIDKVKDLPQVKNVTPVKVYHPTDESADQMAQVQDVWQEQKLKGEGMVISIIDT  223
DSM20072           164  IDMDLDDIDKVKDLPQVKNVTPVKVYHPTDESADQMAQVQDVWQEQKLKGEGMVISIIDT  223
KCCM34717          164  IDMDLDDIDKVKDLPQVKNVTPVKVYHPTDESADQIAQVQDVWQEQKLKGEGMVISIIDT  223
KCTC3034           164  IDMDLDDIDKVKDLPQVKNVTPVKVYHPTDESADQIAQVQDVWQEQKLKGEGMVISIIDT  223
MAG_rmk202_ldel    164  IDMDLDDIDKVKDLPQVKNVTPVKVYHPTDESADQMAQVQDVWQEQKLKGEGMVISIIDT  223
NWC_1_2            164  IDMDLDDIDKVKDLPQVKNVTPVKVYHPTDESADQMAQVQDVWQEQKLKGEGMVISIIDT  223
ND02               164  IDMDLDDIDKVKDLPQVKNVTPVKVYRPTDESADQMAQVQDVWQEQKLKGEGMVISIIDT  223
SK11               159  TKVRVVDIPKLKQIAGVKTVTLAKVYYPTDAKANSMANVQAVWSNYKYKGEGTVVSVIDS  218
```

```
2038                224 GIDSSHQDLKLDSGVSTALSKSEVESDKSKLGHGKYYTEKVPYGYNYADKNDQIVDNGCG 283
ACA-DC87            224 GIDSSHQDLKLDSGVSTALSKSEVESDKSKLGHGKYYTEKVPYGYNYADKNDQIVDNGCG 283
ATCC11842           224 GIDSSHQDLKLDSGVSTALSKSEVESDKSKLGHGKYYTEKVPYGYNYADKNDQIVDNGCG 283
ATCC-BAA-365        224 GIDSSHQDLKLDSGVSTALSKSEVESDKSKLGHGKYYTEKVPYGYNYADKNDQIVDNGCG 283
DSM20080            224 GIDSSHQDLKLDSGVSTALSKSEVESDKSKLGHGKYYTEKVPYGYNYADKNDQIVDNGCG 283
KLDS1.0207          224 GIDSSHQDLKLDSGVSTALSKSEVESDKSKLGHGKYYTEKVPYGYNYADKNDQIVDNGCG 283
KLDS1.1011          224 GIDSSHQDLKLDSGVSTALSKSEVESDKSKLGHGKYYTEKVPYGYNYADKNDQIVDNGCG 283
LBA-40              224 GIDSSHQDLKLDSGVSTALSKSEVESDKSKLGHGKYYTEKVPYGYNYADKNDQIVDNGCG 283
LDB-C1              224 GIDSSHQDLKLDSGVSTALSKSEVESDKSKLGHGKYYTEKVPYGYNYADKNDQIVDNGCG 283
L99                 224 GIDSSHQDLKLDSGVSTALSKSEVESDKSKLGHGKYYTEKVPYGYNYADKNDQIVDNGCG 283
LJJ                 224 GIDSSHQDLKLDSGVSTALSKSEVESDKSKLGHGKYYTEKVPYGYNYADKNDQIVDNGCG 283
MN-BM-F01           224 GIDSSHQDLKLDSGVSTALSKSEVESDKSKLGHGKYYTEKVPYGYNYADKNDQIVDNGCG 283
ND04                224 GIDSSHQDLKLDSGVSTALSKSEVESDKSKLGHGKYYTEKVPYGYNYADKNDQIVDNGCG 283
VHProbi-R03         224 GIDSSHQDLKLDSGVSTALSKSEVESDKSKLGHGKYYTEKVPYGYNYADKNNQIVDNGSG 283
ATCC9649            224 GIDSSHQDLKLDSGVSTALSKSEVESDKSKLGHGNYYTEKVPYGYNYADKNDQIVDNGSG 283
TUA4408L            224 GIDSSHQDLKLDSGVSTALSKSEVESDKSKLGHGKYYTEKVPYGYNYADKNDQIVDNGSG 283
CIDCA133            224 GIDSSHQDLKLDSGVSTALSKSRVESDKSKLGHGKYYTEKVPYGYNYADKNDQIVDNGSG 283
CRL581              224 GIDSSHQDLKLDSGVSTALSKSEVESDKSKLGHGKYYTEKVPYGYNYADKNDQIVDNGSG 283
DSM20072            224 GIDSSHQDLKLDSGVSTALSKSEVESDKSKLGHGKYYTEKVPYGYNYADKNDQIVDNGSG 283
KCCM34717           224 GIDSSHQDLKLDSGVSTALSKSEVESDKSKLGHGKYYTEKVPYGYNYADKNDQIVDNGSG 283
KCTC3034            224 GIDSSHQDLKLDSGVSTALSKSEVESDKSKLGHGKYYTEKVPYGYNYADKNDQIVDNGSG 283
MAG_rmk202_ldel     224 GIDSSHQDLKLDSGVSTALSKSEVESDKSKLGHGKYYTEKVPYGYNYADKNDQIVDNGSG 283
NWC_1_2             224 GIDSSHQDLKLDSGVSTALSKSEVESDKSKLGHGKYYTEKVPYGYNYADKNDQIVDNGSG 283
ND02                224 GIDSSHQDLKLDSGVSTALSKSRVESDKSKLGHGKYYTEKVPYGYNYADKNDQIVDNGSG 283
SK11                219 GIDPTHKDMRLSDDKDVKLTKSDVEKFTDTAKHGRYFNSKVPYGFNYADNNDTITDDTVD 278

2038                284 EMHGQHVAGIAGANGQ-------VKGVAPDAQLLAMKVFSNNAKNSGAYDDDIISAIEDS 336
ACA-DC87            284 EMHGQHVAGIAGANGQ-------VKGVAPNAQLLAMKVFSNNAKNSGAYDDDIISAIEDS 336
ATCC11842           284 EMHGQHVAGIAGANGQ-------VKGVAPDAQLLAMKVFSNNAKNSGAYDDDIISAIEDS 336
ATCC-BAA-365        284 EMHGQHVAGIAGANGQ-------VKGVAPDAQLLAMKVFSNNAKNSGAYDDDIISAIEDS 336
DSM20080            284 EMHGQHVAGIAGANGQ-------VKGVAPDAQLLAMKVFSNNAKNSGAYDDDIISAIEDS 336
KLDS1.0207          284 EMHGQHVAGIAGANGQ-------VKGVAPDAQLLAMKVFSNNAKNSGAYDDDIISAIEDS 336
KLDS1.1011          284 EMHGQHVAGIAGANGQ-------VKGVAPDAQLLAMKVFSNNAKNSGAYDDDIISAIEDS 336
LBA-40              284 EMHGQHVAGIAGANGQ-------VKGVAPDAQLLAMKVFSNNAKNSGAYDDDIISAIEDS 336
LDB-C1              284 EMHGQHVAGIAGANGQ-------VKGVAPDAQLLAMKVFSNNAKNSGAYDDDIISAIEDS 336
L99                 284 EMHGQHVAGIAGANGQ-------VKGVAPDAQLLAMKVFSNNAKNSGAYDDDIISAIEDS 336
LJJ                 284 EMHGQHVAGIAGANGQ-------VKGVAPDAQLLAMKVFSNNAKNSGAYDDDIISAIEDS 336
MN-BM-F01           284 EMHGQHVAGIAGANGQ-------VKGVAPDAQLLAMKVFSNNAKNSGAYDDDIISAIEDS 336
ND04                284 EMHGQHVAGIAGANGQ-------VKGVAPDAQLLAMKVFSNNAKNSGAYDDDIISAIEDS 336
VHProbi-R03         284 EMHGQHVAGIAGANGQ-------VKGVAPDAQLLAMKVFSNNAKNSGAYDDDIISAIEDS 336
ATCC9649            284 EMHGQHVAGIAGANGQ-------VKGVAPDAQLLAMKVFSNNTKNSGAYDDDIISAIEDS 336
TUA4408L            284 EMHGQHVAGIAGANGQ-------VKGVAPDAQLLAMKVFSNNAKNSGAYDDDIISAIEDS 336
CIDCA133            284 EMHGQHVAGIAGANGQ-------VKGVAPDAQLLAMKVFSNNAKNSGAYDDDIISAIEDS 336
CRL581              284 EMHGQHVAGIAGANGQ-------VKGVAPDAQLLAMKVFSNNAKNSGAYDDDIISAIEDS 336
DSM20072            284 EMHGQHVAGIAGANGQ-------VKGVAPDAQLLAMKVFSNNAKNSGAYDDDIISAIEDS 336
KCCM34717           284 EMHGQHVAGIAGANGQ-------VKGVAPDAQLLAMKVFSNNAKNSGAYDDDIISAIEDS 336
KCTC3034            284 EMHGQHVAGIAGANGQ-------VKGVAPDAQLLAMKVFSNNAKNSGAYDDDIISAIEDS 336
MAG_rmk202_ldel     284 EMHGQHVAGIAGANGQ-------VKGVAPDAQLLAMKVFSNNAKNSGAYDDDIISAIEDS 336
NWC_1_2             284 EMHGQHVAGIAGANGQ-------VKGVAPDAQLLAMKVFSNNAKNSGAYDDDIISAIEDS 336
ND02                284 EMHGQHVAGIAGANGQ-------VKGVAPDAQLLAMKVFSNNAKNSGAYDDDIISAIEDS 336
SK11                279 EQHGMHVAGIIGANGTGDDPAKSVVGVAPEAQLLAMKVFTNSDTSATTGSATLVSAIEDS 338
```

```
2038               337 VKLGADVINMSLGSVSSDVDPSDPQQQAVAKASEAGVINVISAGNSGVAGSTADGNPVNN 396
ACA-DC87           337 VKLGADVINMSLGSVSSDVDPSDPQQQAVAKASEAGVINVISAGNSGVAGSTADGNPVNN 396
ATCC11842          337 VKLGADVINMSLGSVSSDVDPSDPQQQAVAKASEAGVINVISAGNSGVAGSTADGNPVNN 396
ATCC-BAA-365       337 VKLGADVINMSLGSVSSDVDPSDPQQQAVAKASEAGVINVISAGNSGVAGSTADGNPVNN 396
DSM20080           337 VKLGADVINMSLGSVSSDVDPSDPQQQAVAKASEAGVINVISAGNSGVAGSTADGNPVNN 396
KLDS1.0207         337 VKLGADVINMSLGSVSSDVDPSDPQQQAVAKASEAGVINVISAGNSGVAGSTADGNPVNN 396
KLDS1.1011         337 VKLGADVINMSLGSVSSDVDPSDPQQQAVAKASEAGVINVISAGNSGVAGSTADGNPVNN 396
LBA-40             337 VKLGADVINMSLGSVSSDVDPSDPQQQAVAKASEAGVINVISAGNSGVAGSTADGNPVNN 396
LDB-C1             337 VKLGADVINMSLGSVSSDVDPSDPQQQAVAKASEAGVINVISAGNSGVAGSTADGNPVNN 396
L99                337 VKLGADVINMSLGSVSSDVDPSDPQQQAVAKASEAGVINVISAGNSGVAGSTADGNPVNN 396
LJJ                337 VKLGADVINMSLGSVSSDVDPSDPQQQAVAKASEAGVINVISAGNSGVAGSTADGNPVNN 396
MN-BM-F01          337 VKLGADVINMSLGSVSSDVDPSDPQQQAVAKASEAGVINVISAGNSGVAGSTADGNPVNN 396
ND04               337 VKLGADVINMSLGSVSSDVDPSDPQQQAVAKASEAGVINVISAGNSGVAGSTADGNPVNN 396
VHProbi-R03        337 VKLGADVINMSLGSVSSDVDPSDPQQQAVAKASEAGVINVISAGNSGVAGSTADGNPVNN 396
ATCC9649           337 VKLGANVINMSLGSVSSDVDPSDPQQQAVAKASEAGVINVISAGNSGVAGSTGDGNPVNN 396
TUA4408L           337 VKLGANVINMSLGSVSSDVDPSDPQQQAVAKASEAGVINVISAGNSGVAGSTGDGNPVNN 396
CIDCA133           337 VKLGADVINMSLGSVSSDVDPSDPEQQAVAKASEAGVINVISAGNSGVAGSTGDGNPVNN 396
CRL581             337 VKLGANVINMSLGSVSSDVDPSDPQQQAVAKASEAGVINVISAGNSGVAGSTADGNPVNN 396
DSM20072           337 VKLGANVINMSLGSVSSDVDPSDPQQQAVAKASEAGVINVISAGNSGVAGSTADGNPVNN 396
KCCM34717          337 VKLGANVINMSLGSVSSDVDPSDPQQQAVAKASEAGVINVISAGNSGVAGSIADGNPVNN 396
KCTC3034           337 VKLGANVINMSLGSVSSDVDPSDPQQQAVAKASEAGVINVISAGNSGVAGSIADGNPVNN 396
MAG_rmk202_ldel    337 VKLGANVINMSLGSVSSDVDPSDPQQQAVAKASEAGVINVISAGNSGVAGSTADGNPVNN 396
NWC_1_2            337 VKLGANVINMSLGSVSSDVDPSDPQQQAVAKASEAGVINVISAGNSGVAGSTADGNPVNN 396
ND02               337 VKLGADVINMSLGSVSSDVDPSDPEQQAVAKASEAGVINVISAGNSGVAGSTGDGNPVNN 396
SK11               339 AKIGADVLNMSLGSDSGNQTLEDPELAAVQNANESGTAAVISAGNSGTSGSATEGVNKDY 398
```

```
2038               397 TGTSELSTVGTPGVTPDALTVASAENSKVTTDTVKDELGGVTFSSNSELKGA----AQVT 452
ACA-DC87           397 TGTSELSTVGTPGVTPDALTVASAENSKVTTDTVKDELGGVIFSSNSELKGA----AQVT 452
ATCC11842          397 TGTSELSTVGTPGVTPDALTVASAENSKVTTDTVKDELGGVTFSSNSELKGA----AQVT 452
ATCC-BAA-365       397 TGTSELSTVGTPGVTPDALTVASAENSKVTTDTVKDELGGVTFSSNSELKGA----AQVT 452
DSM20080           397 TGTSELSTVGTPGVTPDALTVASAENSKVTTDTVKDELGGVTFSSNSELKGAAQGAAQVT 456
KLDS1.0207         397 TGTSELSTVGTPGVTPDALTVASAENSKVTTDTVKDELGGVTFSSNSELKGAAQGAAQVT 456
KLDS1.1011         397 TGTSELSTVGTPGVTPDALTVASAENSKVTTDTVKDELGGVTFSSNSELKGA----AQVT 452
LBA-40             397 TGTSELSTVGTPGVTPDALTVASAENSKVTTDTVKDELGGVTFSSNSELKGA----AQVT 452
LDB-C1             397 TGTSELSTVGTPGVTPDALTVASAENSKVTTDTVKDELGGVTFSSNSELKGAAQGAAQVT 456
L99                397 TGTSELSTVGTPGVTPDALTVASAENSKVTTDTVKDELGGVTFSSNSELKGA----AQVT 452
LJJ                397 TGTSELSTVGTPGVTPDALTVASAENSKVTTDTVKDELGGVTFSSNSELKGA----AQVT 452
MN-BM-F01          397 TGTSELSTVGTPGVTPDALTVASAENSKVTTDTVKDELGKVTFSSNSELKGA----AQVT 452
ND04               397 TGTSELSTVGTPGVTPDALTVASAENSKVTTDTVKDELGGVTFSSNSELKGA----AQVT 452
VHProbi-R03        397 TGTSELSTVGTPGVTPDALTVASAENSKVTTDTVKDELGGVTFSSNSELKGA----AQVT 452
ATCC9649           397 TGTSELSTVGAPGVTPDALTVASAENSKVTTDTVKDELGGVTFSANSELKGA----AQVT 452
TUA4408L           397 TGTSELSTVGAPGVTPDALTVASAENSKVTTDTVKDELGGVTFSSNSELKGA----AQVT 452
CIDCA133           397 TGTSELSTVGTPGVTPDALTVASAENSKVTTDTVKDELGGITFSANPELKGA----AQVT 452
CRL581             397 TGTSELSTVGAPGVTPDALTVASAENSKVTTDTVKDELGGVTFSANSELKGA----AQVT 452
DSM20072           397 TGTSELSTVGAPGVTPDALTVASAENSKVTTDTVKDELGGVTFSANSELKGA----AQVT 452
KCCM34717          397 TGTSELSTVGAPGVTPDALTVASAENSKVTTDTVKDELGGVTFSSNSELKGA----AQVT 452
KCTC3034           397 TGTSELSTVGAPGVTPDALTVASAENSKVTTDTVKDELGGVTFSSNSELKGA----AQVT 452
MAG_rmk202_ldel    397 TGTSELSTVGAPGVTPDALTVASAENSKVITDTVKDELGGVTFSANSELKGA----AQVT 452
NWC_1_2            397 TGTSELSTVGAPGVTPDALTVASAENSKVITDTVKDELGGVTFSANSELKGA----AQVT 452
ND02               397 TGTSELSTVGTPGVTPDALTVASAENSKVTTDTVKDELGGITFSANPELKGA----AQVT 452
SK11               399 YGLQDNEMVGSPGTSRGATTVASAENTDVITQAVTI-------TDGTGLQLG----P-ET 446
```

```
2038              453 TQLES---NYSVLTKKLKLVDMGLGGAD-DYTAEKKAEVKGQLAVVKRGAYTFSAKVANA 508
ACA-DC87          453 TQLES---NYSVLTKKLKLVDMGFGGAD-DYTAEKKAEVKGQLAVVERGSYTFSAKVANA 508
ATCC11842         453 TQLES---NYSVLTKKLKLVDMGLGGAD-DYTAEKKAEVKGQLAVVERGSYTFSAKVANA 508
ATCC-BAA-365      453 TQLES---NYSVLTKKLKLVDMGLGGAD-DYTAEKKAEVKGQLAVVERGSYTFSAKVANA 508
DSM20080          457 TQLES---NYSVLTKKLKLVDMGLGGAD-DYTAEKKAEVKGQLAVVKRGAYTFSAKVANA 512
KLDS1.0207        457 TQLES---NYSVLTKKLKLVDMGLGGAD-DYTAEKKAEVKGQLAVVKRGAYTFSAKVANA 512
KLDS1.1011        453 TQLES---NYSVLTKKLKLVDMGLGGAD-DYTAEKKAEVKGQLAVVKRGAYTFSAKVANA 508
LBA-40            453 TQLES---NYSVLTKKLKLVDMGLGGAD-DYTAEKKAEVKGQLAVVERGSYTFSAKVANA 508
LDB-C1            457 TQLES---NYSVLTKKLKLVDMGLGGAD-DYTAEKKAEVKGQLAVVKRGAYTFSAKVANA 512
L99               453 TQLES---NYSVLTKKLKLVDMGLGGAD-DYTAEKKAEVKGQLAVVKRGAYTFSAKVANA 508
LJJ               453 TQLES---NYSVLTKKLKLVDMGLGGAD-DYTAEKKAEVKGQLAVVERGSYTFSEKVANA 508
MN-BM-F01         453 TQLES---NYSVLTKKLKLVDMGFGGAD-DYTAEKKAEVKGQLAVVERGSYTFSAKVANA 508
ND04              453 TQLES---NYSVLTKKLKLVDMGLGGAD-DYTAEKEAEVKGQLAVVERGSYTFSEKVANA 508
VHProbi-R03       453 TQLES---NYSVLTKKLKLVDMGLGGAD-DYTAEKKAEVKGQLAVVKRGAYTFSEKVANA 508
ATCC9649          453 TQLES---NYSVLTKKLKLVDMGLGGAD-DYTAEKKAEVKGQLAVVKRGSYTFSEKVANA 508
TUA4408L          453 TQLES---NYSVLTKKLKLVNMGLGGAD-DYTAEKKAEVKGQLAVVKRGSYTFSEKVANA 508
CIDCA133          453 TQLES---NYSVLTKKLKLVDMGLGGAD-DYTAEKKAEVKGQLAVVERGSYTFSEKVANA 508
CRL581            453 TQLES---NYSVLTKKLKLVDMGLGGAD-DYTAEKKAEVKGQLAVVKRGSYTFSEKVANA 508
DSM20072          453 TQLES---NYSVLTKKLKLVDMGLGGAD-DYTAEKKAEVKGQLAVVKRGSYTFSEKVANA 508
KCCM34717         453 TQLES---NYSVLTKKLKLVDMGLGGAD-DYTAEKKAEVKGQLAVVKRGSYTFSEKVANA 508
KCTC3034          453 TQLES---NYSVLTKKLKLVDMGLGGAD-DYTAEKKAEVKGQLAVVKRGSYTFSEKVANA 508
MAG_rmk202_ldel   453 TQLES---NYSVLTKKLKLVDMGLGGAD-DYTAEKKAEVKGQLAVVKRGSYTFSEKVANA 508
NWC_1_2           453 TQLES---NYSVLTKKLKLVDMGLGGAD-DYTAEKKAEVKGQLAVVKRGSYTFSEKVANA 508
ND02              453 TQLES---NYSVLTKKLKLVDMGLGGAD-DYTAEKKAEVKGQLAVVERGSYTFSEKVANA 508
SK11              447 IQLSSHDFTGSFDQKKFYIVKDASGNLSKGALADYTADAKGKIAIVKRGEFSFDDKQKYA 506
```

```
2038              509 KAAGAAGIVIYNS-EDDG--LLSMSLDDKTFPTLGMSKADGEVLAK--AAKEGKSIKLKF 563
ACA-DC87          509 KAAGAAGIVIYNNSKDDG--LLSMALDDKTFPTLGMSKADGEVLAQ--AAQKGKSIKLKF 564
ATCC11842         509 KAAGAAGIVIYNS-EDDG--LLSMSLDDKTFPTLGMSKADGEVLAK--AAKEGKSIKLKF 563
ATCC-BAA-365      509 KAAGAAGIVIYNS-EDDG--LLSMSLDDKTFPTLGMSKADGEVLAK--AAKEGKSIKLKF 563
DSM20080          513 KAAGAAGIVIYNN-NDDG--LLSMSLDDKTFPTLGMSKADGEVLAQ--AAKEGKSIKLKF 567
KLDS1.0207        513 KAAGAAGIVIYNN-NDDG--LLSMSLDDKTFPTLGMSKADGEVLAQ--AAKEGKSIKLKF 567
KLDS1.1011        509 KAAGAAGIVIYNS-EDDG--LLSMSLDDKTFPTLGMSKADGEVLAK--AAKEGKSIKLKF 563
LBA-40            509 KAAGAAGIVIYNS-EDDG--LLSMSLDDKTFPTLGMSKADGEVLAK--AAKEGKSIKLKF 563
LDB-C1            513 KAAGAAGIVIYNN-NDDG--LLSMSLDDKTFPTLGMSKADGEVLAQ--AAKEGKSIKLKF 567
L99               509 KAAGAAGIVIYNS-EDDG--LLSMSLDDKTFPTLGMSKADGEVLAK--AAKEGKSIKLKF 563
LJJ               509 KAAGAAGIVIYNN-NDDG--LLSMSLDDKTFPTLGMSKADGEVLAK--AAKEGKSIKLKF 563
MN-BM-F01         509 KAAGAAGIVIYNN-NDDG--LLSMALDDKTFPTLGMSKADGEVLAQ--AAKEGKSIKLKF 563
ND04              509 KAAGAAGIVIYNN-NDDG--LLSMSLDDKTFPTLGMSKADGEVLAK--AAKEGKSIKLKF 563
VHProbi-R03       509 KAAGAAGIVIYNN-NDDG--LLSMSLDDKTFPTLGMSKADGEVLAQ--AAKEGKSIKLKF 563
ATCC9649          509 KAAGAAGIVIYNN-NNDG--LLSMALDDKTFPTLGMSMADGEVLAQ--AAKDGKSIKLKF 563
TUA4408L          509 KAAGAAGIVIYNN-KNDG--LLSMALDDKTFPTLGMSKADGEVLAQ--AANDGKSIKLKF 563
CIDCA133          509 KAAGAAGIVIYNS-KDDG--LLSMSLDDKTFPTLGMSKADGEVLAN--AAKEGKSIKLKF 563
CRL581            509 KAAGAAGIVIYNN-NDDG--LLSMALDDKTFPTLGMSKADGEVLAQ--AAKDGKSIKLKF 563
DSM20072          509 KAAGAAGIVIYNN-NDDG--LLSMALDDKTFPTLGMSKADGEVLAQ--AAKDGKSIKLKF 563
KCCM34717         509 KAAGAAGIVIYNN-NDDG--LLSMALDDKTFPTLGMSKADGEVLAQ--AAKDGKSIKLKF 563
KCTC3034          509 KAAGAAGIVIYNN-NDDG--LLSMALDDKTFPTLGMSKADGEVLAQ--AAKDGKSIKLKF 563
MAG_rmk202_ldel   509 KAAGAAGIVIYNN-NDDG--LLSMALDDKTFPTLGMSKADGEVLAQ--AAKDGKSIKLKF 563
NWC_1_2           509 KAAGAAGIVIYNN-NDDG--LLSMALDDKTFPTLGMSKADGEVLAQ--AAKDGKSIKLKF 563
ND02              509 KAAGAAGIVIYNS-KDDG--LLSMSLDDKTFPTLGMSKADGEVLAN--AAKEGKSIKLKF 563
SK11              507 QAAGAAGLIIVNT-DGTATPMTSIAL-TTTFPTFGLSSVTGQKLVDWVTAHPDDSLGVKI 564
```

```
2038              564 GTALIDNS--SAGKMSDFTSWGPTPDLDFKPEITAPGGKIYSLANDNKYQQMSGTSMASP 621
ACA-DC87          565 GTALIDNS--SAGKMSDFTSWGPTPELDFKPEITAPGGKIYSLANDNKYQQMSGTSMASP 622
ATCC11842         564 GTALIDNS--SAGKMSDFTSWGPTPELDFKPEITAPGGKIYSLANDNKYQQMSGTSMASP 621
ATCC-BAA-365      564 GTALIDNS--SAGKMSDFTSWGPTPDLDFKPEITAPGGKIYSLANDNKYQQMSGTSMASP 621
DSM20080          568 GTALIDNS--SAGKMSDFTSWGPTPELDFKPEITAPGGKIYSLANDNKYQQMSGTSMASP 625
KLDS1.0207        568 GTALIDNS--SAGKMSDFTSWGPTPELDFKPEITAPGGKIYSLANDNKYQQMSGTSMASP 625
KLDS1.1011        564 GTALIDNS--SAGKMSDFTSWGPTPELDFKPEITAPGGKIYSLANDNKYQQMSGTSMASP 621
LBA-40            564 GTALIDNS--SAGKMSDFTSWGPTPDLDFKPEITAPGGKIYSLANDNKYQQMSGTSMASP 621
LDB-C1            568 GTALIDNS--SAGKMSDFTSWGPTPELDFKPEITAPGGKIYSLANDNKYQQMSGTSMASP 625
L99               564 GTALIDNS--SAGKMSDFTSWGPTPELDFKPEITAPGGKIYSLANDNKYQQMSGTSMASP 621
LJJ               564 GTALIDNS--SAGKMSDFTSWGPTPELDFKPEITAPGGKIYSLANDNKYQQMSGTSMASP 621
MN-BM-F01         564 GTALIDNS--SAGKMSDFTSWGPTPDLDFKPEITAPGGKIYSLANDNKYQQMSGTSMASP 621
ND04              564 GTALIDNS--IAGKMSDFTSWGPTPDLDFKPEITAPGGKIYSLANDNKYQQMSGTSMASP 621
VHProbi-R03       564 GTALIDNS--SAGKMSDFTSWGPTPDLDFKPEITAPGGKIYSLANDNKYQQMSGTSMASP 621
ATCC9649          564 GTALIDNS--SAGKMSEFTSWGPTPELDFKPEITAPGGKIYSLANDNKYQQMSGTSMASP 621
TUA4408L          564 GTALIDNS--SAGKMSDFTSWGPTPELDFKPEITAPGGKIYSLANDNKYQQMSGTSMASP 621
CIDCA133          564 GTALIDNP--SAGKMSDFTSWGPTPELDFKPEITAPGGKIYSLANDNKYQQMSGTSMASP 621
CRL581            564 GTALIDNS--SAGKMSDFTSWGPTPELDFKPEITAPGGKIYSLANDNKYQQMSGTSMASP 621
DSM20072          564 GTALIDNS--SAGKMSDFTSWGPTPELDFKPEITAPGGKIYSLANDNKYQQMSGTSMASP 621
KCCM34717         564 GTALIDNS--SAGKMSDFTSWGPTPELDFKPEITAPGGKIYSLANDNKYQQMSGTSMASP 621
KCTC3034          564 GTALIDNS--SAGKMSDFTSWGPTPELDFKPEITAPGGKIYSLANDNKYQQMSGTSMASP 621
MAG_rmk202_ldel   564 GTALIDNS--SAGKMSDFTSWGPTPELDFKPEITAPGGKIYSLANDNKYQQMSGTSMASP 621
NWC_1_2           564 GTALIDNS--SAGKMSDFTSWGPTPELDFKPEITAPGGKIYSLANDNKYQQMSGTSMASP 621
ND02              564 GTALIDNP--SAGKMSDFTSWGPTPELDFKPEITAPGGKIYSLANDNKYQQMSGTSMASP 621
SK11              565 TLAMLPNQKYTEDKMSDFTSYGPVSNLSFKPDITAPGGNIWSTQNNNGYTNMSGTSMASP 624
```

```
2038              622 FVAGSEALILQGIKKQG-------LNLSGEELVQFAKNSAMNTSHPVYDTEHTKEIISPR 674
ACA-DC87          623 FVAGSEALILQGIKKQG-------LNLSGEELVQFAKNSAMNTSHPVYDTEHTKEIISPR 675
ATCC11842         622 FVAGSEALILQGIKKQG-------LNLSGEELVQFAKNSAMNTSHPVYDTEHTKEIISPR 674
ATCC-BAA-365      622 FVAGSEALILQGIKKQG-------LNLSGEELVQFAKNSAMNTSHPVYDTEHTKEIISPR 674
DSM20080          626 FVAGSEALILQGIKKQG-------LNLSGEELVQFAKNSAMNTSHPVHDTEHTKEIISPR 678
KLDS1.0207        626 FVAGSEALILQGIKKQG-------LNLSGEELVQFAKNSAMNTSHPVHDTEHTKEIISPR 678
KLDS1.1011        622 FVAGSEALILQGIKKQG-------LNLSGEELVQFAKNSAMNTSHPVYDTEHTKEIISPR 674
LBA-40            622 FVAGSEALILQGIKKQG-------LNLSGEELVQFAKNSAMNTSHPVYDTEHTKEIISPR 674
LDB-C1            626 FVAGSEALILQGIKKQG-------LNLSGEELVQFAKNSAMNTSHPVHDTEHTKEIISPR 678
L99               622 FVAGSEALILQGIKKQG-------LNLSGEELVQFAKNSAMNTSHPVYDTEHTKEIISPR 674
LJJ               622 FVAGSEALILQGIKKQG-------LNLSGEELVQFAKNSAMNTSHPVYDTEHTKEIISPR 674
MN-BM-F01         622 FVAGSEALILQGIKKQG-------LNLSGEELVQFAKNSAMNTSHPVYDTEHTKEIISPR 674
ND04              622 FVAGSEALILQGIKKQG-------LNLSGEELVQFAKNSAMNTSHPVYDTEHTKEIISPR 674
VHProbi-R03       622 FVAGSEALILQGIKKQG-------LNLSGEELVQFAKNSAMNTSHPVYDTEHTKEIISPR 674
ATCC9649          622 FVAGSEALILQGIKKQG-------LNLSGEELVQFAKNSAMNTSHPFYDTEHTKEIISPR 674
TUA4408L          622 FVAGSEALILQGIKKQG-------LNLSGEELVQFAKNSAMNTSHPFYDTEHTKEIISPR 674
CIDCA133          622 FVAGSEALILQGIKKQG-------LNLSGEELVQFAKNSAMNTSHPVYDTEHTKEIISPR 674
CRL581            622 FVAGSEALILQGIKKQG-------LNLSGEELVQFAKNSAMNTSHPVYDTEHTKEIISPR 674
DSM20072          622 FVAGSEALILQGIKKQG-------LNLSGEELVQFAKNSAMNTSHPVYDTEHTKEIISPR 674
KCCM34717         622 FVAGSEALILQGIKKQG-------LNLSGEELVQFAKNSAMNTSHPVYDTEHTKEIISPR 674
KCTC3034          622 FVAGSEALILQGIKKQG-------LNLSGEELVQFAKNSAMNTSHPVYDTEHTKEIISPR 674
MAG_rmk202_ldel   622 FVAGSEALILQGIKKQG-------LNLSGEELVQFAKNSAMNTSHPFYDTEHTKEIISPR 674
NWC_1_2           622 FVAGSEALILQGIKKQG-------LNLSGEELVQFAKNSAMNTSHPFYDTEHTKEIISPR 674
ND02              622 FVAGSEALILQGIKKQG-------LNLSGEELVQFAKNSAMNTSHPVYDTEHTKEIISPR 674
SK11              625 FIAGSQALLKQALNNKNNPFYAYYKQLKGTALTDFLKTVEMNTAQPINDINYNNVIVSPR 684
```

```
2038               675 RQGSGEINVKDAIN----NTVEVKAANGNGAAALKEIG-RQTTFKVTLTNHGKKAQTYAV 729
ACA-DC87           676 RQGSGEINVKDAIN----NTVEVKAANGNGAAALKEIG-RQTTFKVTLTNHGKKAQTYAV 730
ATCC11842          675 RQGSGEINVKDAIN----NTVEVKAANGNGAAALKEIG-RQTTFKVTLTNHGKKAQTYAV 729
ATCC-BAA-365       675 RQGSGEINVKDAIN----NTVEVKAANGNGAAALKEIG-RQTTFKVTLTNHGKKAQTYAV 729
DSM20080           679 RQGSGEINVKDAIN----NTVEVKAANGNGAAALKEIG-RQTTFKVTLTNHGKKAQTYAV 733
KLDS1.0207         679 RQGSGEINVKDAIN----NTVEVKAANGNGAAALKEIG-RQTTFKVTLTNHGKKAQTYAV 733
KLDS1.1011         675 RQGSGEINVKDAIN----NTVEVKAANGNGAAALKEIG-RQTTFKVTLTNHGKKTQTYAV 729
LBA-40             675 RQGSGEINVKDAIN----NTVEVKAANGNGAAALKEIG-RQTTFKVTLTNHGKKAQTYAV 729
LDB-C1             679 RQGSGEINVKDAIN----NTVEVKAANGNGAAALKEIG-RQTTFKVTLTNHGKKAQTYAV 733
L99                675 RQGSGEINVKDAIN----NTVEVKAANGNGAAALKEIG-RQTTFKVTLTNHGKKAQTYAV 729
LJJ                675 RQGSGEINVKDAIN----NTVEVKAANGNGAAALKEIG-RQTTFKVTLTNHGKKAQTYAV 729
MN-BM-F01          675 RQGSGEINVKDAIN----NTVEVKAANGNGAAALKEIG-RQTTFKVTLTNHGKKAQTYAV 729
ND04               675 RQGSGEINVKDAIN----NTVEVKAANGNGAAALKEIG-RQTTFKVTLTNHGKKAQTYAV 729
VHProbi-R03        675 RQGSGEINVKDAIN----NTVEVKAANGNGAAALKEIG-RQTTFKVTLTNHGKKAQTYAV 729
ATCC9649           675 RQGSG------------------------------------------------------- 679
TUA4408L           675 RQGSGEINVKDAIN----NTVEVKAANGNGAAALKEIG-RQTTFKVTLTNYGKKAQTYAV 729
CIDCA133           675 RQGSGEINVKDAIN----NTVEVKAANGNGAAALKEIG-RQTTFKVTLTNHGKKAQTYAV 729
CRL581             675 RQGSGEINVKDAIN----NTVEVKAANGNGAAALKEIG-RQTTFKVTLTNHGKKAQTYAV 729
DSM20072           675 RQGSGEINVKDAIN----NTVEVKAANGNGAAALKEIG-RQTTFKVTLTNHGKKAQTYAV 729
KCCM34717          675 RQGSGEINVKDAIN----NTVEVKAANGNGAAALKEIG-RQTTFKVTLTNHGKKAQTYAV 729
KCTC3034           675 RQGSGEINVKDAIN----NTVEVKAANGNGAAALKEIG-RQTTFKVTLTNHGKKAQTYAV 729
MAG_rmk202_ldel    675 RQGSGEINVKDAIN----NTVEVKAANGNGAAALKEIG-RQTTFKVTLTNHGKKAQTYAV 729
NWC_1_2            675 RQGSGEINVKDAIN----NTVEVKAANGNGAAALKEIG-RQTTFKVTLTNHGKKAQTYAV 729
ND02               675 RQGSGEINVKDAIN----NTVEVKAANGNGAAALKEIG-RQTTFKVTLTNHGKKAQTYAV 729
SK11               685 RQGAGLVDVKAAIDALEKNPSTVVAENGYPAVELKDFTSTDKTFKLTFTNRTTHELTYQM 744

2038               730 DNY---GGPYTQATEAKSGEIYDTKIVKGQLTTETPKVTVQPGESVDVSFTLTLPYSFQR 786
ACA-DC87           731 DNY---GGPYTQATEAKSGEIYDTKIVKGQLTTETPKVTVQPGESVDVSFTLTLPYSFQR 787
ATCC11842          730 DNY---GGPYTQATEAKSGEIYDTKIVKGQLTTETPKVTVQPGESVDVSFTLTLPYSFQR 786
ATCC-BAA-365       730 DNY---GGPYTQATEAKSGEIYDTKIVKGQLTTETPKVTVQPGESVDVSFTLTLPYSFQR 786
DSM20080           734 DNY---GGPYTQATEAKSGEIYDTKIVKGQLTTETPKVTVQPGESVDVSFTLTLPYSFQR 790
KLDS1.0207         734 DNY---GGPYTQATEAKSGEIYDTKIVKGQLTTETPKVTVQPGESVDVSFTLTLPYSFQR 790
KLDS1.1011         730 DNY---GGPYTQATEAKSGEIYDTKIVKGQLTTETPKVTVQPGESVDVSFTLTLPYSFQR 786
LBA-40             730 DNY---GGPYTQATEAKSGEIYDTKIVKGQLTTETPKVTVQPGESVDVSFTLTLPYSFQR 786
LDB-C1             734 DNY---GGPYTQATEAKSGEIYDTKIVKGQLTTETPKVTVQPGESVDVSFTLTLPYSFQR 790
L99                730 DNY---GGPYTQATEAKSGEIYDTKIVKGQLTTETPKVTVQPGESVDVSFTLTLPYSFQR 786
LJJ                730 DNY---GGPYTQATEAKSGEIYDTKIVKGQLTTETPKVTVQPGESVDVSFTLTLPYSFQR 786
MN-BM-F01          730 DNY---GGPYTQATEAKSGEIYDTKIVKGQLTTETPKVTVQPGESVDVSFTLTLPYSFQR 786
ND04               730 DNY---GGPYTQATEAKSGEIYDTKIVKGQLTTETPKVTVQPGESVDVSFTLTLPYSFQR 786
VHProbi-R03        730 DNY---GGPYTQATEAKSGEIYDTKIVKGQLTTETPKVTVQPGESVDVSFTLTLPYSFQR 786
ATCC9649           680 -------------------EIYDTKIVKGQLTTETPKVTVQPGESVDVSFTLTLPYSFQR 720
TUA4408L           730 DNY---GGPYTQATEAKSGEIYDTKIVKGQLTTETPKVTVQPGESVDVSFTLTLPYSFQR 786
CIDCA133           730 DNY---GGPYTQATEAKSGEIYDTKIVKGQLTTETPKVTVQPGESVDVSFTLTLPYSFQR 786
CRL581             730 DNY---GGPYTQATEAKSGEIYDTKIVKGQLTTETPKVTVQPGESVDVSFTLTLPYSFQR 786
DSM20072           730 DNY---GGPYTQATEAKSGEIYDTKIVKGQLTTETPKVTVQPGESVDVSFTLTLPYSFQR 786
KCCM34717          730 DNY---GGPYTQATEAKSGEIYDTKIVKGQLTTETPKVTVQPGESVDVSFTLTLPYSFQR 786
KCTC3034           730 DNY---GGPYTQATEAKSGEIYDTKIVKGQLTTETPKVTVQPGESVDVSFTLTLPYSFQR 786
MAG_rmk202_ldel    730 DNY---GGPYTQATEAKSGEIYDTKIVKGQLTTETPKVTVQPGESVDVSFTLTLPYSFQR 786
NWC_1_2            730 DNY---GGPYTQATEAKSGEIYDTKIVKGQLTTETPKVTVQPGESVDVSFTLTLPYSFQR 786
ND02               730 DNY---GGPYTQATEAKSGEIYDTKIVKGQLTTETPKVTVQPGESVDVSFTLTLPYSFQR 786
SK11               745 DSNTDTNAVYTSATDPNSGVLYDKKI-DGAAIKAGSNITVPAGKTAQIEFTLSLPKSFDQ 803
```

```
2038              787 QNFVEGYVGFEAEDQATPNLVLPYMGFFGSYSQASVSAPMLYEGGNSNLINTIHSLVGVM 846
ACA-DC87          788 QNFVEGYVGFEAEDQATPNLVLPYMGFFGSYSQASVSAPMLYEGGNSNLINTIHSLVGVM 847
ATCC11842         787 QNFVEGYVGFEAKDQATPNLVLPYMGFFGSYSQASVSAPMLYEGGNSNLINTIHSLVGVM 846
ATCC-BAA-365      787 QNFVEGYVGFEAEDQATPNLVLPYMGFFGSYSQASVSAPMLYEGGNSNLINTIHSLVGVM 846
DSM20080          791 QNFVEGYVGFEAEDQATPNLVLPYMGFFGSYSQASVSAPMLYEGGNSNLINTIHSLVGVM 850
KLDS1.0207        791 QNFVEGYVGFEAEDQATPNLVLPYMGFFGSYSQASVSAPMLYEGGNSNLINTIHSLVGVM 850
KLDS1.1011        787 QNFVEGYVGFEAEDQATPNLVLPYMGFFGSYSQASVSAPMLYEGGNSNLINTIHSLVGVM 846
LBA-40            787 QNFVEGYVGFEAEDQATPNLVLPYMGFFGSYSQASVSAPMLYEGGNSNLINTIHSLVGVM 846
LDB-C1            791 QNFVEGYVGFEAEDQATPNLVLPYMGFFGSYSQASVSAPMLYEGGNSNLINTIHSLVGVM 850
L99               787 QNFVEGYVGFEAKDQATPNLVLPYMGFFGSYSQASVSAPMLYEGGNSNLINTIHSLVGVM 846
LJJ               787 QNFVEGYVGFEAEDQATPNLVLPYMGFFGSYSQASVSAPMLYEGGNSNLINTIHSLVGVM 846
MN-BM-F01         787 QNFVEGYVGFEAEDQATPNLVLPYMGFFGSYSQASVSAPMLYEGGNSNLINTIHSLVGVM 846
ND04              787 QNFVEGYVGFEAEDQATPNLVLPYMGFFGSYSQASVSAPMLYEGGNSNLINTIHSLVGVM 846
VHProbi-R03       787 QNFVEGYVGFEAEDQATPNLVLPYMGFFGSYSQASVSAPMLYEGGNSNLINTIHSLVGVM 846
ATCC9649          721 QNFVEGYVGFEAEDQATPNLVLPYMGFFGSYSQASVSAPMLYEGGNSNLINTIQSLVGVM 780
TUA4408L          787 QNFVEGYVGFEAEDQATPNLVLPYMGFFGSYSQASVSAPMLYEGGNSNLINTIQSLVGVM 846
CIDCA133          787 QNFVEGYVGFEAEDQATPNLVLPYMGFFGSYSQASVSAPMLYEGGNSNLINTIQSLVGVM 846
CRL581            787 QNFVEGYVGFEADDRTTPNLVLPYMGFFGSYSQASVSAPMLYEGGNSNLINTIHSLVGVM 846
DSM20072          787 QNFVEGYVGFEAEDRATPNLVLPYMGFFGSYSQASVSAPMLYEVGNSNLINTIHSLVGVM 846
KCCM34717         787 QNFVEGYVGFEAEDRATPNLVLPYMGFFGSYSQASVSAPMLYEGGNSNLINTIHSLVGVM 846
KCTC3034          787 QNFVEGYVGFEAEDRATPNLVLPYMGFFGSYSQASVSAPMLYEGGNSNLINTIHSLVGVM 846
MAG_rmk202_ldel   787 QNFVEGYVGFEAEDQATPNLVLPYMGFFGSYSQASVSAPMLYEGGNSNLINTIHSLVGVM 846
NWC_1_2           787 QNFVEGYVGFEAEDQATPNLVLPYMGFFGSYSQASVSAPMLYEGGNSNLINTIHSLVGVM 846
ND02              787 QNFVEGYVGFEAEDQATPNLVLPYMGFFGSYSQASVSAPMLYEGGNSNLINTIQSLVGVM 846
SK11              804 QQFVEGFLNFKGSDGSR--LNLPYMGFFGDWNDGKIV----------------DSLNGIT 845

2038              847 FSNNNDIL--------------GHTGYE-GDHYSKYTDPDLIAISPNGDGSRDYAYPVLF 891
ACA-DC87          848 FSNNNDML--------------GHTGYE-GDDYSKYTDPDLIAISPNGDGSRDYAYPVLF 892
ATCC11842         847 FSNNNDML--------------GHTGYE-GDDYSKYTDPDLIAISPNGDGSRDYAYPVLF 891
ATCC-BAA-365      847 FSNNNDIL--------------GHTGYE-GDDYSKYTDPDLIAISPNGDGSRDYAYPVLF 891
DSM20080          851 FSNNNDIL--------------GRTGYE-GDDYSKYTDPDLIAISPNGDGSRDYAYPVLF 895
KLDS1.0207        851 FSNNNDIL--------------GRTGYE-GDDYSKYTDPDLIAISPNGDGSRDYAYPVLF 895
KLDS1.1011        847 FSNNNDIL--------------GHTGYE-GDDYSKYTDPDLIAISPNGDGSRDYAYPVLF 891
LBA-40            847 FSNNNDIL--------------GHTGYE-GDDYSKYTDPDLIAISPNGDGSRDYAYPVLF 891
LDB-C1            851 FSNNNDIL--------------GRTGYE-GDDYSKYTDPDLIAISPNGDGSRDYAYPVLF 895
L99               847 FSNNNDML--------------GHTGYE-GDDYSKYTDPDLIAISPNGDGSRDYAYPVLF 891
LJJ               847 FSNNNDIL--------------GHTGYE-GDDYSKYTDPDLIAISPNGDGSRDYAYPVLF 891
MN-BM-F01         847 FSNNKDIL--------------GRTGYE-GDDYSKYTDPDLIAISPNGDGSRDYAYPVLF 891
ND04              847 FSNNNDIL--------------GRTGYE-GDDYSKYTDPDLIAISPNGDGSRDYAYPVLF 891
VHProbi-R03       847 FSNNKDIL--------------GRTGYE-GDDYSKYTDPDLIAISPNGDGSRDYAYPVLF 891
ATCC9649          781 ASNNNDIL--------------GHTGYE-GDDYSKYTDPDLIAISPNGDGSRDYAYPVLF 825
TUA4408L          847 TSNNNDIL--------------GHTGYE-GDDYSKYTDPDLIAISPNGDGSRDYAYPVLF 891
CIDCA133          847 SSNNDDIL--------------GSTGYE-GDDYSKYTDPDLIAISPNGDGSRDYAYPVLF 891
CRL581            847 SSNNNDIL--------------GHTGYE-GDDYSKYTDPDLIAISPNGDGSRDYAYPVLF 891
DSM20072          847 SSNDNDIL--------------GHTGYE-GDDYSKYTDPDLIAISPNGDGSRDYAYPVLF 891
KCCM34717         847 SSNDNDIL--------------GYTG---GDDYSKYTDPDLIAISPNGDGSRDYAYPVLF 889
KCTC3034          847 SSNDNDIL--------------GYTG---GDDYSKYTDPDLIAISPNGDGSRDYAYPVLF 889
MAG_rmk202_ldel   847 SSNNNDIL--------------GHTGYE-GDDYSKYTDPDLIAISPNGDGSRDYAYPVLF 891
NWC_1_2           847 SSNNNDIL--------------GHTGYE-GDDYSKYTDPDLIAISPNGDGSRDYAYPVLF 891
ND02              847 SSNNDDIL--------------GSTGYE-GDDYSKYTDPDLIAISPNGDGSRDYAYPVLF 891
SK11              846 YSPAGGNFGTVPLLKNKNTGTQYYGGMVTDADGNKTVDDQAIAFSSDKNALYNEISMKYY 905
```

```
2038              892 FDRNYKEYTETITDAQGNKVKSLGVGKEGTKDYYSSSSGEWTTHSLDKWDGTDA-----D  946
ACA-DC87          893 FDRNYKEYTETITDAQGNKVKSLGVGKEGTKDYYSSSSGEWTTHSLDKWDGTDA-----D  947
ATCC11842         892 FDRNYKEYTETITDAQGNKVKSLGVGKEGTKDYYSSSSGEWTTHSLDKWDGTDA-----D  946
ATCC-BAA-365      892 FDRNYKEYTETITDAQGNKVKSLGVGKEGTKDYYSSSSGEWTTHSLDKWDGTDA-----D  946
DSM20080          896 FDRNYKEYTETITDAQGNKVKSLGVGKEGTKDYYSSSSGEWTTHSLDKWDGTDA-----D  950
KLDS1.0207        896 FDRNYKEYTETITDAQGNKVKSLGVGKEGTKDYYSSSSGEWTTHSLDKWDGTDA-----D  950
KLDS1.1011        892 FDRNYKEYTETITDAQGNKVKSLGVGKEGTKDYYSSSSGEWTTHSLDKWDGTDA-----D  946
LBA-40            892 FDRNYKEYTETITDAQGNKVKSLGVGKEGTKDYYSSSSGEWTTHSLDKWDGTDA-----D  946
LDB-C1            896 FDRNYKEYTETITDAQGNKVKSLGVGKEGTKDYYSSSSGEWTTHSLDKWDGTDA-----D  950
L99               892 FDRNYKEYTETITDAQGNKVKSLGVGKEGTKDYYSSSSGEWTTHSLDKWDGTDA-----D  946
LJJ               892 FDRNYKEYTETITDAQGNKVKSLGVGKEGTKDYYSSSSGEWTTHSLDKWDGTDA-----D  946
MN-BM-F01         892 FDRNYKEYTETITDAQGNKVKSLGVDKEGTKDYYSSSSGEWTTHSLDKWDGTDA-----D  946
ND04              892 FDRNYKEYTETITDAQGNKVKSLGVDKEGTKDYYSSSSGEWTTHSLDKWDGTDA-----D  946
VHProbi-R03       892 FDRNYKEYTETITDAQGNKVKSLGVDKEGTKDYYSSSSGEWTTHSLDKWDGTDA-----D  946
ATCC9649          826 FDRNYKEYTEMITDAQGNKVKSLGVGKEGTKDYYSSSSGKWTTHSLDKWDGTDA-----D  880
TUA4408L          892 FDRNYKEYTETITDAQGNKVKSLGVGKEGTKDYYSSSSGKWTTHSLDKWDGTDA-----D  946
CIDCA133          892 FDRNYKEYTETITDAQGNKVKSLGVGKKGTKDYYSSSSGKWTTHSLDKWDGTDA-----D  946
CRL581            892 FDRNYKEYTETITDAQGNKVKSLGVGKEGTKDYYSSSSGKWTTHSLDKWDGTDA-----D  946
DSM20072          892 FDRNYKEYTETITDAQGNKVKSLGVGKEGTKDYYSSSSGKWTTHSLDKWDGTDA-----D  946
KCCM34717         890 FDRNYKEYTETITDAQGNKVKSLGVGKEGTKDYYSSSSGKWTTHSLDKWDGTDA-----D  944
KCTC3034          890 FDRNYKEYTETITDAQGNKVKSLGVGKEGTKDYYSSSSGKWTTHSLDKWDGTDA-----D  944
MAG_rmk202_ldel   892 FDRNYKEYTETITDAQGNKVKSLGVGKKGTKDYYSSSRGKWTTHSLDKWDGTDA-----D  946
NWC_1_2           892 FDRNYKEYTETITDAQGNKVKSLGVDKKGTKDYYSSSRGKWTTHSLDKWDGTDA-----D  946
ND02              892 FDRNYKEYTETITDAQGNKVKSLGVGKKGTKDYYSSSSGKWTTHSLDKWDGTDA-----D  946
SK11              906 LLRNISNVQVDILDGQGNKVTTLSSSTNRKKTYYNAHSQQYIYYNAPAWDGTYYDQRDGN  965

2038              947 GQVVKDGQYIYKVEFTPAIGGSKQELNIPVKVDTQAPEVSDLQV---TKDGK----LRLK  999
ACA-DC87          948 GQVVKDGQYIYKVEFTPAIGGSKQELNIPVKVDTQAPEVSDLQV---TKDGK----LRLK 1000
ATCC11842         947 GQVVKDGQYIYKVEFTPAIGGSKQELNIPVKVDTQAPEVSDLQV---TKDGK----LRLK  999
ATCC-BAA-365      947 GQVVKDGQYIYKVEFTPAIGGSKQELNIPVKVDTQAPEVSDLQV---TKDGK----LRLK  999
DSM20080          951 GQVVKDGQYIYKVEFTPAIGGSKQELNIPVKVDTQAPEVSDLQV---TKDGK----LRLK 1003
KLDS1.0207        951 GQVVKDGQYIYKVEFTPAIGGSKQELNIPVKVDTQAPEVSDLQV---TKDGK----LRLK 1003
KLDS1.1011        947 GQVVKDGQYIYKVEFTPAIGGSKQELNIPVKVDTQAPEVSDLQV---TKDGK----LRLK  999
LBA-40            947 GQVVKDGQYIYKVEFTPAIGGSKQELNIPVKVDTQAPEVSDLQV---TKDGK----LRLK  999
LDB-C1            951 GQVVKDGQYIYKVEFTPAIGGSKQELNIPVKVDTQAPEVSDLQV---TKDGK----LRLK 1003
L99               947 GQVVKDGQYIYKVEFTPAIGGSKQELNIPVKVDTQAPEVSDLQV---TKDGK----LRLK  999
LJJ               947 GQVVKDGQYIYKVEFTPAIGGSKQELNIPVKVDTQAPEVSDLQV---TKDGK----LRLK  999
MN-BM-F01         947 GQVVKDGQYIYKVEFIPAIGGSKQELNIPVKVDTQAPEVSDLQV---TKDGK----LRLK  999
ND04              947 GQVVKDGQYIYKVEFTPAIGGSKQELNIPVKVDTQAPEVSDLQV---TKDGK----LRLK  999
VHProbi-R03       947 GQVVKDGQYIYKVEFIPAIGGSKQELNIPVKVDTQAPEVSDLQV---TKDGK----LRLK  999
ATCC9649          881 GQVVKDGQYIYKVEFTPATGGNKQELNIPVKVDTQAPEVSDLQV---TKDGK----LRLK  933
TUA4408L          947 GQVVKDGQYIYKVEFTPATGGSKQELNIPVKVDTQAPEVSDLQV---TKDGK----LRLK  999
CIDCA133          947 GQVVKDGQYIYKVEFTPATGDGKQELNIPVKVDTQAPEVSDLQV---TKDGK----LHLK  999
CRL581            947 GQVVKDGQYIYKVEFTPATGGSKQELNIPVKVDTQAPEVSDLQV---TKDGK----LRLK  999
DSM20072          947 GQVVKDGQYIYKVEFTPATGGSKQELNIPVKVDTQAPEVSDLQV---TKDGK----LRLK  999
KCCM34717         945 GQVVKDGQYIYKVEFTPATGGSKQELNIPVKVDTQAPEVSDLQV---TKDGK----LRLK  997
KCTC3034          945 GQVVKDGQYIYKVEFTPATGGSKQELNIPVKVDTQAPEVSDLQV---TKDGK----LRLK  997
MAG_rmk202_ldel   947 GQVVKDGQYIYKVEFTPATGGSKQELNIPVKVDTQAPEVSDLQV---TKDGK----LRLK  999
NWC_1_2           947 GQVVKDGQYIYKVEFTPATGGSKQELNIPVKVDTQAPEVSDLQV---TKDGK----LRLK  999
ND02              947 GQVVKDGQYIYKVEFTPATGDGKQELNIPVKVDTQAPEVSDLQV---TKDGK----LHLK  999
SK11              966 IKTADDGSYTYRISGVPEGGDKRQVFDVPFKLDSKAPTVRHVALSAKTENGKTQYYLTAE 1025
```

```
2038              1000 AKDSGSGLDMTMFVAAVNGEEQKLALAPVKGE--SNVY------ESTSALTGLKDGKNQV 1051
ACA-DC87          1001 AKDSGSGLDMTMFVAAVNGEEQKLALAPVKGE--SNVY------ESTSALTGLKDGKNQV 1052
ATCC11842         1000 AKDSGSGLDMTMFVAAVNGEEQKLALAPVKGE--SNVY------ESTSALTGLKDGKNQV 1051
ATCC-BAA-365      1000 AKDSGSGLDMTMFVAAVNGEEQKLALAPVKGE--SNVY------ESTSALTGLKDGKNQV 1051
DSM20080          1004 AKDSGSGLDMTMFVAAVNGEEQKLALAPVKG----NVY------ESTSALTGLKDGKNQV 1053
KLDS1.0207        1004 AKDSGSGLDMTMFVAAVNGEEQKLALAPVKG----NVY------ESTSALTGLKDGKNQV 1053
KLDS1.1011        1000 AKDSGSGLDMTMFVAAVNGEEQKLALAPVKGE--SNVY------ESTSALTGLKDGKNQV 1051
LBA-40            1000 AKDSGSGLDMTMFVAAVNGEEQKLALAPVKGE--SNVY------ESTSALTGLKDGKNQV 1051
LDB-C1            1004 AKDSGSGLDMTMFVAAVNGEEQKLALAPVKG----NVY------ESTSALTGLKDGKNQV 1053
L99               1000 AKDSGSGLDMTMFVAAVNGEEQKLALAPVKGE--SNVY------ESTSALTGLKDGKNQV 1051
LJJ               1000 AKDSGSGLDMTMFVAAVNGEEQKLALAPVKGE--SNVY------ESTSALTGLKDGKNQV 1051
MN-BM-F01         1000 AKDSGSGLDMTMFVAAVNGEEQKLALAPVKGE--SNVY------ESTSALTGLKDGKNQV 1051
ND04              1000 AKDSGSGLDMTMFVAAVNGEEQKLALAPVKGE--SNVY------ESTSALTGLKDGKNQV 1051
VHProbi-R03       1000 AKDSGSGLDMTMFVAAVNGEEQKLALAPVKGE--SNVY------ESTSALTGLKDGKNQV 1051
ATCC9649           934 AKDSGSGLDMTMFVAAVNGEKQKLALAPVKGE--SDVY------ESTTALTGLKDGKNQV  985
TUA4408L          1000 AKDSGSGLDDTMFVAAVNGQKQKLALTQVKGE--ENVY------ESTTALTGLKDGKNQV 1051
CIDCA133          1000 AKDTGSGLDMTMFVAVVNGEKQKLALAPVKGE--SDVY------ESTTALTGLKDGKNQV 1051
CRL581            1000 AKDSGSGLDMTMFVAAVNGEKQKLALAPVKGE--SDVY------ESTTALTGLKDGKNQV 1051
DSM20072          1000 AKDSGSGLDMTMFVAAVNGEKQKLALAPVKGE--SDVY------ESTTALTGLKDGKNQV 1051
KCCM34717          998 AKDSGSGLDMTMFVAAVNGEKQKLALAPVKGE--SDVY------ESTTALTGLKDGKNQV 1049
KCTC3034           998 AKDSGSGLDMTMFVAAVNGEKQKLALAPVKGE--SDVY------ESTTALTGLKDGKNQV 1049
MAG_rmk202_ldel   1000 AKDSGSGLDMTMFVAAVNGEKQKLALAPVKGE--SDVY------ESTTALTGLKDGKNQV 1051
NWC_1_2           1000 AKDSGSGLDMTMFVAAVNGEKQKLALAPVKGE--SDVY------ESTTALTGLKDGKNQV 1051
ND02              1000 AKDTGSGLDMTMFVAVVNGEKQKLALAPVKGE--SDVY------ESTTALTGLKDGKNQV 1051
SK11              1026 AKDDLSGLDATKSVKTEINEVTNLDATFTDAGTTADGYTKIETPLSDEQAQALGNGDNSA 1085
```

```
2038              1052 ETVLADYAGNVGYAATFSSQNNDADNKLLLFNLADGQKITSQSPAYDQE---KETYTVTG 1108
ACA-DC87          1053 ETVLADYAGNVGYAATFSSQNNDADNKLLLFNLADGQKITSQSPAYDQE---KETYTVTG 1109
ATCC11842         1052 ETVLADYAGNVGYAATFSSQNNDADNKLLLFNLADGQKITSQSPAYDQE---KETYTVTG 1108
ATCC-BAA-365      1052 ETVLADYAGNVGYAATFSSQNNDADNKLLLFNLADGQKITSQSPAYDQE---KETYTVTG 1108
DSM20080          1054 ETVLADYAGNVGYAATFSSQNNDADNKLLLFNLADGQKITSQSPAYDQE---KETYTVTG 1110
KLDS1.0207        1054 ETVLADYAGNVGYAATFSSQNNDADNKLLLFNLADGQKITSQSPAYDQE---KETYTVTG 1110
KLDS1.1011        1052 ETVLADYAGNVGYAATFSSQNNDADNKLLLFNLADGQKITSQSPAYDQE---KETYTVTG 1108
LBA-40            1052 ETVLADYAGNVGYAATFSSQNNDADNKLLLFNLADGQKITSQSPAYDQE---KETYTVTG 1108
LDB-C1            1054 ETVLADYAGNVGYAATFSSQNNDADNKLLLFNLADGQKITSQSPAYDQE---KETYTVTG 1110
L99               1052 ETVLADYAGNVGYAATFSSQNNDADNKLLLFNLADGQKITSQSPAYDQE---KETYTVTG 1108
LJJ               1052 ETVLADYAGNVGYAATFSSQNNDADNKLLLFNLADGQKITSQSPAYDQE---KETYTVTG 1108
MN-BM-F01         1052 ETVLADYAGNVGYAATFSSQNNDADNKLLLFNLADGQKITSQSPAYDQE---KETYTVTG 1108
ND04              1052 ETVLADYAGNVGYAATFSSQNNDADNKLLLFNLADGQKITSQSPAYDQE---KETYTVTG 1108
VHProbi-R03       1052 ETVLADYAGNVGYAATFSSQNNDADNKLLLFNLADGQKITSQSPAYDQE---KETYTVTG 1108
ATCC9649           986 ETVLADYAGNVGYAATFSSQNNDADNKLLLFNLADGQKITSQSPAYDQE---KETYTVTG 1042
TUA4408L          1052 ETVLADYAGNVGYAATFSSQNNDADNKLLLFNLADGQKITSQSPAYEQE---KETYTVTG 1108
CIDCA133          1052 ETVLADNAGNVGYAATFSSQNDDADNKLLLFNLADGQKITSQSPAYDQE---KGTYTVTG 1108
CRL581            1052 ETVLADYAGNVGYAATFSSQNNDADNKLLLFNLADGQKITSQSPAYDQE---KETYTVTG 1108
DSM20072          1052 ETVLADYAGNVGYAATFSSQNNDADNKLLLFNLADGQKITSQSPAYDQE---KETYTVTG 1108
KCCM34717         1050 ETVLADYAGNVGYAATFSSQNNDADNKLLLFNLADGQKITSQSPAYDQE---KETYTVTG 1106
KCTC3034          1050 ETVLADYAGNVGYAATFSSQNNDADNKLLLFNLADGQKITSQSPAYDQE---KETYTVTG 1106
MAG_rmk202_ldel   1052 ETVVADYAGNVGYAATFSSQNNDADNKLLLFNLADGQKITSQSPAYDQE---KETYTVTG 1108
NWC_1_2           1052 ETVVADYAGNVGYAATFSSQNNDADNKLLLFNLADGQKITSQSPAYDQE---KETYTVTG 1108
ND02              1052 ETVLADNAGNVGYAATFSSQNDDADNKLLLFNLADGQKITSQSPAYDQE---KGTYTVTG 1108
SK11              1086 ELYLTDNASNATDQDASVQKPGSTSFDLIVNGGGIPDKISSTTTGYEANTQGGGTYTFSG 1145
```

```
2038                1109 TYKKNAKLKFNDVEAES-------D-KNGYFEVKLPVKDGQNQL-----LIKDGDQILEA 1155
ACA-DC87            1110 TYKKNAKLKFNDVEAES-------D-KNGYFEVKLPVKDGQNQL-----LIKDGDQILEA 1156
ATCC11842           1109 TYKKNAKLKFNDVEAES-------D-KNGYFEVKLPVKDGQNQL-----LIKDGDQILEA 1155
ATCC-BAA-365        1109 TYKKNAKLKFNDVEAES-------D-KNGYFEVKLPVKDGQNQL-----LIKDGDQILEA 1155
DSM20080            1111 TYKKNAKLKFNDVEAES-------D-KNGYFEVKLPVKDGQNQL-----LIKDGDQILEA 1157
KLDS1.0207          1111 TYKKNAKLKFNDVEAES-------D-KNGYFEVKLPVKDGQNQL-----LIKDGDQILEA 1157
KLDS1.1011          1109 TYKKNAKLKFNDVEAES-------D-KNGYFEVKLPVKDGQNQL-----LIKDGDQILEA 1155
LBA-40              1109 TYKKNAKLKFNDVEAES-------D-KNGYFEVKLPVKDGQNQL-----LIKDGDQILEA 1155
LDB-C1              1111 TYKKNAKLKFNDVEAES-------D-KNGYFEVKLPVKDGQNQL-----LIKDGDQILEA 1157
L99                 1109 TYKKNAKLKFNDVEAES-------D-KNGYFEVKLPVKDGQNQL-----LIKDGDQILEA 1155
LJJ                 1109 TYKKNAKLKFNDVEAES-------D-KNGYFEVKLPVKDGQNQL-----LIKDGDQILEA 1155
MN-BM-F01           1109 TYKKNAKLKFNDVEAES-------D-KNGYFEVKLPVKDGQNQL-----LIKDGDQILEA 1155
ND04                1109 TYKKNAKLKFNDVEAEI-------D-KNGYFEVKLPVKDGQNQL-----LIKDGDQILEA 1155
VHProbi-R03         1109 TYKKNAKLKFNDVEAES-------D-EDGYFEVKLPVKDGQNQL-----LIKDGDQILEA 1155
ATCC9649            1043 TYKKNAKLKFNDVEAES-------D-EDGYFEVKLPVKDGQNQL-----LIKDGDQTLEA 1089
TUA4408L            1109 TYKKNAKLKFNDVEAES-------D-EDGYFEVKLPVKDGQNQL-----LIKDGDQTLEA 1155
CIDCA133            1109 TYKKNAKLKFNDVEAGS-------D-KNGYFEVKLPVKDGQNQL-----LIKDGDQILEA 1155
CRL581              1109 TYKKNAKLKFNDVEAES-------D-EDGYFEVKLPVKDGQNLL-----LIKDGDQTLEA 1155
DSM20072            1109 TYKKNAKLKFNDVEAES-------D-EDGYFEVKLPVKDGQNLL-----LIKDGDQTLEA 1155
KCCM34717           1107 TYKKNAKLKFNDVEAES-------D-EDGYFEVKLPVKDGQNLL-----LIKDGDQTLEA 1153
KCTC3034            1107 TYKKNAKLKFNDVEAES-------D-EDGYFEVKLPVKDGQNLL-----LIKDGDQTLEA 1153
MAG_rmk202_ldel     1109 TYKKNAKLKFNDVEAES-------D-EDGYFEVKLPVKDGQNQL-----LIKDGDQTLEA 1155
NWC_1_2             1109 TYKKNAKLKFNDVEAES-------D-EDGYFEVKLPVKDGQNQL-----LIKDGDQTLEA 1155
ND02                1109 TYKKNAKLKFNDVEAGS-------D-KNGYFEVKLPVKDGQNQL-----LIKDGDQILEA 1155
SK11                1146 TYPAAVDGTYTDAQGKKHDLNTTYDAATNSFTASMPVTNADYAAQVDLYADKAHTQLLKH 1205

2038                1156 VNFTVKAEGPKVSVDEERSGRILAKDDSYTLSGTVSGLAESGKLELTNLSDKSKTYKLTV 1215
ACA-DC87            1157 VNFTVKAEGPKVSVDEERSGRILAKDDSYTLSGTVSGLGESGKLELTNLSDKSKTNKLTV 1216
ATCC11842           1156 VNFTVKAEGPKVSVDEERSGRILAKDDSYTLSGTVSGLGESGKLELTNLSDKSKTDKLTV 1215
ATCC-BAA-365        1156 VNFTVKAEGPKVSVDEERSGRILAKDDSYTLSGTVSGLGESGKLELTNLSDKSKTNKLTV 1215
DSM20080            1158 VNFTVKAEGPKVSVDEERSGRILAKDDSYTLSGTVSGLGESGKLELTNLSDKSKTNKLTV 1217
KLDS1.0207          1158 VNFTVKAEGPKVSVDEERSGRILAKDDSYTLSGTVSGLGESGKLELTNLSDKSKTNKLTV 1217
KLDS1.1011          1156 VNFTVKAEGPKVSVDKESSGRILAKDDSYTLSGTVSGLGESGKLELTNLSDKSKTNELTV 1215
LBA-40              1156 VNFTVKAEGPKVSVDEERSGRILAKDDSYTLSGTVSGLGESGKLELTNLSDKSKTDKLTV 1215
LDB-C1              1158 VNFTVKAEGPKVSVDEERSGRILAKDDSYTLSGTVSGLGESGKLELTNLSDKSKTNKLTV 1217
L99                 1156 VNFTVKAEGPKVSVDEERSGRILAKDDSYTLSGTVSGLGESGKLELTNLSDKSKTDKLTV 1215
LJJ                 1156 VNFTVKAEGPKVSVDKESSGRILAKDDSYTLSGTVSGLGESGKLELTNLSDKSKTNELTV 1215
MN-BM-F01           1156 VNFTVKTEGPKVSVDEERSGRILAKDDSYTLSGTVSGLGESGKLELTNLSDKSKTDKLTV 1215
ND04                1156 VNFTVKAEGPKVSVDEERSGRILAKDDSYTLSGTVSGLGESGELELTNLSDKSKTNKLTV 1215
VHProbi-R03         1156 VNFTVKAEGPKVSVDEERSGRILAKDDSYTLSGTVSGLGESGKLELTNLSDKSKTNKLTV 1215
ATCC9649            1090 VNFTVKAEGPKVSVDEESSGRILAKDDSYTLSGTVSGLGESGKLELTNLSDKSKT-KLTV 1148
TUA4408L            1156 VSFTVKAEGPKVSVDEESSGRILAKDDSYTLSGTVSGLGESGKLELTNLSDKSKT-ELTV 1214
CIDCA133            1156 VNFTVKAEGPKVSVDEESSGRILAKDDSYTLSGTVSGLGESGKLELTNLSDKSKT-KLTV 1214
CRL581              1156 VNFTVKAEGPKVSVDEESSGRILAKDDSYTLSGTVSGLGESGKLELTNLSDKSKT-KLTV 1214
DSM20072            1156 VNFTVKAEGPKVSVDEESSGRILAKDDSYTLSGTVSGLGESGKLELTNLSDKSKT-KLTV 1214
KCCM34717           1154 VNFTVKAEGPKVSVDEESSGRILAKDDSYTLSGTVSGLGESGKLELTNLSDKSKT-KLTV 1212
KCTC3034            1154 VNFTVKAEGPKVSVDEESSGRILAKDDSYTLSGTVSGLGESGKLELTNLSDKSKT-KLTV 1212
MAG_rmk202_ldel     1156 VNFTVKAEGPKVSVDEESSGRILAKDDSYTLSGTVSGLGESGKLELTNLSDKSKT-KLTV 1214
NWC_1_2             1156 VNFTVKAEGPKVSVDEESSGRILAKDDSYTLSGTVSGLGESGKLELTNLSDKSKT-KLTV 1214
ND02                1156 VNFTVKAEGPKVSVDEESSGRILAKDDSYTLSGTVSGLGESGKLELTNLSDKSKT-KLTV 1214
SK11                1206 FDTKVRLTAPTFTDLKFNNGSDQTSEATIKVTGTVSADTKT-----VNVGD---T-VAAL 1256
```

```
2038               1216 DQDGKFSQKVDLKYGDNPFELTATDADGNVTKKDVTIFTARSYTYNKDMLTFDNIAS-DL 1274
ACA-DC87           1217 DQDGKFSQKVDLNYGDNPFELTATDADGNVTKKDVTIFTARSYTYNKDMLTFDNIAS-DL 1275
ATCC11842          1216 DQDGKFSQKVDLNYGDNPFELTATDADGNVTKKDVTIFTARSYTYNKDMLTFDNIAS-DL 1274
ATCC-BAA-365       1216 DQDGKFSQKVDLNYGDNPFELTATDADGNVTKKDVTIFTARSYTYNKDMLTFDNIAS-DL 1274
DSM20080           1218 DQDGKFSQKVDLNYGDNPFELTATDADGNVTKKDVTIFTARSYTYNEDMLTFDNIAS-DL 1276
KLDS1.0207         1218 DQDGKFSQKVDLNYGDNPFELTATDADGNVTKKDVTIFTARSYTYNEDMLTFDNIAS-DL 1276
KLDS1.1011         1216 DQDGKFSQKVDLNYGDNPFELTATDADGNVTKKDVTIFTARSYTYNKDMLTFDNIAS-DL 1274
LBA-40             1216 DQDGKFSQKVDLNYGDNPFELTATDADGNVTKKDVTIFTARSYTYNKDMLTFDNIAS-DL 1274
LDB-C1             1218 DQDGKFSQKVDLNYGDNPFELTATDADGNVTKKDVTIFTARSYTYNEDMLTFDNIAS-DL 1276
L99                1216 DQDGKFSQKVDLNYGDNPFELTATDADGNVTKKDVTIFTARSYTYNKDMLTFDNIAS-DL 1274
LJJ                1216 DQDGKFSQKVDLNYGDNPFELTATDADGNVTKKDVTIFTARSYTYNKDMLTFDNIAS-DL 1274
MN-BM-F01          1216 DQDGKFSQKVDLNYGDNPFELTATDADGNVTKKDVTIFTARSYTYNKDMLTFDNIAS-DL 1274
ND04               1216 DQDGKFSQKVDLNYGDNPFELTATDADGNVTKKDVTIFTARSYTYNKDMLTFDNIAS-DL 1274
VHProbi-R03        1216 DQDGKFSQKVDLNYGDNPFELTATDADGNVTKKDVTIFTARSYTYNKDMLTFDNIAS-DL 1274
ATCC9649           1149 DQDGKFSQKVDLNYGDNPFELTATDADGNVTKKDVTIFTARSYTYTKDMLTFDNIAS-NL 1207
TUA4408L           1215 DQNGKFSQKVDLNYGDNPFELTATDADGNVTKKDVTIFTARSYTYNKDMLTFDNIAS-DL 1273
CIDCA133           1215 DQHGKFSQKVNLNYGDNPFELTATDADGNVTNKDVTIFTARSYTYNKDMLTFDNVAS-DL 1273
CRL581             1215 DQDGKFSQKVDLNYGDNPFELTATDADGNVTKKDVTIFTARSYTYTKDMLTFDNIAS-DL 1273
DSM20072           1215 DQDGKFSQKVDLNYGDNPFELTATDADGNVTKKDVTIFTARSYTYTKDMLTFDNIAS-DL 1273
KCCM34717          1213 DQDGKFSQKVDLNYGDNPFELTATDADGNVTKKDVTIFTARSYTYTKDMLTFDNIAS-DL 1271
KCTC3034           1213 DQDGKFSQKVDLNYGDNPFELTATDADGNVTKKDVTIFTARSYTYTKDMLTFDNIAS-DL 1271
MAG_rmk202_ldel    1215 DQDGKFSQKVDLNYGDNPFELTATDADGNVTKKDVTIFTARSYTYTKDMLTFDNIAS-DL 1273
NWC_1_2            1215 DQDGKFSQKVDLNYGDNPFELTATDADGNVTKKDVTIFTARSYTYTKDMLTFDNIAS-DL 1273
ND02               1215 DQHGKFSQKVNLNYGDNPFELTATDADGNVTNKDVTIFTARSYTYNKDMLTFDNVAS-DL 1273
SK11               1257 DAQHHFSVDVPVNYGDNTIKVTATDEDGNTTTEQKTITSSYDPDMLKNSVTFDQGVTFGA 1316


2038               1275 TVI-GKTTPGYDEKDHSFTVSGKLAYPVARFQLNGDDVKYDPDTLKFSYTIKDLKNGNHS 1333
ACA-DC87           1276 TVI-GKTTPGYDEKDHSFTVTGKLAYPVARFQLNGDDIKYDPDTLKFSYTIKDLKNGNHS 1334
ATCC11842          1275 TVI-GKMTPGYDEKDHSFTVTGKLAYPVARFQLNGDDVKYDPDTLKFSYTIKDLKNGNHS 1333
ATCC-BAA-365       1275 TVI-GKTTPDYDEKDHSFTVTGKLAYPVARFQLNGDDVKYDPDTLKFSYTIKDLKNGNHS 1333
DSM20080           1277 TVI-GKTTPGYDEKDHSFTVTGKLAYPVARFQLNGDDVKYDPDTLKFSYTIKDLKNGNHS 1335
KLDS1.0207         1277 TVI-GKTTPGYDEKDHSFTVTGKLAYPVARFQLNGDDVKYDPDTLKFSYTIKDLKNGNHS 1335
KLDS1.1011         1275 TVI-GKTTPGYDEKDHSFTVTGKLAYPVARFQLNGDDVKYDPDTFKFSYTIKDLKNGNHS 1333
LBA-40             1275 TVI-GKTTPGYDEKDHSFTVTGKLAYPVARFQLNGDDVKYDPDTLKFSYTIKDLKNGNHS 1333
LDB-C1             1277 TVI-GKTTPGYDEKDHSFTVTGKLAYPVARFQLNGDDVKYDPDTLKFSYTIKDLKNGNHS 1335
L99                1275 TVI-GKMTPGYDEKDHSFTVTGKLAYPVARFQLNGDDVKYDPDTLKFSYTIKDLKNGNHS 1333
LJJ                1275 TVI-GKTTPGYDEKDHSFTVTGKLAYPVARFQLNGDDVKYDPDTLKFSYTIKDLKNGNHS 1333
MN-BM-F01          1275 TVI-GKTTPGYDEKDHSFTVTGKLAYPVARFQLNGDDVKYDPDTLKFSYTIKDLKNGNHS 1333
ND04               1275 TVI-GKTTPGYDEKDHSFTVTGKLAYPVARFQLNGDDVKYDPDTLKFSYTIKDLKNGNHS 1333
VHProbi-R03        1275 TVI-GKTTPGYDEKDHSFTVTGKLAYPVARFQLNGDDVKYDPDTLKFSYTIKDLKNGNHS 1333
ATCC9649           1208 TVI-GKTTPGYDEKDHSFTVTGKLAYPVARFQLNGDDVKYDPDTLKFSYTIKDLKNGNHS 1266
TUA4408L           1274 TVI-GKMTPGYDEKNHSFTVTGKLAYPVARFQLNGDDVKYDPDTLKFSYTIKDLKNGNNS 1332
CIDCA133           1274 TVI-GKTTPGYDEKDHSFTVTGKLAYPVARFQLNGDDVKYDPDTLKFSYTIKDLKNGNHS 1332
CRL581             1274 TII-GKTTPGYDEKDHSFTVTGKLAYPVARFQLNGDDVKYDPDTLKFSYTIKDLKNGNHP 1332
DSM20072           1274 TII-GKTTPGYDEKDHSFTVTGKLAYPVARFQLNGDDVKYDPDTLKFSYTIKDLKNGNHP 1332
KCCM34717          1272 TII-GKTTPGYDEKDHSFTVTGKLAYPVARFQLNGDDVKYDPDTLKFSYTIKDLKNGNHP 1330
KCTC3034           1272 TII-GKTTPGYDEKDHSFTVTGKLAYPVARFQLNGDDVKYDPDTLKFSYTIKDLKNGNHP 1330
MAG_rmk202_ldel    1274 TVI-GKTTPGYDEKDHSFTVTGKLAYPVARFQLNGDDVKYDPDTLKFSYTIKDLKNGNHP 1332
NWC_1_2            1274 TVI-GKTTPGYDEKDHSFTVTGKLAYPVARFQLNGDDVKYDPDTLKFSYTIKDLKNGNHP 1332
ND02               1274 TVI-GKTTPGYDEKDHSFTVTGKLAYPVARFQLNGDDVKYDPDTLKFSYTIKDLKNGNHS 1332
SK11               1317 NEFNATSAKFYDPKTGIATITGKVKHPTTTLQVDGKQIPIK-DDLTFSFTLDLGTLGQKP 1375
```

```
2038              1334 LTALVQDPRLNDGKPVVEWGYKLWVDLAAPSLQLEGMSLGEDGQLAVYTNKDVYDLKATI 1393
ACA-DC87          1335 LTALVQDPRLNDGKPVVEWGYKLWVDLAAPSLQLEGMSLGEDGQLAVYTNKDVYDLKATI 1394
ATCC11842         1334 LTALVQDPRLNDGKPVVEWGYKLWVDLAAPSLQLEGMSLGEDGQLAVYTNKDVYDLKATI 1393
ATCC-BAA-365      1334 LTALVQDPRLNDGKPVVEWGYKLWVDLAAPSLQLEGMSLGEDGQLAVYTNKDVYDLKATI 1393
DSM20080          1336 LTALVQDPRLNDGKPVVEWGYKLWVDLAAPSLQLEGMSLGEDGQLAVYTNKDVYDLKATI 1395
KLDS1.0207        1336 LTALVQDPRLNDGKPVVEWGYKLWVDLAAPSLQLEGMSLGEDGQLAVYTNKDVYDLKATI 1395
KLDS1.1011        1334 LTALVQDPRLNDGKPVVEWGYKLWVDLAAPSLQLEGMSLGEDGQLAVYTNKDVYDLKATI 1393
LBA-40            1334 LTALVQDPRLNDGKPVVEWGYKLWVDLAAPSLQLEGMSLGEDGQLAVYTNKDVYDLKATI 1393
LDB-C1            1336 LTALVQDPRLNDGKPVVEWGYKLWVDLAAPSLQLEGMSLGEDGQLAVYTNKDVYDLKATI 1395
L99               1334 LTALVQDPRLNDGKPVVEWGYKLWVDLAAPSLQLEGMSLGEDGQLAVYTNKDVYDLKATI 1393
LJJ               1334 LTALVQDPRLNDGKPVVEWGYKLWVDLAAPSLQLEGMSLGEDGQLAVYTNKDVYDLKATI 1393
MN-BM-F01         1334 LTALVQDPRLNDGKPVVEWGYKLWVDLAAPSLQLEGMSLGEDGQLAVYTNKDVYDLKATI 1393
ND04              1334 LTALVQDPRLNDGKPVVEWGYKLWVDLAAPSLQLEGMSLGEDGQLAVYTNKDIYDLKATI 1393
VHProbi-R03       1334 LTALVQDPRLNDGKPVVEWGYKLWVDLAAPSLQLEGMSLGEDGQLAVYTNKDVYDLKATI 1393
ATCC9649          1267 LTALVQDPRLNDGKPVVEWGYKLWVDLAAPSLQLEGMSLGEDGQLAVYTNKDVYDLKATI 1326
TUA4408L          1333 LTALVQDPRLNDGKPVVEWGYKLWVDLAAPSLQVEGMSLGEDGQLAVYTNKDVYDLKATI 1392
CIDCA133          1333 LTALVQDPRLNDGKPVVEWGYKLWVDLTAPSLQLEGMSLGEDGQLAVYTNKDVYDLKATI 1392
CRL581            1333 LTALVQDPRLNDGKPVVEWGYKLWVDLTAPSLQLEGMSLGEDGQLAVYTNKDVYDLKATI 1392
DSM20072          1333 LTALVQDPRLNDGKPVVEWGYKLWVDLTAPSLQLEGMSLGEDGQLAVYTNKDVYDLKATI 1392
KCCM34717         1331 LTALVQDPRLNDGKPVVEWGYKLWVDLTAPSLQLEGMSLGEDGQLAVYTNKDVYDLKATI 1390
KCTC3034          1331 LTALVQDPRLNDGKPVVEWGYKLWVDLTAPSLQLEGMSLGEDGQLAVYTNKDVYDLKATI 1390
MAG_rmk202_ldel   1333 LTALVQDPRLNDGKPVVEWGYKLWVDLTAPSLQLEGMSLGEDGQLAVYTNKDVYDLKATI 1392
NWC_1_2           1333 LTALVQDPRLNDGKPVVEWGYKLWVDLTAPSLQLEGMSLGEDGQLAVYTNKDVYDLKATI 1392
ND02              1333 LTALVQDPRLNDGKPVVEWGYKLWVDLTAPSLQLEGMSLGEDGQLAVYTNKDVYDLKATI 1392
SK11              1376 FGVVVGDTTQNK---TFQEALTFILDAVAPTLSLDSS-----TDAPVYTNDPNFQITGTA 1427
```

```
2038              1394 NDNLSGYSLQVG-SDTAYQDKTYKVFNEDFFKNRDAVKVSYPIKAEKDGFRKVKVTLTDG 1452
ACA-DC87          1395 NDNLSGYSLQVG-SDTAYQDKTYKVFNEDFFKNRDAVKVSYPIKAEKDGFRKVKVTLTDG 1453
ATCC11842         1394 NDNLSGYSLQVG-SDTAYQDKTYKVFNEDFFKNRDAVKVSYPIKAEKDGFRKVKVTLTDG 1452
ATCC-BAA-365      1394 NDNLSGYSLQVG-SDTAYQDKTYKVFNEDFFKNRDAVKVSYPIKAEKDGFRKVKVTLTDG 1452
DSM20080          1396 NDNLSGYSLQVG-SDTAYQDKTYKVFNEDFFKNRDAVKVSYPIKAEKDGSRKVKVTLTDG 1454
KLDS1.0207        1396 NDNLSGYSLQVG-SDTAYQDKTYKVFNEDFFKNRDAVKVSYPIKAEKDGSRKVKVTLTDG 1454
KLDS1.1011        1394 NDNLSGYSLQVG-SDTAYQDKTYKVFNEDFFKNRDAVKVSYPIKAEKDGFRKVKVTLTDG 1452
LBA-40            1394 NDNLSGYSLQVG-SDTAYQDKTYKVFNEDFFKNRDAVKVSYPIKAEKDGFRKVKVTLTDG 1452
LDB-C1            1396 NDNLSGYSLQVG-SDTAYQDKTYKVFNEDFFKNRDAVKVSYPIKAEKDGFRKVKVTLTDG 1454
L99               1394 NDNLSGYSLQVG-SDTAYQDKTYKVFNEDFFKNRDAVKVSYPIKAEKDGFRKVKVTLTDG 1452
LJJ               1394 NDNLSGYSLQVG-SDTAYQDKTYKVFNEDFFKNRDAVKVSYPIKAEKDGFRKVKVTLTDG 1452
MN-BM-F01         1394 NDNLSGYSLQVG-SDTAYQDKTYKVFNEDFFKNRDAVKVSYPIKAEKDGFRKVKVTLTDG 1452
ND04              1394 NDNLSGYSLQVG-SDTAYQDKTYKVFNEDFFKNRDAVKVSYPIKAEKDGFRKVKVTLTDG 1452
VHProbi-R03       1394 NDNLSGYSLQVG-SDTAYQDKTYKVFNEDFFKNRDAVKVSYPIKAEKDGFRKVKVTLTDG 1452
ATCC9649          1327 NDNLSGYSLQVG-SDTAYQDKTYEVFNEDFFKNRDAVKVSYPIKAEKDGSRKVKVTLTDE 1385
TUA4408L          1393 NDNLSGYSLQVG-SDTAYQDKTYEVFNEDFFKNRDAVKVSYPVKAEKDGSRKVKVTLTDE 1451
CIDCA133          1393 NDNLSGYSLQVG-SDTAYQDKSYEVFNEDFFKNRDAVKVSYPIKAEKDGSRKVKVTLTDS 1451
CRL581            1393 NDNLSGYSLQVG-SDTAYQDKTYEVFNEDFFKNRDAVKVSYPIKAEKDGSRKVKVTLTDS 1451
DSM20072          1393 NDNLSGYSLQVG-SDTAYQDKTYEVFNEDFFKNRDAVKVSYPIKAEKDGSRKVKVTLTDS 1451
KCCM34717         1391 NDNLSGYSLQVG-SDTAYQDKTYEVFNEDFFKNRDAVKVSYPIKAEKDGSRKVKVTLTDS 1449
KCTC3034          1391 NDNLSGYSLQVG-SDTAYQDKTYEVFNEDFFKNRDAVKVSYPIKAEKDGSRKVKVTLTDS 1449
MAG_rmk202_ldel   1393 NDNLSGYSLQVG-SDTAYQDKTYEVFNEDFFKNRDAVKVSYPIKAEKDGSRKVKVTLTDS 1451
NWC_1_2           1393 NDNLSGYSLQVG-SDTAYQDKTYEVFNEDFFKNRDAVKVSYPIKAEKDGSRKVKVTLTDS 1451
ND02              1393 NDNLSGYSLQVG-SDTAYQDKSYEVFNEDFFKNRDAVKVSYPIKAEKDGSRKVKVTLTDS 1451
SK11              1428 TDNAQYLSLSINGSSVASQ---YVDININSGKP-GHMAIDQPVKL-LEGKNVLTVAVTDS 1482
```

```
2038              1453 SDNKTEQDFILYN-HQADLEAPEVSASESKKTNQAVQLKVGNLSDVQKSAGKFKAADLYY 1511
ACA-DC87          1454 SDNKTEQDFILYN-HQADLEAPEVSASESKKTNQAVQLKVGNLSDVQKSAGKFKAADLYY 1512
ATCC11842         1453 SDNKTEQDFTLYN-HQADLEAPEVSASESKKTNQAVQLKVGNLSDVQKSAGKFKAADLYY 1511
ATCC-BAA-365      1453 SDNKTEQDFILYN-HQADLEAPEVSASESKKTNQAVQLKVGNLSDVQKSAGKFKAADLYY 1511
DSM20080          1455 SDNKTEQDFTLYN-HQADLEAPEVSASESKKTNQAVQLKVGNLSDVQKSAGKFKAADLYY 1513
KLDS1.0207        1455 SDNKTEQDFTLYN-HQADLEAPEVSASESKKTNQAVQLKVGNLSDVQKSAGKFKAADLYY 1513
KLDS1.1011        1453 SDNKTEQDFILYN-HQADLEAPEVSASESKKTNQAVQLKVGNLSDVQKSAGKFKAADLYY 1511
LBA-40            1453 SDNKTEQDFTLYN-HQADLEAPEVSASESKKTNQAVQLKVGNLSDVQKSAGKFKAADLYY 1511
LDB-C1            1455 SDNKTEQDFTLYN-HQADLEAPEVSASESKKTNQAVQLKVGNLSDVQKSAGKFKTADLYY 1513
L99               1453 SDNKTEQDFTLYN-HQADLEAPEVSASESKKTNQAVQLKVGNLSDVQKSAGKFKAADLYY 1511
LJJ               1453 SDNKTEQDFTLYN-HQADLEAPEVSASESKKTNQAVQLKVGNLSDVQKSAGKFKAADLYY 1511
MN-BM-F01         1453 SDNKTEQDFTLYN-HQADLEAPEVSASESKKTNQAVQLKVGNLSDVQKSAGKFKAADLYY 1511
ND04              1453 SDNKTEQDFTLYN-HQADLEAPEVSASESKKTNQAVQLKVGNLSDVQKSAGKFKAADLYY 1511
VHProbi-R03       1453 SDNKTEQDFTLYN-HQADLEAPEVSASESKKTNQAVQLKVGNLSDVQKSAGKFKAADLYY 1511
ATCC9649          1386 SDNKTEQDFTLYN-HQADLEAPEVSASESKKTNQAVQLKVENLSDVQKSAGKFKAADLYY 1444
TUA4408L          1452 SDNKTEQDFTLYN-HQADLEAPEVSASESKKTNQAVQLKVENLSDVQKSAGKFKAADLYY 1510
CIDCA133          1452 SDNETEQDFTLYN-HQADLEAPEVSASESKKTNQAVQLKVENLSDVQKSAGKFKAADLYY 1510
CRL581            1452 SDNKTEQDFTLYN-HQADLEAPEVSASESKKTNQAVQLKVENLSDVQKSAGKFKAADLYY 1510
DSM20072          1452 SDNKTEQDFTLYN-HQADLEAPEVSASESKKTNQAVQLKVENLSDVQKSAGKFKAADLYY 1510
KCCM34717         1450 SDNKTEQDFTLYN-HQADLEAPEVSASESKKTNQAVQLKVENLSDVQKSAGKFKAADLYY 1508
KCTC3034          1450 SDNKTEQDFTLYN-HQADLEAPEVSASESKKTNQAVQLKVENLSDVQKSAGKFKAADLYY 1508
MAG_rmk202_ldel   1452 SDNKTEQDFTLYN-HQADLEAPEVSASESKKTNQAVQLKVENLSDVQKSAGKFKVADLYY 1510
NWC_1_2           1452 SDNKTEQDFTLYN-HQADLEAPEVSASESKKTNQAVQLKVENLSDVQKSAGKFKVADLYY 1510
ND02              1452 SDNETEQDFTLYN-HQADLEAPEVSASESKKTNQAVQLKVENLSDVQKSAGKFKAADLYY 1510
SK11              1483 EDNTTTKNITVYYEPKKTLAAPTVTPSTTEPA-KTVTLTANS-------AA--TGETVQY 1532
```

```
2038              1512 SVD-GKTWTKLDKDTVQVAENGKVEFKYQDVYGNESKVTTYEVKNIVKEVAAQPEL---- 1566
ACA-DC87          1513 SVD-GKTWTKLDKDTVQVAENGKVEFKYQDVYGNESKVTTYEVKNIVKEVAAQPEL---- 1567
ATCC11842         1512 SVD-GKTWTKLDKDTVQVAENGKVEFKYQDVYGNESKVTTYEVKNIVKEVAAQPEL---- 1566
ATCC-BAA-365      1512 SVD-GKTWTKLDKDTVQVAENGKVEFKYQDVYGNESKVTTYEVKNIVKEVAAQPEL---- 1566
DSM20080          1514 SVD-GKTWTKLDKDTVQVAENGKVEFKYQDVYGNESKVTTYEVKNIVKEVAAQPEL---- 1568
KLDS1.0207        1514 SVD-GKTWTKLDKDTVQVAENGKVEFKYQDVYGNESKVTTYEVKNIVKEVAAQPEL---- 1568
KLDS1.1011        1512 SVD-GKTWTKLDKDTVQVAENGKVEFKYQDVYGNESKVTTYEVKNIVKEVAAQPEL---- 1566
LBA-40            1512 SVD-GKTWTKLDKDTVQVAENGKVEFKYQDVYGNESKVTTYEVKNIFKEVAAQPEL---- 1566
LDB-C1            1514 SVD-GKTWTKLDKDTVQVAENGKVEFKYQDVYGNESKVTTYEVKNIVKEVAAQPEL---- 1568
L99               1512 SVDDGKTWTKLDKDTVQVAENGKVEFKYQDVYGNESKVTTYEVKNIVKEVAAQPEL---- 1567
LJJ               1512 SVD-GKTWTKLDKDTVQVAENGKVEFKYQDVYGNESKVTTYEVKNIVKEVAAQPEL---- 1566
MN-BM-F01         1512 SVD-GKTWTKLDKDTVQVAENGKVEFKYQDVYGNESKVTTYEVKNIVKEVVAQPEL---- 1566
ND04              1512 SVD-GKTWTKLDKDTVQVAENGKVEFKYQDVYGNESKVTTYEVKNIVKEVAAQPEL---- 1566
VHProbi-R03       1512 SVD-GKTWTKLDKDTVQVAENGKVEFKYQDVYGNESKVTTYEVKNIVKEVAAQPEL---- 1566
ATCC9649          1445 SVD-GKTWTKLDKETVQVAENGKVEFKYQDVYGNESKVTTYEVKNIVKEVAAQPEL---- 1499
TUA4408L          1511 SVD-GKTWTKLDKDTVQVAENGKVEFKYQDVYGNESKVTTCEVKNIVKEVAAQPEL---- 1565
CIDCA133          1511 SVD-GKTWTKLDQDTVQVAENGKVEFKYQDVYGNESKVTTYEVKNIVKEVAAQPEL---- 1565
CRL581            1511 SVD-GKTWTKLDKDTVQVAENGKVEFKYQDVYGNESKVTTYEVKNIVKEVAAQPEL---- 1565
DSM20072          1511 SVD-GKTWTKLDKDTVQVAENGKVEFKYQDVYGNESKVTTYEVKNIVKEVAAQPEL---- 1565
KCCM34717         1509 SVD-GKTWTKLDKDTVQVAENGKVEFKYQDVYGNESKVTTYEVKNIVKEVAAQPEL---- 1563
KCTC3034          1509 SVD-GKTWTKLDKDTVQVAENGKVEFKYQDVYGNESKVTTYEVKNIVKEVAAQPEL---- 1563
MAG_rmk202_ldel   1511 SVD-GKTWTKLDKDTVQVAENGKVEFKYQDVYGNESKVTTYEVKNIVKEVAAQPEL---- 1565
NWC_1_2           1511 SVD-GKTWTKLDKDTVQVAENGKVEFKYQDVYGNESKVTTYEVKNIVKEVAAQPEL---- 1565
ND02              1511 SVD-GKTWTKLDQDTVQVAENGKVEFKYQDVYGNESKVTTYEVKNIVKEVAAQPEL---- 1565
SK11              1533 SADGGKTYQDVPAAGVTVTANGTFKFKSTDLYGNESPAVDYVVTNIKADDPAQLQAAKQE 1592
```

```
2038              1567 --------------------KLTPDGEGKVKAELAFDKKDVDKDFNHIKYSLDGGKSWTD 1606
ACA-DC87          1568 --------------------KLTPDGEGKVKAVLAFDKKDVDKDFNHIKYSLDGGKSWTD 1607
ATCC11842         1567 --------------------KLTPDGEGKVKAELAFDKKDVDKDFNHIKYSLDGGKSWTD 1606
ATCC-BAA-365      1567 --------------------KLTPDGEGKVKAVLAFDKKDVDKDFNHIKYSLDGGKSWTD 1606
DSM20080          1569 --------------------KLTPDGEGKVKAELAFDKKDVDKDFNHIKYSLDGGKSWTD 1608
KLDS1.0207        1569 --------------------KLTPDGEGKVKAELAFDKKDVDKDFNHIKYSLDGGKSWTD 1608
KLDS1.1011        1567 --------------------KLTPDGEGKVKAVLAFDKKDVDKDFNHIKYSLDGGKSWTD 1606
LBA-40            1567 --------------------KLTPDGEGKVKAVLAFDKKDVDKDFNHIKYSLDGGKSWTD 1606
LDB-C1            1569 --------------------KLTPDGEGKVKAELAFDKKDVDKDFNHIKYSLDGGKSWTD 1608
L99               1568 --------------------KLTPDGEGKVKAELAFDKKDVDKDFNHIKYSLDGGKSWTD 1607
LJJ               1567 --------------------KLTPDGEGKVKAELAFDKKDVDKDFNHIKYSLDGGKSWTD 1606
MN-BM-F01         1567 --------------------KLTPDGEGKVKAELAFDKKDVDKDFNHIKYSLDGGKSWTD 1606
ND04              1567 --------------------KLTPDGEGKVKAELAFDKKDVDKDFNHIKYSLDGGKSWTD 1606
VHProbi-R03       1567 --------------------KLTPDGEGKVKAELAFDKKDVDKDFNHIKYSLDGGKSWTD 1606
ATCC9649          1500 --------------------KLTPDGEGKVKAELAFDKKDVDKDFNHIKYSLDGGKTWTD 1539
TUA4408L          1566 --------------------KLTPDGEGKVKAELAFDRKDVDKDFNHIKYSLDGGKTWTD 1605
CIDCA133          1566 --------------------KLTPDGEGKVKAELAFDKKDVDKNFNHIKYSLDGGKSWTD 1605
CRL581            1566 --------------------KLTPDGEGKVKAELAFDKKDVDQDFNHIKYSLDGGKTWTD 1605
DSM20072          1566 --------------------KLTPDGEGKVKAELAFDKKDVDQDFNHIKYSLDGGKTWTD 1605
KCCM34717         1564 --------------------KLTPDGEGKVKAELAFDKKDVDQDFNHIKYSLDGGKTWTD 1603
KCTC3034          1564 --------------------KLTPDGEGKVKAELAFDKKDVDQDFNHIKYSLDGGKTWTD 1603
MAG_rmk202_ldel   1566 --------------------KLTPDGEGKVKAELAFDKKDVDQDFNHIKYSLDGGKTWTD 1605
NWC_1_2           1566 --------------------KLTPDGEGKVKAELAFDKKDVDQDFNHIKYSLDGGKTWTD 1605
ND02              1566 --------------------KLTPDGEGKVKAELAFDKKDVDKNFNHIKYSLDGGKSWTD 1605
SK11              1593 LTNLIASAKTLSASGKYDDATTTALAAATQKAQTALDQTNASVD---------------- 1636

2038              1607 YKDAFTLTHNGTVEFKSYDDAGNESQVYTSVVKSNTKSDVKSDGNGEAKSDNKSSAKDDK 1666
ACA-DC87          1608 YKDAFTLTHNGTVEFKSYDDAGNEGQVYTSVVKGNTKSDVKSDGNGEAKSDNKSSAKDDK 1667
ATCC11842         1607 YKDAFTLTHNGTVEFKSYDDAGNESQVYTSVVKVERKLPA-PDLTGTVEADKS------- 1658
ATCC-BAA-365      1607 YKDAFTLTHNGTVEFKSYDDAGNEGQVYTSVVKVERKLPA-PDLTGTVEADKS------- 1658
DSM20080          1609 YKDAFTLTHNGTVEFKSYDDAGNESQVYTSVVKVERKLPA-PDLTGTVEADKSV------ 1661
KLDS1.0207        1609 YKDAFTLTHNGTVEFKSYDDAGNESQVYTSVVKVERKLPA-PDLTGTVEADKSV------ 1661
KLDS1.1011        1607 YKDAFTLTHNGTVEFKSYDDAGNESQVYTSVVKVERKLPA-PDLTGTVEADKS------- 1658
LBA-40            1607 YKDAFTLTHNGTVEFKSYDDAGNEGQVYTSVVKVERKLPA-PDLTGTVEADKS------- 1658
LDB-C1            1609 YKDAFTLTHNGTVEFKSYDDAGNESQVYTSVVKVERKLPA-PDLTGTVEVDKSV------ 1661
L99               1608 YKDTFTLTHNGTVEFKSYDDAGNESQVYTSVVKVERKLPA-PDLTGTVEANKR------- 1659
LJJ               1607 YKDAFTLTHNGTVEFKSYDDAGNESQVYTSVVKVERKLPA-PDLTGTVEADKSV------ 1659
MN-BM-F01         1607 YKDAFTLTHNGTVEFKSYDDAGNESQVYTSVVKVERKLPA-PDLTGTVEADKSV------ 1659
ND04              1607 YKDAFTLTHNGTVEFKSYDDAGNESQVYTSVVKVERKLPA-PDLTGTVEADKSV------ 1659
VHProbi-R03       1607 YKDAFTLTHNGTVEFKSYDDAGNESQVYTSVVKVERKLPA-PDLTGTVEA---------- 1655
ATCC9649          1540 YKDAFTLTHNGTVEFKSYDNAGNESQVYTSVVKVERKLPA-PDLTGTVETDKSV------ 1592
TUA4408L          1606 YKDAFTLTHNGTVEFKSYDDAGNESQVYTSVVKVERKLPA-PDLTGTVEADKSV------ 1658
CIDCA133          1606 YKDAFTLTHNGTVEFKSYDDAGNESQVYTSVVKVERKLPA-PDLTSTVEADKNV------ 1658
CRL581            1606 YKDAFTLTHNGTVEFKSYDDAGNESQVYTSVVKVERKLPA-PDLTGTVEADKSV------ 1658
DSM20072          1606 YKDAFTLTHNGTVEFKSYDDAGNESQVYTSVVKVERKLPA-PDLTGTVEADKSV------ 1658
KCCM34717         1604 YKDAFTLTHNGTVEFKSYDDAGNESQVYTSVVKVERKLPA-PDLTGTVEADKSV------ 1656
KCTC3034          1604 YKDAFTLTHNGTVEFKSYDDAGNESQVYTSVVKVERKLPA-PDLTGTVEADKSV------ 1656
MAG_rmk202_ldel   1606 YKDAFTLTHNETVEFKSYDDAGNESQVYTSVVKVERKLPA-PDLTGTVEADKSV------ 1658
NWC_1_2           1606 YKDAFTLTHNETVEFKSYDDAGNESQVYTSVVKVERKLPA-PDLTGTVEADKSV------ 1658
ND02              1606 YKDAFTLTHNGTVEFKSYDDAGNESQVYTSVVKVERKLPA-PDLTSTVEADKNV------ 1658
SK11              1637 ------------------SLTGANRDLQTAINQLAAKLPA-DKKTSLLNQLQSV------ 1671
```

```
2038              1667 KTDSKTTSK--------------------------------------------------- 1675
ACA-DC87          1668 KTDSKTTSK--------------------------------------------------- 1676
ATCC11842         1659 -VEVKA-GNVEQAEKDADGKVTLLYSTD-------------------------------- 1684
ATCC-BAA-365      1659 -VEVKA-GN--------------------------------------------------- 1665
DSM20080          1662 --EVKA-GNVEQAEKDADGKVTLLYSTD-------------------------------- 1686
KLDS1.0207        1662 --EVKA-GNVEQAEKDADGKVTLLYSTD-------------------------------- 1686
KLDS1.1011        1659 -VEVK-AGN--------------------------------------------------- 1665
LBA-40            1659 -VEVKA-GN--------------------------------------------------- 1665
LDB-C1            1662 --EVKA-GNVEQAEKDADGKVTLLYSTD-------------------------------- 1686
L99               1660 ------------------------------------------------------------ 1659
LJJ               1660 --EVKA-GNVEQAEKD-------------------------------------------- 1672
MN-BM-F01         1660 --EVKA-GNVEQAEKDADGKVTLLYSTD-------------------------------- 1684
ND04              1660 --EVKA-GNVEQAEKDADGKVTLLYSTD-------------------------------- 1684
VHProbi-R03       1656 ------------------------------------------------------------ 1655
ATCC9649          1593 --EVKA-GNVEQAEKDADGKVTLLYSTD-------------------------------- 1617
TUA4408L          1659 --EVKA-GNVEQAEKDTDGKVTLLYSTD-------------------------------- 1683
CIDCA133          1659 --EVKA-GNVEQAEKDADGKVTLLYSTD-------------------------------- 1683
CRL581            1659 --EVKA-GNVEQAEKDADGKVTLLYSTD-------------------------------- 1683
DSM20072          1659 --EVKA-GNVEQAEKDADGKVTLLYSTD-------------------------------- 1683
KCCM34717         1657 --EVKA-GNVEQAEKDADGKVTLLYSTD-------------------------------- 1681
KCTC3034          1657 --EVKA-GNVEQAEKDADGKVTLLYSTD-------------------------------- 1681
MAG_rmk202_ldel   1659 --EVKA-GNVEQAEKDADGKVTLLYSTD-------------------------------- 1683
NWC_1_2           1659 --EVKA-GNVEQAEKDADGKVTLLYSTD-------------------------------- 1683
ND02              1659 --EVKA-GNVEQAEKDADGKVTLLYSTD-------------------------------- 1683
SK11              1672 --KAAL-GTDLGNQTDSSTGKTFTAALDDLVAQAQAGTQTDDQLQATLAKVLDAVLAKLA 1728


2038              1676 ------------------------------------------------------------ 1675
ACA-DC87          1677 ------------------------------------------------------------ 1676
ATCC11842         1685 -------------------GKDWSKVDGEVKLTDSGRAMFKYRDGDD---------NESA 1716
ATCC-BAA-365      1666 ------------------------------------------------------------ 1665
DSM20080          1687 -------------------GKDWSKVDGEVKLTDSGRAMFKYRDGDD---------NESA 1718
KLDS1.0207        1687 -------------------GKDWSKVDGEVKLTDSGRAMFKYRDGDD---------NESA 1718
KLDS1.1011        1666 ------------------------------------------------------------ 1665
LBA-40            1666 ------------------------------------------------------------ 1665
LDB-C1            1687 -------------------GKDWSKVDGEVKLTDSGRAMFKYRDGDD---------NESA 1718
L99               1660 ------------------------------------------------------------ 1659
LJJ               1673 ------------------------------------------------------------ 1672
MN-BM-F01         1685 -------------------GKDWSKVDGEVKLTDSGRAMFKYRDGDD---------NESA 1716
ND04              1685 -------------------GKDWSKVDGEVKLTDSGRAMFKYRDGDD---------NESA 1716
VHProbi-R03       1656 ------------------------------------------------------------ 1655
ATCC9649          1618 -------------------GKDWSKVDGEVKLTDSDRAMFKYRDGDD---------NESA 1649
TUA4408L          1684 -------------------GKDWSKVDGEVKLTDSDRAMFKYRDGDD---------NESA 1715
CIDCA133          1684 -------------------GKDWSKVDGEVKLTDSDRAMFKYRDGDG---------NESA 1715
CRL581            1684 -------------------GKDWSKVDGEVKLTDSDRAMFKYRDGDD---------NESA 1715
DSM20072          1684 -------------------GKDWSKVDGEVKLTDSDRAMFKYRDGDD---------NESA 1715
KCCM34717         1682 -------------------GKDWSKVDGEVKLTDSDRAMFKYRDGDD---------NESA 1713
KCTC3034          1682 -------------------GKDWSKVDGEVKLTDSDRAMFKYRDGDD---------NESA 1713
MAG_rmk202_ldel   1684 -------------------GKDWSKVDGEVKLTDSDRAMFKYRDGDD---------NESA 1715
NWC_1_2           1684 -------------------GKDWSKVDGEVKLTDSDRAMFKYRDGDD---------NESA 1715
ND02              1684 -------------------GKDWSKVDGEVKLTDSDRAMFKYRDGDG---------NESA 1715
SK11              1729 EGIKAATPAEVGNAKDAATGKTWYA-DIADTLT-SGQASADASDKLAHLQALQSLKTKVA 1786
```

```
2038              1676 ------------------------------------------------------------ 1675
ACA-DC87          1677 ------------------------------------------------------------ 1676
ATCC11842         1717 VVVYEVKKVV-------------EEKTAEFKPENKDDSKSDSKS------------DSKS 1751
ATCC-BAA-365      1666 ------------------------------------------------------------ 1665
DSM20080          1719 VVVYEVKKVV-------------EEKTAEFKPENKDDIKSDSKS------------DSKS 1753
KLDS1.0207        1719 VVVYEVKKVV-------------EEKTAEFKPENKDDIKSDSKS------------DSKS 1753
KLDS1.1011        1666 ------------------------------------------------------------ 1665
LBA-40            1666 ------------------------------------------------------------ 1665
LDB-C1            1719 VVVYEVKKVV-------------EEKTAEFKPENKDDSKSDSKS------------DSKS 1753
L99               1660 ------------------------------------------------------------ 1659
LJJ               1673 ------------------------------------------------------------ 1672
MN-BM-F01         1717 VVVYEVKKVV-------------EEKTAEFKPENKDDSKSDSKS------------DSKS 1751
ND04              1717 VVVYEVKKVV-------------EEKTAEFKPENKDDSKS-------------------- 1743
VHProbi-R03       1656 ------------------------------------------------------------ 1655
ATCC9649          1650 VVVYEVKKVV-------------EEKTAESKPENKDDSKSDSNTDTKSDTKSDSKSDSKS 1696
TUA4408L          1716 VVVYEVKKVV-------------EEKTAESKPENKDDSKSNSNTDTKSDTKSDSKSDSKS 1762
CIDCA133          1716 VVVYEVKKVV-------------EEKAAVSKPENKDDNKSDGKNETKSDNKSDGKSDVKT 1762
CRL581            1716 VVVYEVKKVV-------------EEKTAESKPENKDDSKSDSNTDTKSDTKSDSKSDSKS 1762
DSM20072          1716 VVVYEVKKVV-------------EEKTAESKPENKDDSKSDSNTDTKSDTKSDSKSDSKS 1762
KCCM34717         1714 VVVYEVKKVV-------------EEKTAESKPENKDDSKSDSNTDTKSDTKSDSKSDSKS 1760
KCTC3034          1714 VVVYEVKKVV-------------EEKTAESKPENKDDSKSDSNTDTKSDTKSDSKSDSKS 1760
MAG_rmk202_ldel   1716 VVVYEVKKVV-------------EEKTAESKPENKDDSKSDSNTDTKSDTKSDSKSDSKS 1762
NWC_1_2           1716 VVVYEVKKVV-------------EEKTAESKPENKDDSKSDSNTDTKSDTKSDSKSDSKS 1762
ND02              1716 VVVYEVKKVV-------------EEKAAVSKPENKDDNKSDGKNETKSDNKSDGKSDVKT 1762
SK11              1787 AAVEAAKTVGKGDGTTGTSDKGGGQGTPAPAPGDTGKDKGDEGS------------QPSS 1834

2038              1676 ------------------------------------------------------------ 1675
ACA-DC87          1677 ------------------------------------------------------------ 1676
ATCC11842         1752 DNK------------AETKSDVKTD----------------TKSDVKSDGNGEA------ 1777
ATCC-BAA-365      1666 ------------------------------------------------------------ 1665
DSM20080          1754 D----------------------------------------------------------- 1754
KLDS1.0207        1754 D----------------------------------------------------------- 1754
KLDS1.1011        1666 ------------------------------------------------------------ 1665
LBA-40            1666 ------------------------------------------------------------ 1665
LDB-C1            1754 D----------------------------------------------------------- 1754
L99               1660 ------------------------------------------------------------ 1659
LJJ               1673 ------------------------------------------------------------ 1672
MN-BM-F01         1752 DNK------------AETKSDVKTDTKSDSKSDN----KSNTKSDVKSDGNGEA------ 1789
ND04              1744 ------------------------------------------------------------ 1743
VHProbi-R03       1656 ------------------------------------------------------------ 1655
ATCC9649          1697 DNK------------AETKSDVKTDSKTDTKSDSKSDNKSNTKSDVKSDGNGEA------ 1738
TUA4408L          1763 DNK------------VETKSDVKTDSKTDTESDSKSD---NTKSDVKSDGNGEA------ 1801
CIDCA133          1763 DSKTDTKSDAKSDAKADTKSDTKSDSKT------------DSKNDFKSNGKSEA------ 1804
CRL581            1763 DNK------------AETKSDV-------------------------------------- 1772
DSM20072          1763 DNK------------AETKSDV-------------------------------------- 1772
KCCM34717         1761 DNK------------AETKSDV-------------------------------------- 1770
KCTC3034          1761 DNK------------AETKSDV-------------------------------------- 1770
MAG_rmk202_ldel   1763 DNK------------AETKSDV-------------------------------------- 1772
NWC_1_2           1763 DNK------------AETKSDV-------------------------------------- 1772
ND02              1763 DSKTDTKSDAKSDAKADTKSDTKSDSKT------------DSKNDFKSNGKSEA------ 1804
SK11              1835 GGN------------IPTKPATTTSTTTDDTTDR----NGQLTSGTSDKGGGQGTPAPAP 1878
```

```
2038                1676 ----------------------------SSAKKTSAKKNKKASKKASKKTVKKTKTYKK 1706
ACA-DC87            1677 ----------------------------SSAKKTSAKKNKKASKKASKKTVKKTKTYKK 1707
ATCC11842           1778 ------KS--DNKPSAKDDKKTDSKTTSKSSAKKTSAKKNKKASKKASKKTVKKTKTYKK 1829
ATCC-BAA-365        1666 ----------------------------SSAKKTSAKKNKKASKKASKKTVKKTKTYKK 1696
DSM20080            1755 -----------NKSETKSDVKTDSKTTSKSSAKKTSAKKNKKASKKASKKTVKKTKTYKK 1803
KLDS1.0207          1755 -----------NKSETKSDVKTDSKTTSKSSAKKTSAKKNKKASKKASKKTVKKTKTYKK 1803
KLDS1.1011          1666 ----------------------------SSAKKTSAKKNKKASKKASKKTVKKTKTYKK 1696
LBA-40              1666 ----------------------------SSAKKTSAKKNKKASKKASKKTVKKTKTYKK 1696
LDB-C1              1755 -----------NKAETKSDVKTDSKTTSKSSAKKTSAKKNKKASKKASKKTVKKTKTYKK 1803
L99                 1660 -------------SSAKDDKKTDSKTTSKSSAK--------------------KTKTYKK 1686
LJJ                 1673 ----------------------------------------------KKASKKTKTYKK 1684
MN-BM-F01           1790 ------KS--DNKPSAKDDKKTDSKTTSKSSAKKTSAKKNKKASKKASKKTVKKTKTYKK 1841
ND04                1744 ---------------------DSKTTSKSSAKKTSAKKNKKASKKASKKTVKKTKTYKK 1781
VHProbi-R03         1656 ------------------------------------------------------------ 1655
ATCC9649            1739 ------KS--DNKSSAKDDVKTDSKTTSKSSAKKTSAKKNKKAS----KKTVKKTKTYKK 1786
TUA4408L            1802 ------KY--NNKSSAKDDKKTDGKTTSKSSAKKTNAKKNKKASKKASKKTVKKTKTYKK 1853
CIDCA133            1805 ------KS--NNKSSAKDDKKPKSNPASKSSTKKTSSKKNNKAS----KKASKNTKTY-- 1850
CRL581              1773 -------------------KTDSKTTSKSSAKKTSAKKNKKAS----KKTVKKTKTYKK 1808
DSM20072            1773 -------------------KTDSKTTSKSSAKKTSAKKNKKAS----KKTVKKTKTYKK 1808
KCCM34717           1771 -------------------KTDSKTTSKSSAKKTSAKKNKKAS----KKTVKKTKTYKK 1806
KCTC3034            1771 -------------------KTDSKTTSKSSAKKTSAKKNKKAS----KKTVKKTKTYKK 1806
MAG_rmk202_1del     1773 -------------------KTDSKTTSKSSAKKTSAKKNKKAS----KKTVKKTKTYKK 1808
NWC_1_2             1773 -------------------KTDSKTTSKSSAKKTSAKKNKKAS----KKTVKKTKTYKK 1808
ND02                1805 ------KS--NNKSSAKDDKKPKSNPASKSSTKKTSSKKNNKAS----KKASKNTKTY-- 1850
SK11                1879 GDIGKDKGDEGSQPSSGGNIPTNPATTTSTTTDDTTDRNGQLTSG---KGALPKTGE--- 1932

2038                1707 VKLTKLTKVYNKKGKVVGKLKKKTSIKLLSKKQKLHGKYYYRVGKNRYILASNLPKKTKK 1766
ACA-DC87            1708 VKLTKLTKVYNKKGKVVGKLKKKTSIKLLSKKQKLHGKYYYRVGKNRYILASNLPKKTKK 1767
ATCC11842           1830 VKLTKLTKVYNKKGKVVGKLKKKTSVKLLSKKQKLHGKYYYRVGKNRYILASSLPKKTKK 1889
ATCC-BAA-365        1697 VKLTKLTKVYNKKGKVVGKLKKKTSIKLLSKKQKLHGKYYYRVGKNRYILASNLPKKTKK 1756
DSM20080            1804 VKLTKLTKVYNKKGKVVGKLKKKTSVKLLSKKQKLHGKYYYRVGKNRYILASSLPKKTKK 1863
KLDS1.0207          1804 VKLTKLTKVYNKKGKVVGKLKKKTSVKLLSKKQKLHGKYYYRVGKNRYILASSLPKKTKK 1863
KLDS1.1011          1697 VKLTKLTKVYNKKGKVVGKLKKKTSIKLLSKKQKLHGKYYYRVGKNRYILASNLPKKTKK 1756
LBA-40              1697 VKLTKLTKVYNKKGKVVGKLKKKTSIKLLSKKQKLHGKYYYRVGKNRYILASNLPKKTKK 1756
LDB-C1              1804 VKLTKLKKVYNKKGKVVGKLKEKTSVKLLSKKQKLHGKYYYRVGKNRYILASSLPKKTKK 1863
L99                 1687 VKLTKLTKVYNKKGKVVGKLKKKTSVKLLSKKQKIHGKYYYRVGKNRYILASSLPKKTKK 1746
LJJ                 1685 VKLTKLKKVYNKKGKVVGKLKKKTSVKLLSKKQKLHGKYYYRVGKNRYILASSLPKKTKK 1744
MN-BM-F01           1842 VKLTKLTKVYNKKGKVVGKLKKKTSVKLLSKKQKLHGKYYYRVGKNRYILASSLPKKTKK 1901
ND04                1782 VKLTKLTKVYNKKGKVVGKLKK-------------------------------------- 1803
VHProbi-R03         1656 ------TKVYNKKGKVVGKLKKKTSVKLLSKKQKLHGKYYYRVGKNRYILASSLPKKTKK 1709
ATCC9649            1787 VKLTKLTKVYNKKGKVVGKLK-KTSVKLLSKKQKLHGKYYYRVGKNRYILASSLPKKTKK 1845
TUA4408L            1854 VKLTKLTKVYNKKGKVVGKLK-KTNVKLLSKKQKLHGKYYYRVGKNRYILASSLPKKTKK 1912
CIDCA133            1851 --MTKLTKVFNKKGKVVGKLKKKISVKLLSKKQKLHGKYYYRIGKNKYVLASSLPKKTKK 1908
CRL581              1809 VKLTKLTKVYNKKGKVVGKLK-KTSVKLLSKKQKLHGKYYYRVGKNRYILASSLPKKTKK 1867
DSM20072            1809 VKLTKLTKVYNKKGKVVGKLK-KTSVKLLSKKQKLHGKYYYRVGKNRYILASSLPKKTKK 1867
KCCM34717           1807 VK-------------LVGKLK-KTSVKLLSKKQKLHGKYYYRVGKNRYILASSLPKKTKK 1852
KCTC3034            1807 VK-------------LVGKLK-KTSVKLLSKKQKLHGKYYYRVGKNRYILASSLPKKTKK 1852
MAG_rmk202_1del     1809 VKLTKLTKVYNKKGKVVGKLK-KTSVKLLSKKQKLHGKYYYRVGKNRYILASSLPKKTKK 1867
NWC_1_2             1809 VKLTKLTKVYNKKGKVVGKLK-KTSVKLLSKKQKLHGKYYYRVGKNRYILASSLPKKTKK 1867
ND02                1851 --MTKLTKVFNKKGKVVGKLKKKISVKLLSKKQKLHGKYYYRIGKNKYVLASSLPKKTKK 1908
SK11                1933 ---TTERPAFGFLGVIVVSL--------M------------------------------- 1950
```

```
2038              1767 VKQVRARKNAKVYNKKGKVVDHLKKKQKVKLLSKKQKLHGKYYYRIGKNRYVNANVL 1823
ACA-DC87          1768 VKQVRARKNAKVYNKKGKVVGHLKKKQKVKLLSKKQKLHGKYYYRIGKNRYVNANVL 1824
ATCC11842         1890 VKQVRARKNAKVYNKKGKVVGHLKKKQKVKLLSKKQKLHGKYYYRIGKNRYVNANVL 1946
ATCC-BAA-365      1757 VKQVRARKNAKVYNKKGKVVGHLKKKQKVKLLSKKQKLHGKYYYRIGKNRYVNANVL 1813
DSM20080          1864 VKQVRARKNAKIYNKKGKVVGHLKKKQKVKLLSKKQKLHGKYYYRIGKNRYVNANVL 1920
KLDS1.0207        1864 VKQVRARKNAKIYNKKGKVVGHLKKKQKVKLLSKKQKLHGKYYYRIGKNRYVNANVL 1920
KLDS1.1011        1757 VKQVRARKNAKVYNKKGKVVGHLKKKQKVKLLSKKQKLHGKYYYRIGKNRYVNANVL 1813
LBA-40            1757 VKQVRARKNAKVYNKKGKVVGHLKKKQKVKLLSKKQKLHGKYYYRIGKNRYVNANVL 1813
LDB-C1            1864 VKQVRARKNAKVYNKKGKVVGHLKKKQKVKLLSKKQKLHGKYYYRIGKNRYVNANVL 1920
L99               1747 VKQVRARKNAKVYNKKGKVVDHLKKKQKVKLLSKKQKLHGKYYYRIGKNRYVNANVL 1803
LJJ               1745 VKQVRARKNAKVYNKKGKVVGHLKKKQKVKLLSKKQKLHGKYYYRIGKNRYVNANVL 1801
MN-BM-F01         1902 VKQVRARKNAKVYNKKGKVVGHLKKKQKVKLLSKKQKLHGKYYYRIGKNRYVNANVL 1958
ND04              1804 -------------------------KTSVKLLSKKQKLHGKYYYRIGKNRYVNANVL 1835
VHProbi-R03       1710 VKQVRARKNAKVYNKKGKVVGHLKKKQKVKLLSKKQKLHGKYYYRIGKNRYVNANVL 1766
ATCC9649          1846 VKQVRARKNAKVYNKKGKVVGHLKKKQKVKLLSKKQKLHGKYYYRIGKNRYVKANVL 1902
TUA4408L          1913 VKQVRSRKNAKVYNKKGKVVGHLKKKQKVKLLSKKQKLHGKYYYRIGKNRYVKANVL 1969
CIDCA133          1909 VKQVRVRKNAKVYNKKGKVVGHLKKKQKVKLLSKKQKLHGKYYYRIGKNRYVKANVL 1965
CRL581            1868 VKQVRVRKNAKAYNKKGKVVGHLKKKQKVKLLSKKQKLHGKYYYRIGKNRYVKANVL 1924
DSM20072          1868 VKQVRVRKNAKAYNKKGKVVGHLKKKQKVKLLSKKQKLHGKYYYRIGKNRYVKANVL 1924
KCCM34717         1853 VKQVRVRKNAKAYNKKGKVVGHLKKKQKVKLLSKKQKLHGKYYYRIGKNRYVKANVL 1909
KCTC3034          1853 VKQVRVRKNAKAYNKKGKVVGHLKKKQKVKLLSKKQKLHGKYYYRIGKNRYVKANVL 1909
MAG_rmk202_ldel   1868 VKQVRVRKNAKAYNKKGKVVGHLKKKQKVKLLSKKQKLHGKYYYRIGKNRYVKANVL 1924
NWC_1_2           1868 VKQVRVRKNAKAYNKKGKVVGHLKKKQKVKLLSKKQKLHGKYYYRIGKNRYVKANVL 1924
ND02              1909 VKQVRVRKNAKVYNKKGKVVGHLKKKQKVKLLSKKQKLHGKYYYRIGKNRYVKANVL 1965
SK11              1951 -----------------G-VLGLKRKQREE--------------------------- 1962
```

Supplementary Figure S4. Amino acid sequence alignment was generated by T-Coffee for the Prt proteinases from 24 *L. delbrueckii* strains. The proteinase PrtP of *Lactococcus lactis* subsp. *cremoris* SK11 was used as an outgroup. Conserved regions are marked in black, substitutions are marked in white, and conservative substitutions are marked in gray. The replacement of serine by cysteine at position 282 of PrtB in comparison to PrtL is highlighted in yellow.

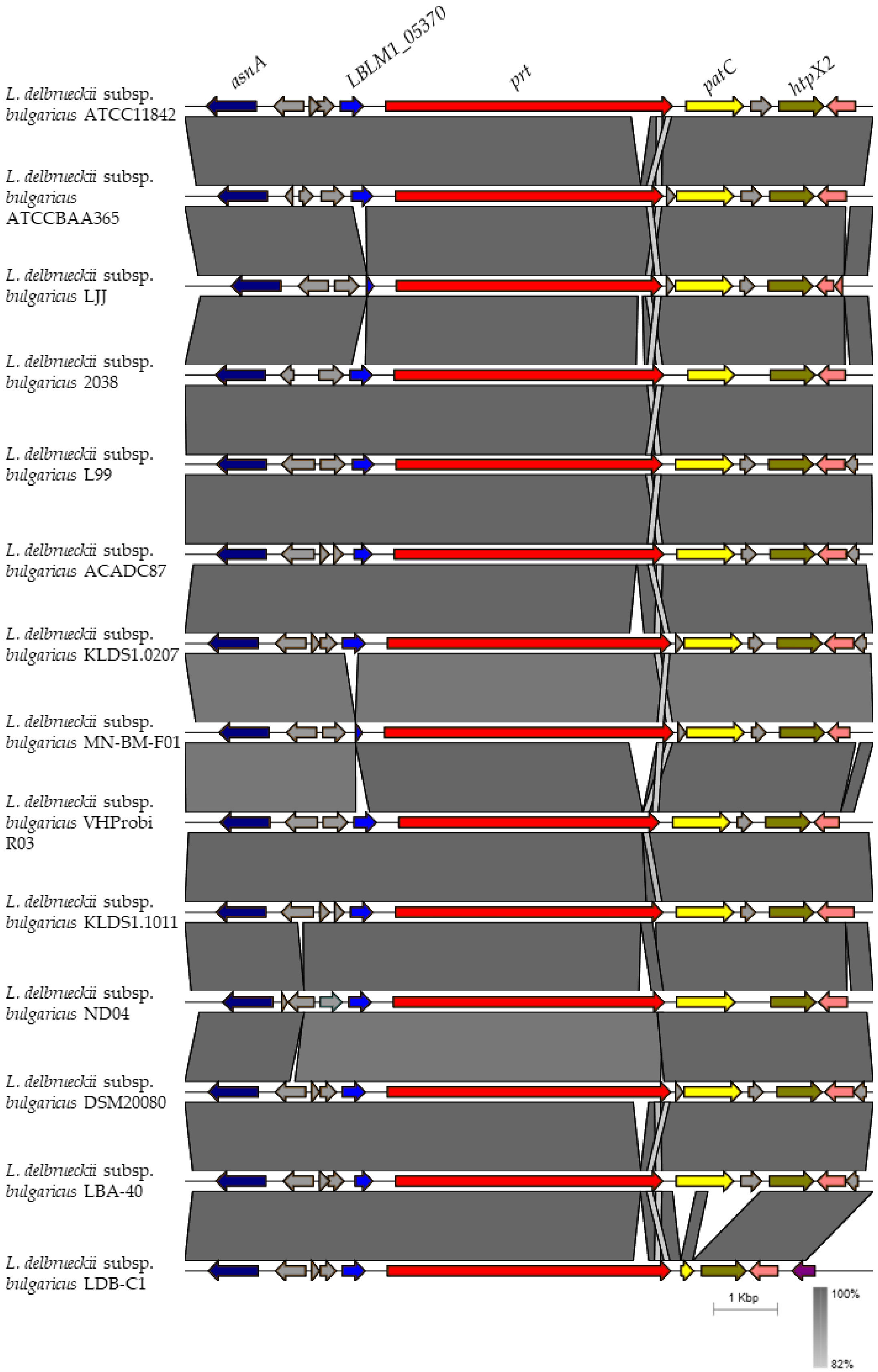

Supplementary Figure S5. Synteny comparison of the *prt* region in *L. delbrueckii* subsp. *bulgaricus* strains (14 Kb). The following genes are represented: aspartate ammonium lyase *asnA* (dark blue), acetyltransferase *LBLM1_05370* (blue), proteinase *prtB* (red), cystathionine beta-lyase *patC* (yellow), heat shock protein *htpX2* (green), and hypothetical proteins (gray).

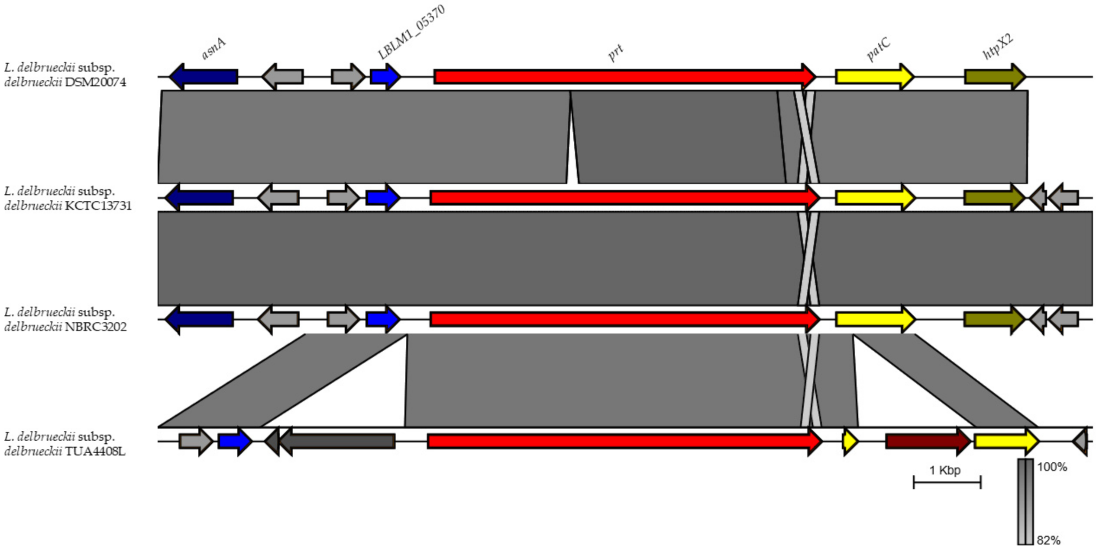

Supplementary Figure S6. Synteny comparison of the *prt* region in *L. delbrueckii* subsp. *delbrueckii* strains (14 Kb). The following genes are represented: aspartate ammonium lyase *asnA* (dark blue), acetyltransferase *LBLM1_05370* (blue), proteinase *prtL* (red), cystathionine beta-lyase *patC* (yellow), heat shock protein *htpX2* (green), and hypothetical proteins (gray).

A)

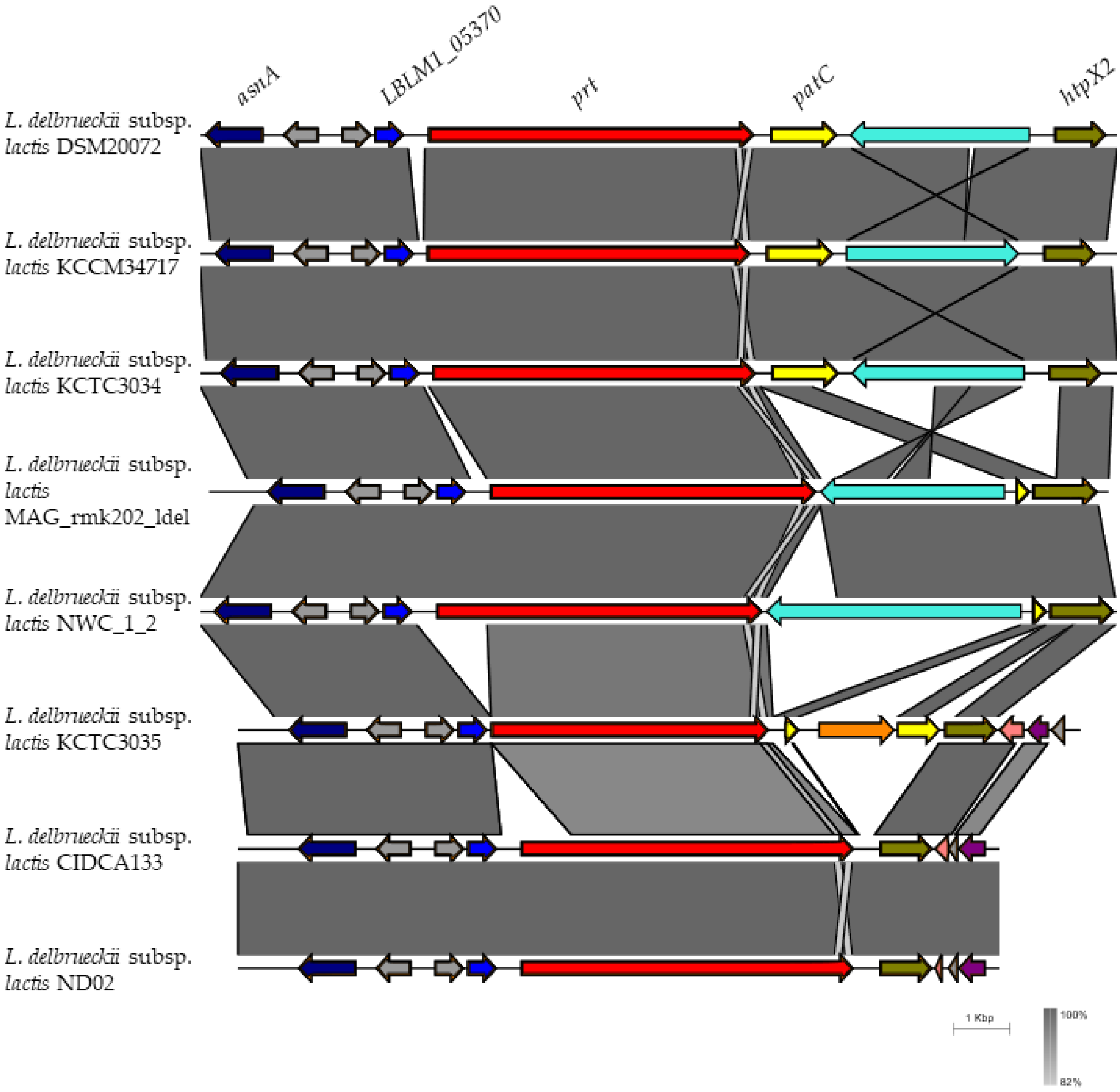

B)

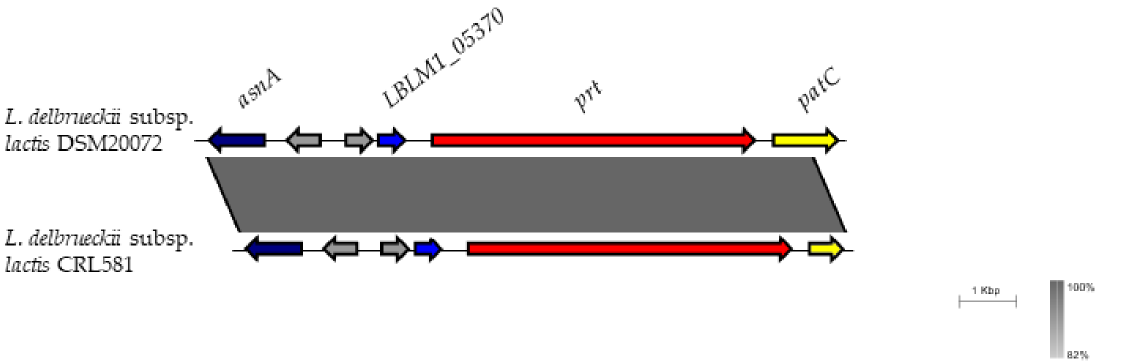

Supplementary Figure S7. (A) Synteny comparison of the *prt* region in *L. delbrueckii* subsp. *lactis* strains (14 Kb). The following genes are represented: aspartate ammonium lyase *asnA* (dark blue), acetyltransferase *LBLM1_05370* (blue), proteinase *prtL* (red), cystathionine beta-lyase *patC* (yellow), heat shock protein *htpX2* (green), and hypothetical proteins (gray). Insertion sequences are marked in white. **(B)** Synteny comparison of the *prtL* region between *L. delbrueckii* subsp. *lactis* DSM20072 and *L. delbrueckii* subsp. *lactis* CRL581 in the *prtL* region.
